# Anti-obesity effects of Wumeishanzhayin: an integrated lipidomics and transcriptomics study

**DOI:** 10.3389/fphar.2025.1697683

**Published:** 2025-10-21

**Authors:** Xiao Huang, Chunling Liao, Bo Peng, Jin Wang, Yong Zhang, Qingman He, Sijie Dang, Mei Zhao

**Affiliations:** ^1^ Hospital of Tradition Medicine LS.SC, Leshan, Sichuan Province, China; ^2^ College of Computer Science, Chengdu University, Chengdu, Sichuan, China; ^3^ Institute of Traditional Chinese Medicine, Sichuan Academy of Chinese Medicine Sciences, Chengdu, Sichuan, China; ^4^ Chengdu University of Traditional Chinese Medicine, Chengdu, Sichuan, China

**Keywords:** Wumeishanzhayin, high-fat and high-fructose diet, obesity, transcriptomics, lipidomics, AMPK/CPT-1

## Abstract

**Background:**

The global prevalence of adult obesity has increased significantly, affecting over 890 million adults worldwide. Wumeishanzhayin (WMSZY), a formulation derived from four medicinal and edible botanical drugs, has shown efficacy in alleviating obesity and lipid metabolism disorders induced by high-fat and high-fructose (HFHF) diets. However, its underlying therapeutic mechanisms remain unclear.

**Objective:**

This study aims to investigate the therapeutic effects and mechanisms of WMSZY in ameliorating obesity induced by a HFHF diet.

**Methods:**

UPLC-MS/MS was used to characterize the metabolites of WMSZY. The effects of WMSZY on body weight gain, lipid metabolism, and glucose dysfunction were evaluated in a HFHF diet-induced murine model. Targeted lipidomics and transcriptomics analyses were performed to identify differentially altered lipids (DALs) and differentially expressed genes (DEGs). Computational approaches, including molecular docking simulation and dynamics simulations, were used to predict bioactive compound-target interactions. Key findings were validated through RT-qPCR and Western blot. Integrated transcriptomic and lipidomic analyses identified potential therapeutic targets.

**Results:**

Sixteen metabolites were identified in WMSZY. Animal studies showed that WMSZY reduced body weight gain, improved lipid and glucose metabolism, and alleviated inflammation in HFHF diet-induced mice. Targeted lipidomics indicated that WMSZY’s anti-obesity effect may be linked to cholesterol metabolism, adipocyte lipolysis, lipid digestion, insulin resistance, and glycerolipid metabolism. Transcriptomic analysis suggested involvement of AMPK, MAPK, and PPAR signaling pathways. Molecular docking simulation revealed strong binding of WMSZY metabolites with AMPK, and simulations showed that cryptochlorogenic acid had notable binding affinity. The data from Western blot and RT-qPCR showed changes associated with the activation of the AMPK/CPT-1 pathway and the inhibition of lipid metabolism-related genes following WMSZY treatment.

**Conclusion:**

WMSZY significantly reduces body weight gain and ameliorates glucose and lipid metabolic disorders in HFHF diet-induced mice. Its anti-obesity effect is likely due to the regulation of lipid metabolism *via* the AMPK/CPT-1 signaling pathway.

## Graphical Abstract

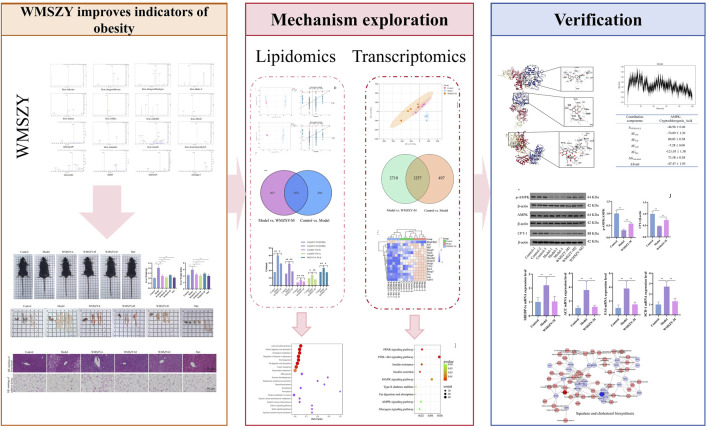

## Introduction

Obesity has become a global public health challenge. Since 1975, the prevalence of adult obesity (BMI ≥30) has doubled, affecting over 890 million adults (13% of the global population), and is projected to rise to 1.02 billion (18% of adults) by 2030 (Lingvay et al., 2024). Obesity is a major risk factor for various chronic diseases, leading to metabolic disorders such as type 2 diabetes, fatty liver disease, and hyperlipidemia, as well as cardiovascular conditions including hypertension (Gilden et al., 2024). The core mechanism of obesity involves a long-term imbalance between energy intake (excessive diet) and energy expenditure (insufficient physical activity), resulting in surplus energy stored as triglycerides in adipose tissue (González-Muniesa et al., 2017). Overeating induces adipose tissue dysfunction, characterized by adipocyte hypertrophy, low-grade inflammation, and insulin resistance, which exacerbate metabolic disturbances (González-Muniesa et al., 2017). Moreover, dysbiosis of the gut microbiota can influence energy absorption and inflammatory responses, while epigenetic modifications and social-environmental factors further contribute to the development of obesity (Fabricatore and Wadden, 2006). Current interventions mainly rely on lifestyle management, glucagon-like peptide-1 (GLP-1) receptor agonists, and metabolic surgery, yet these approaches face limitations such as gastrointestinal side effects, weight rebound after drug withdrawal, surgical trauma, and high costs (Elmaleh-Sachs et al., 2023). In contrast, traditional Chinese medicine (TCM) exhibits unique advantages through holistic regulation.

TCM has accumulated thousands of years of practical experience in health maintenance and disease treatment, forming a distinctive theoretical system and therapeutic approach. Its core intervention, the Chinese herbal formula, combines multiple medicinal plants in a scientifically designed composition to achieve synergistic efficacy and reduced toxicity. Existing studies have demonstrated that many classical prescriptions exert multi-target regulatory effects on obesity, including modulation of lipid metabolism disorders, inhibition of inflammatory cascades, mitigation of oxidative stress, and regulation of pathological processes such as autophagy and apoptosis (Chen et al., 2023; Gong et al., 2020; Shang et al., 2024; Wang Z. et al., 2024). Wumeishanzhayin (WMSZY) is an important herbal formula composed mainly of *Prunus mume (Sieb.) Sieb. et Zucc.* (Wumei), *Citrus reticulata Blanco* (Chenpi), *Crataegus cuneata Sieb. et Zucc.* (Shanzha), and *Glycyrrhiza glabra L.* (Gancao). Modern pharmacological studies have shown that Wumei, Chenpi, and Gancao exhibit anti-inflammatory, antioxidant, and metabolism-modulating properties, helping reduce tissue damage (Jian et al., 2022; Ruiqiang and Changfu, 2023; Yue et al., 2022). Moreover, Shanzha improves high-fat diet-induced lipid metabolism disorders through multiple mechanisms, including inhibition of key enzymes in cholesterol biosynthesis, suppression of pre adipocyte differentiation, and promotion of bile acid excretion. Research has shown that WMSZY could effectively control weight gain and lipid metabolism disorders induced by a high-fat diet (Wang C. et al., 2024). Therefore, investigating whether WMSZY can alleviate obesity and metabolic disorders, along with its underlying mechanisms, is both theoretically and clinically meaningful.

## Methods

### Preparation and analysis of WMSZY

The WMSZY was prepared using four botanical drugs in a 6:6:2:1 ratio, corresponding to the following respective weights: *P. mume* (Siebold) Siebold and Zucc. [Rosaceae; Mume Fructus] (30 g), *Crataegus pinnatifida* Bunge [Rosaceae; Crataegi Fructus](30 g), *C. reticulata* Blanco [Rutaceae; Citri Reticulatae Pericarpium] (10 g), and *Glycyrrhiza uralensis* Fisch. ex DC. [Fabaceae; Glycyrrhizae Radix et Rhizoma] (5 g). All botanical drugs met the specifications of the Pharmacopoeia of the People’s Republic of China. The mixed botanical drugs were soaked in water for 20 min, decocted for 60 min, and then filtered. After a second decoction with water and subsequent filtration, the two filtrates were combined, concentrated by rotary evaporation at 60 °C, and lyophilized. The resulting powder (eeach Gram of the resulting lyophilized powder was equivalent to 5.17 g of the crude crude drug) was stored at 4 °C for later use.

UPLC-MS/MS analysis was performed using an ExionLC™ AD system coupled with a QTRAP^®^ 6500+ mass spectrometer (SCIEX). Flavonoid identification: The thawed samples were mixed with the internal standard (daidzein; IsoReag, 97% purity), with other analytes quantified by external standard method, and 70% methanol, then sonicated for 30 min and centrifuged. The supernatant was filtered through a 0.22 μm membrane. Chromatographic separation was achieved on a Waters HSS T3 C18 column (1.8 μm, 100 × 2.1 mm) with a mobile phase consisting of 0.05% formic acid in water (A) and acetonitrile (B). The gradient elution program was: 0 min, 90:10 (A/B); 1 min, 80:20; 9 min, 30:70; 12.5 min, 5:95; 13.6 min, 90:10; 15 min, 90:10. Flow rate was 0.35 mL/min, and the column temperature was 40 °C. Mass spectrometry conditions included an ESI source at 550 °C, positive (+5500 V) and negative ion modes (−4500 V), and curtain gas at 35 psi. Organic acid identification: Sample were extracted with 20% acetonitrile-methanol, centrifuged, and the supernatant was precipitated at −20 °C, followed by another centrifugation prior to injection. Chromatographic conditions were the same as those for flavonoids, with the gradient adjusted as follows: 0 min, 95:5 (A/B); 8–9.5 min, 5:95; 9.6–12 min, 95:5. Mass spectrometry parameters were identical to those used for flavonoid analysis. All eetabolites were detected by MetWare (http://www.metware.cn/) based on the AB Sciex QTRAP 6500 LC-MS/MS platform.

### Animals, drugs and reagents

A total of 36 male C57BL/6J mice, aged 4–5 weeks and weighing 18–20 g, were used in this study. All animals were obtained from SPF (Beijing) Biotechnology Co., Ltd. (license No. SCXX [Beijing] 2019-0010). The experiment was approved by the Ethics Committee of Chengdu University of Traditional Chinese Medicine (approval No. 202312) to ensure compliance with ethical standards. During the study, mice were housed under controlled environmental conditions (temperature 22 °C–24 °C, humidity 40%–60%) with a consistent light-dark cycle. Food and water were provided *ad libitum* to maintain animal welfare and ensure reliable experimental outcomes. The high-fat and high-fructose (HFHF) diet (D09100310; Rodent Diet containing 40 kcal% fat, 20 kcal% fructose, and 2% cholesterol) was purchased from Research Diets, USA. Metformin was purchased from Merck Pharmaceutical (Jiangsu, China).

The total cholesterol (TC) colorimetric assay kit (No. E-BC-K109-M), triglyceride (TG) colorimetric assay kit (No. E-BC-K261-M), low-density lipoprotein cholesterol (LDL-C) colorimetric assay kit (No. E-BC-K205-M), high-density lipoprotein cholesterol (HDL-C) colorimetric assay kit (No. E-BC-K221-M), Mouse INS ELISA Kit (No. E-EL-M1382), Mouse TNF-α ELISA Kit (No. E-EL-M3063), and Mouse IL-1β ELISA Kit (No. E-EL-M0037) were all obtained from Wuhan Elabscience Biotechnology Co., Ltd. β-actin (No. 81115-1-RR) was purchased from Wuhan Sanying Biotechnology Co., Ltd., and p-AMPK (No. HA723603), AMPK (No. ET1608-40), and CPT1 (No. HA723261) were obtained from Hangzhou Huaan Biotechnology Co., Ltd.

### Animal experiment design

After 1 week of acclimatization, mice in the control group were fed a standard diet, while those in the other groups were fed the HFHF diet to establish an obesity model. After 12 weeks of feeding, one mouse was randomly selected from both the control group and the model control group to measure body weight and epididymal fat weight, and epididymal white adipose tissue (eWAT) was collected for HE staining. Following successful model establishment, the animals were randomly assigned using a random number table to the model group and various treatment groups, with six mice per group. Based on the human-to-mouse equivalent dose conversion, the medium-dose group received 2.2 g/kg (WMSZY-M), the high-dose group 4.4 g/kg (WMSZY-H), the low-dose group 1.1 g/kg (WMSZY-L), and the positive control group received metformin at 0.1 g/kg (Met). The control and model groups were administered an equal volume of normal saline by gavage. All groups received 10 mL/kg per dose, once daily, for four consecutive weeks. During the gavage period, the model and treatment groups continued to receive the HFHF diet.

### General condition

Mouse body weight was measured weekly. An oral glucose tolerance test (OGTT) was performed 1 day prior to tissue collection. Body weight of the mice was recorded, followed by anesthesia with sodium pentobarbital (60 mg/kg, i.p.) for blood collection *via* the retro-orbital sinus, and then cervical dislocation was performed to ensure rapid and humane euthanasia. Epididymal, interscapular, and inguinal adipose tissues and liver were dissected and weighed. The epididymal fat index, interscapular fat index, inguinal fat index, liver index, and Lee’s index were calculated. The formula was as follows: Epididymal/Interscapular/Inguinal Fat Index or Liver Index = Epididymal/Interscapular/Inguinal Fat or Liver (g)/Body Weight (g) × 100%. Lee’s Index $=\frac{\sqrt[{3}]{Body\;Weight\;\left(g\right)}}{Length\;from\;nose\;to\;anus\;\left(cm\right)}$ × 10^3^.

### ELISA biochemistry and ELISA

Serum biochemical analysis was performed using a fully automated biochemical analyzer after warming the serum to 37 °C. Matched reagents and calibrators were used, standard reaction parameters were set, and quality control samples were tested simultaneously. The analyzer automatically calculated and reported the concentrations of AST, ALT, LDL-C, HDL-C, T-CHO, and TG. ELISA procedures were conducted as follows: Microplates were set with standard, blank, and sample wells, to which diluted standards, diluent, or samples were added, followed by incubation at 37 °C. After discarding the liquid, 100 μL of biotinylated antibody was added to each well for further incubation. Plates were then washed three times. Subsequently, 100 μL of enzyme conjugate was added to each well and incubated at 37 °C for 30 min, followed by five washes using the same method. Then, 90 μL of TMB substrate solution was added to each well and incubated in the dark at 37 °C for approximately 15 min until a gradient appeared in the standard wells. Finally, 50 μL of stop solution was added to each well, and the optical density was immediately measured at 450 nm.

### HE staining

After fixation, liver and adipose tissues were thoroughly rinsed with distilled water and immersed in 75% ethanol overnight. The tissues were then subjected to a standard series of procedures, including dehydration, clearing, paraffin embedding, sectioning, and mounting. HE staining was subsequently performed to facilitate the observation of tissue architecture. Finally, pathological changes in the liver and adipose tissues of mice were examined, photographed, and analyzed under a light microscope.

### Lipidomics

The frozen samples stored at −80 °C were thawed on ice, and 10 mg of eWAT(epididymal White Adipose Tissue) was weighed and added to steel beads for homogenization using a ball mill (30 Hz, 20 s). The homogenate was then centrifuged at 4 °C (3,000 rpm, 30 s). A 1 mL extraction solvent (methyl tert-butyl ether: methanol, 3:1) (Matyash et al., 2008) was added, and the mixture was vortexed for 15 min. After adding 200 μL of water, the solution was vortexed again for 1 min and then centrifuged at 4 °C (12,000 rpm, 10 min). The supernatant was collected and nitrogen-evaporated to dryness. The residue was reconstituted in 400 μL acetonitrile: isopropanol (1:1) and centrifuged, and the supernatant was collected for analysis. Chromatographic conditions were as follows: Thermo Accucore™ C30 column (2.1 × 100 mm, 2.6 μm); mobile phase A (acetonitrile/water) and B (acetonitrile/isopropanol); gradient elution program: 0 min, A/B 80:20 (v/v); 2 min, 70:30 (v/v); 4 min, 40:60 (v/v); 9 min, 15:85 (v/v); 14 min, 10:90 (v/v); 15.5 min, 5:95 (v/v); 17.3 min, 5:95 (v/v); 17.5 min, 80:20 (v/v); 20 min, 80:20 (v/v); flow rate: 0.35 mL/min, column temperature: 45 °C, injection volume: 2 μL. Mass spectrometry conditions were as follows: ESI source (positive/negative ion mode, positive (+5500 V) and negative ion modes (−4500 V), temperature: 500 °C, Gas1: 45 psi, Gas2: 55 psi, MRM mode scan (optimized DP/Collision Energy parameters). Qualitative analysis was based on retention time and parent/child ion pairs using a self-built database (MWDB). Quantification was performed using the internal standard method and peak area integration in MRM mode. The original peak intensity data (*.wiff) from Analyst^®^ software (SCIEX, Framingham, MA, United States) were extracted using MultiQuant 3.0.3 software (SCIEX, Framingham, MA, United States). Peaks in the MRM chromatograms were extracted with the following parameters: Gaussian Smooth Width (0 points), RT Half Windows (30 s), Min. Peak Width (2 points), Min. Peak Height: (0), Baseline Noise Percentages (70%), Baseline Sub. Window (1.0 min), Peak Splitting (2 points). The minimum intensity for chromatographic peak filtering was 1,000 cps, the minimum signal-to-noise ratio was 5, and the retention time deviation was <0.2 min. Based on the peak areas of QC samples, substances with a detection rate less than 50% and a CV greater than 0.3 were removed, resulting in a final substance list (Supplementary Table S1). The Internal Standard Method involves adding a known amount of a pure substance as an internal standard (see Supplementary Table S2 for internal standards) to a known amount of the sample mixture. The sample containing the internal standard is then analyzed, and the peak areas of both the internal standard and the target analytes are measured along with their relative correction factors. The percentage content of the target analyte in the sample can be calculated using the formula. The above operations are carried out according to the standard procedures of Metware Biotech Co., Ltd, Wuhan, China. All related data analysis was performed on the dedicated MetWare cloud platform (https://cloud.metware.cn).

### Transcriptomics

The eWAT from the control group, model group, and the WMSZY-M group were collected. Sample quality was assessed using agarose gel electrophoresis, a Qubit 2.0 fluorometer, and an Agilent 2100 Bioanalyzer. mRNA was enriched with Oligo (dT) magnetic beads, followed by purification, end repair, and PCR amplification to construct the final cDNA libraries. Preliminary library quality was evaluated using Qubit 2.0 and Agilent 2,100, and the effective concentration was accurately quantified by qPCR (valid concentration >2 nM). Qualified libraries were sequenced on the Illumina platform. Raw reads were subjected to quality control using fastp, aligned to the reference genome using HISAT2, and gene expression levels were calculated as FPKM values with featureCounts v1.6.2. Differential expression analysis between groups was performed using DESeq2. The Benjamini–Hochberg method was applied to adjust *P* values for multiple testing to obtain the false discovery rate (FDR). DEGs were defined as those with |FC| ≥ 2, *P* < 0.05, and FDR <0.05. Finally, KEGG enrichment analysis was performed to assess the biological pathways associated with the differentially expressed genes. The above operations are carried out according to the standard procedures of Metware Biotech Co., Ltd, Wuhan, China. All related data analysis was performed on the dedicated MetWare cloud platform (https://cloud.metware.cn).

### Molecular Docking simulation

Protein crystal structures were obtained from the RCSB Protein Data Bank (http://www.rcsb.org/), with the AMPK protein file ID 5UFU. The three-dimensional structures of ligand molecules were retrieved from the PUBCHEM database (https://pubchem.ncbi.nlm.nih.gov/). Molecular docking simulation was performed using the CB-Dock2 platform (https://cadd.labshare.cn/cb-dock2/php/blinddock.php#job_list_load) (Liu et al., 2022).

### Molecular dynamics simulation

Molecular dynamics simulations were performed using Gromacs 2022.3. Small molecules were parameterized with the GAFF force field *via* AmberTools 22, and hydrogen addition and RESP charge calculations were carried out using Gaussian 16W, with the resulting data incorporated into the system topology file. Simulations were conducted at 300 K and 1 bar using the Amber99sb-ildn force field, with Tip3p water as the solvent and Na^+^ ions added to neutralize the system. The system first underwent energy minimization using the steepest descent method, followed by NVT and NPT equilibration, each for 100,000 steps (approximately 100 ps, coupling constant 0.1 ps). Subsequently, a production run of 5,000,000 steps was performed with a 2 fs time step, totaling 100 ns. Trajectories were analyzed using built-in tools to calculate RMSD, RMSF, radius of gyration, and binding free energy (MMGBSA).

### Western blotting

A total of 100 mg of tissue was homogenized in RIPA lysis buffer at ten times the tissue volume. After homogenization, lysis, and centrifugation, total protein extracts were collected, and protein concentrations were determined using a BCA assay. Proteins were separated by SDS-PAGE and transferred onto PVDF membranes under a constant current of 200 mA for 1 h. Membranes were blocked in TBST containing 5% skim milk for 1 h and then incubated with primary antibodies overnight at 4 °C on a shaker. After washing, membranes were incubated with secondary antibodies at 37 °C for 1 h. Following additional washes, chemiluminescent substrate was applied, and the signals were visualized by exposure and developed. Films were scanned and archived, and the optical density of target bands was analyzed using the AlphaEaseFC software system.

### RT-qPCR

Total RNA from eWAT was extracted using the Total RNA Miniprep Kit. cDNA was synthesized according to the instructions of the reverse transcription kit, followed by qPCR. The PCR cycling conditions were as follows: 95 °C for 5 min; 95 °C for 10 s; 60 °C for 30 s; and 65 °C–95 °C with an increment of 0.5 °C every 5 s. Ct values for target genes and internal controls in both control and experimental groups were recorded, and relative gene expression levels were calculated using the 2^−ΔΔCT^ method. Primer sequences are listed in Table 1.

**TABLE 1:** Primer sequences.

Target gene	Forward/Reverse (F/R)	Primer sequence	Product length/(bp)
β-actin	F	CTG​TGT​GGA​TTG​GTG​GCT​CT	136
	R	CAG​CTC​AGT​AAC​AGT​CCG​CC	
SREBP-1c	F	TAGTGTTGGCCTGCTTGG	106
	R	AGG​TCA​GCT​TGT​TTG​CGA​T	
ACC	F	ATG​GGC​GGA​ATG​GTC​TCT​TTC	148
	R	TGG​GGA​CCT​TGT​CTT​CAT​CAT	
FAS	F	CAA​CCA​TGC​CAA​CCT​GAA​AAC​TA	153
	R	ACC​CCC​TGC​AAT​TTC​CGT​T	
SCD1	F	AGC​TGC​TGG​AAG​AGT​ACC​TG	154
	R	GTG​GGC​AAA​GAC​GTT​GTA​GC	
ACOT1	F	CAG​CCA​CCC​CGA​GGT​AAA​AG	189
	R	CTC​AGG​ATA​GTC​ACA​GGG​GG	
ACOT4	F	AGC​AGT​GCG​GTA​CAT​GCT​TC	142
	R	AGA​GCC​ATT​GAT​GGA​AAC​TGT​G	
ACOT6	F	GCC​AGT​TCC​CTG​GGA​TCA​TC	151
	R	AGG​CGA​ACG​TCA​CTC​AGA​TTT	

### Statistics

Statistical analysis was performed using PRISM 9.5 software. Measurement data were expressed as mean ± standard deviation ( $\bar{x}\pm s$ ). For comparisons between two groups, the LSD test was applied for normally distributed data, whereas the nonparametric Mann–Whitney U test was used for non-normally distributed data. A *P* value <0.05 was considered statistically significant.

## Results

### Metabolites of WMSZY

**FIGURE 1:**
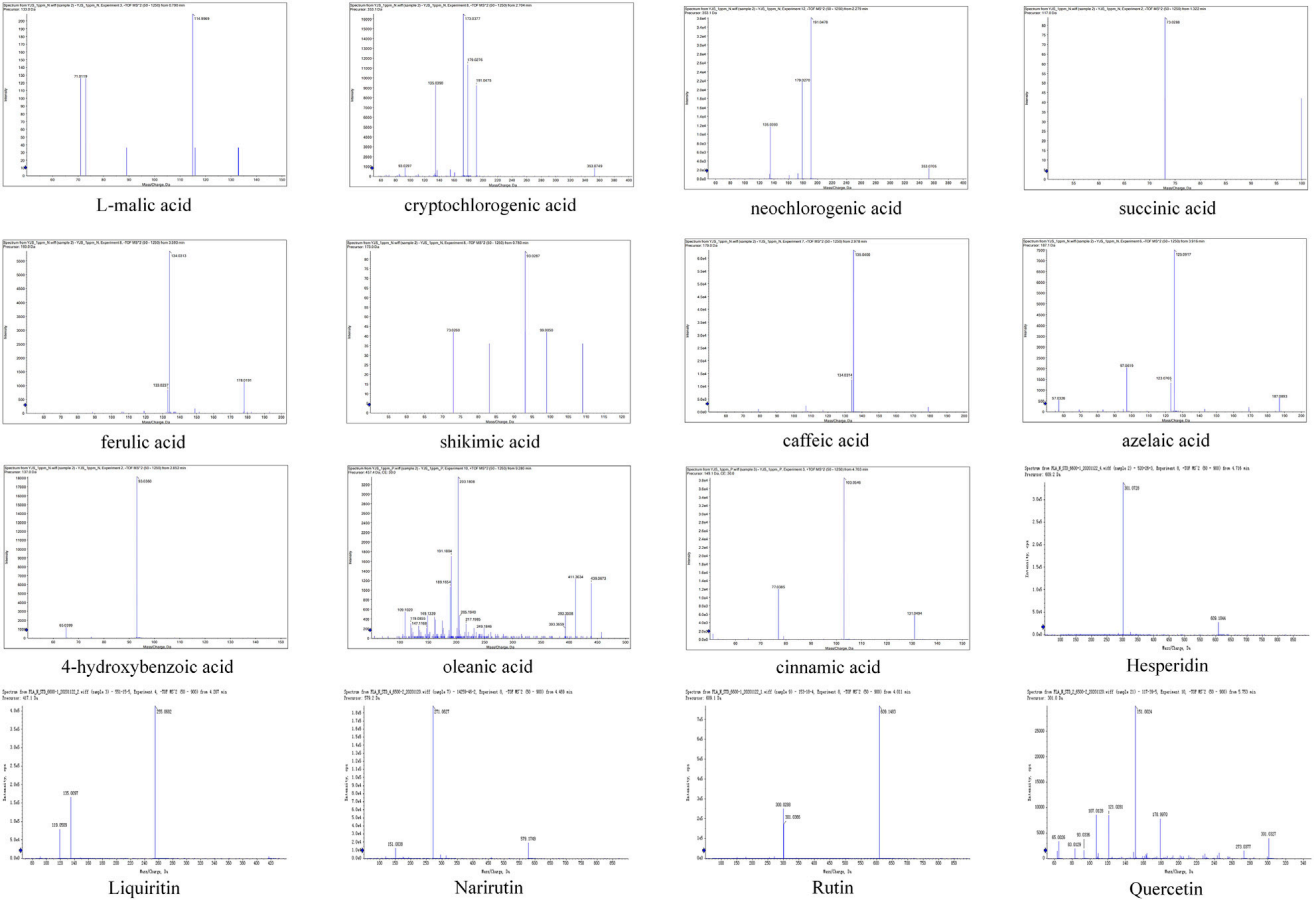
MS/MS spectra of the 16 metabolites identified in WMSZY using UPLC-MS/MS.

The metabolites of WMSZY was systematically characterized using UPLC-MS/MS. Mass spectrometry data were matched against the MWDB (Metware Database) constructed with authentic standards, and metabolite structures were confirmed based on retention time, MS/MS fragmentation patterns, and comparison with reference standards. A total of 16 metabolites were identified, including L-malic acid, cryptochlorogenic acid, neochlorogenic acid, succinic acid, ferulic acid, shikimic acid, caffeic acid, azelaic acid, 4-hydroxybenzoic acid, oleanic acid, cinnamic acid, hesperidin, liquiritin, narirutin, rutin, and quercetin. Detailed metabolite information and corresponding MS/MS spectra are presented in Figure 1 and Table 2.

**TABLE 2:** Detailed information on the 16 metabolites of WMSZY.

Compounds	Class	Ion mode	Ionization model	Content	Q1 (Da)	Q3 (Da)
L-malic acid	Organic Acid	Negative	[M−H]−	1,055,630 ng/mL	133	115.1
cryptochlorogenic acid	Organic Acid	Negative	[M−H]−	19,461.92 ng/mL	353.1	173.1
neochlorogenic acid	Organic Acid	Negative	[M−H]−	10,199.58 ng/mL	353.1	191.1
succinic acid	Organic Acid	Negative	[M−H]−	4,080.464 ng/mL	117	73
ferulic acid	Organic Acid	Negative	[M−H]−	2091.284 ng/mL	193	134
shikimic acid	Organic Acid	Negative	[M−H]−	1,204.966 ng/mL	172.8	93
caffeic acid	Organic Acid	Negative	[M−H]−	924.806 ng/mL	179	135.1
azelaic acid	Organic Acid	Negative	[M−H]−	687.642 ng/mL	187	125
4-hydroxybenzoic acid	Organic Acid	Negative	[M−H]−	408.630 ng/mL	137	93
oleanic acid	Organic Acid	Negative	[M−H]−	303.590 ng/mL	455.4	407.4
cinnamic acid	Organic Acid	Positive	[M + H]+	261.557 ng/mL	149.1	131.1
Hesperidin	Flavanones	Negative	[M−H]−	120.137 μmol/L	611.2	303.1
Liquiritin	Flavanones	Negative	[M−H]−	51.406 μmol/L	417.1	255.1
Narirutin	Flavanones	Negative	[M−H]−	43.659 μmol/L	579.2	271.1
Rutin	Flavonols	Negative	[M−H]−	2.196 μmol/L	579.2	271.1
Quercetin	Flavonols	Negative	[M−H]−	0.506 μmol/L	301.1	151

### WMSZY alleviates body weight gain and obesity in HFHF diet-induced mice

**FIGURE 2:**
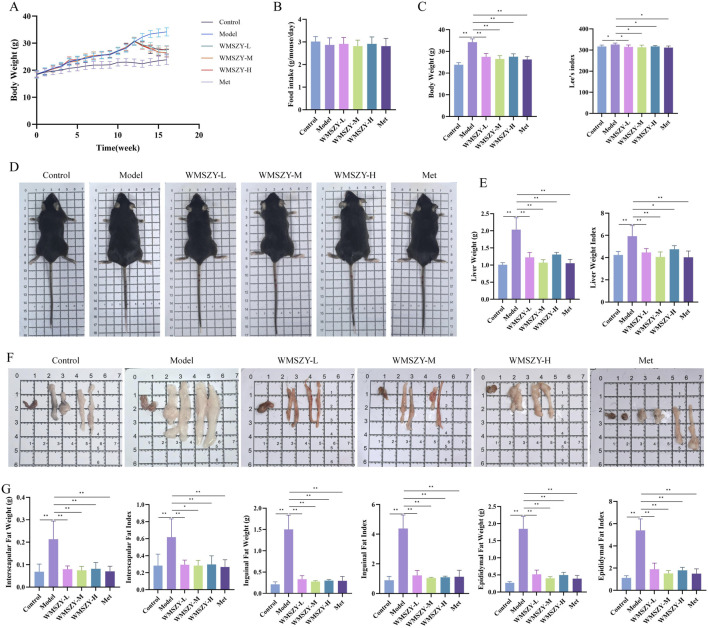
WMSZY Alleviates Body Weight Gain and Obesity in HFHF diet-induced mice (n = 6). **(A)** Body weight trend. **(B)** Daily food intake. **(C)** Effects of WMSZY on body weight and Lee’s index. **(D)** Mouse appearance. **(E)** Effects of WMSZY on liver weight and liver index. **(F)** Gross observation of epididymal, interscapular, and inguinal adipose tissues. **(G)** Effects of WMSZY on the weight and fat index of epididymal, interscapular, and inguinal adipose tissues. The results were expressed as mean ± SD. Compared with the Model group, ^*^ *p* < 0.05, ^**^ *p* < 0.01.

To investigate the anti-obesity effect of WMSZY, a HFHF diet-induced obese mouse model was established. Mice were fed an HFHF diet for 12 weeks, followed by oral administration of WMSZY (1.1, 2.2, or 4.4 g/kg/day) or metformin (0.1 g/kg/day) for 4 weeks. Experimental results showed that WMSZY markedly attenuated body weight gain and obesity-related parameters in HFHF diet-induced mice. Compared with the model group, all WMSZY and Met groups exhibited a slower trend of body weight gain (Figure 2A), with no significant difference in daily food intake (Figure 2B). In addition, WMSZY significantly reduced body weight and Lee’s index (Figures 2C,D) and visibly decreased fat accumulation, with a notable reduction in adipose tissue volume on gross observation (Figure 2F). Further analysis revealed significant reductions in liver weight and liver index in the WMSZY-treated groups (Figure 2E), along with decreased weights and fat indices of epididymal, interscapular, and inguinal adipose tissues (Figure 2G). These findings indicate that WMSZY effectively mitigates weight gain and obesity in HFHF diet-induced mice.

### WMSZY improves glucose and lipid metabolic disorders in HFHF diet-induced mice

**FIGURE 3:**
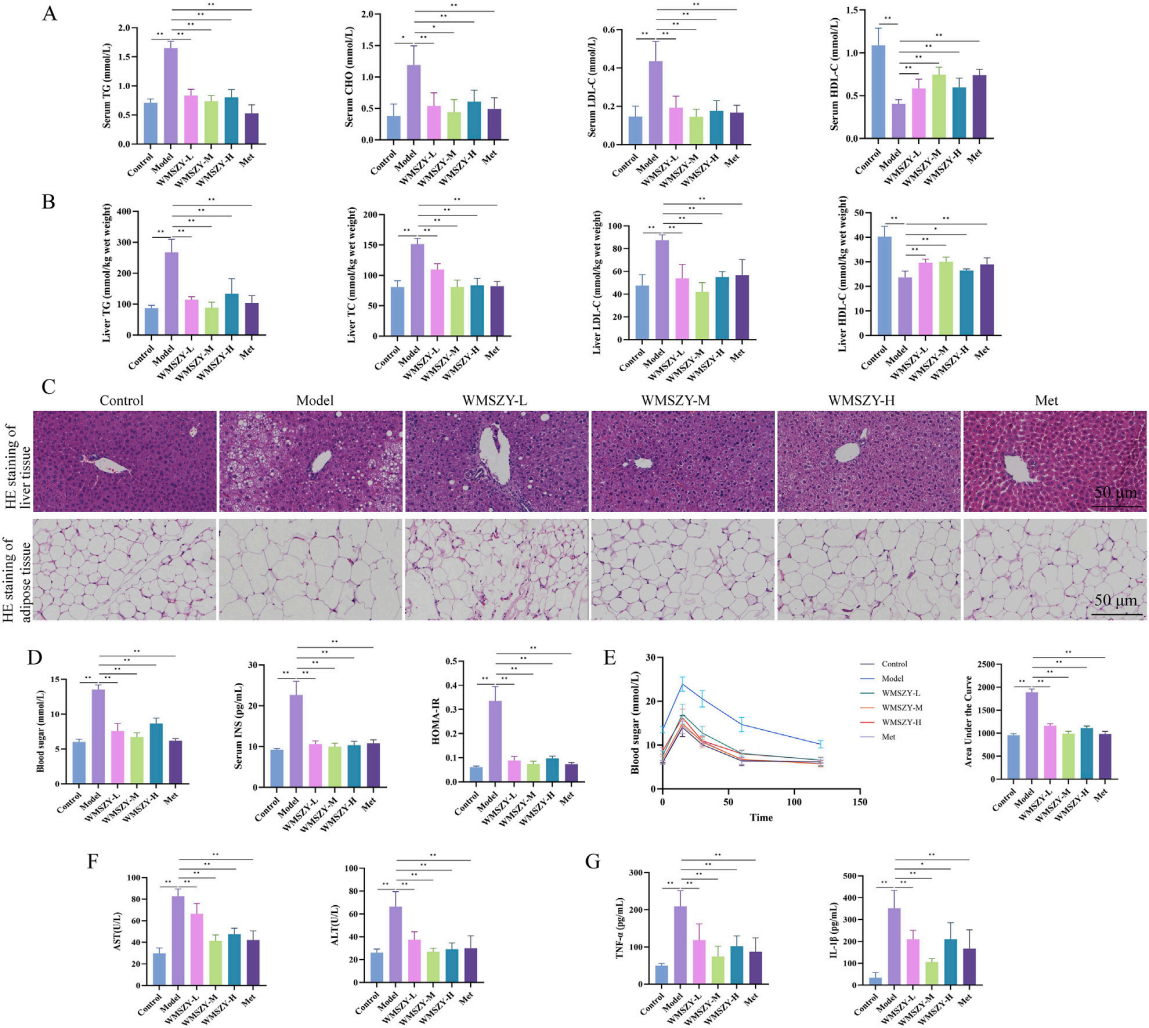
WMSZY improves glucose and lipid metabolic disorders in HFHF diet-induced mice (n = 6). **(A)** Effects of WMSZY on serum TC, TG, LDL-C, and HDL-C in HFHF diet-induced mice. **(B)** Effects of WMSZY on liver TC, TG, LDL-C, and HDL-C in HFHF diet-induced mice. **(C)** Effects of WMSZY on HE staining of liver and eWAT in HFHF diet-induced mice (scale bar: 50 μm; ×400). **(D)** Effects of WMSZY on blood sugar, insulin, and HOMA-IR in HFHF diet-induced mice. **(E)** Effects of WMSZY on OGTT in HFHF diet-induced mice. **(F)** Effects of WMSZY on serum AST and ALT in HFHF diet-induced mice. **(G)** Effects of WMSZY on TNF-α and IL-1β in HFHF diet-induced mice. The results were expressed as mean ± SD. Compared with the Model group, ^*^ *p* < 0.05, ^**^ *p* < 0.01.

Glucose and lipid metabolic disorders are key pathological features in the progression of obesity. In terms of lipid metabolism regulation, WMSZY significantly reduced serum and liver TC, TG, and LDL-C levels, while increasing HDL-C levels (Figures 3A,B). HE staining of liver and eWAT showed that the WMSZY-treated and Met groups exhibited a marked reduction in hepatic lipid vacuoles, more orderly cell arrangement, clearer lobular architecture, and smaller adipocyte size compared with the model group (Figure 3C). Regarding glucose metabolism, WMSZY significantly improved blood sugar, insulin levels, and HOMA-IR (Figure 3D), and OGTT results further confirmed its effect on enhancing insulin resistance (IR) (Figure 3E). The decrease in serum AST and ALT activity indicated a protective effect of WMSZY on liver function (Figure 3F). Inflammation assessment showed that WMSZY markedly suppressed TNF-α and IL-1β expression in eWAT (Figure 3G), both of which are closely associated with obesity-related chronic low-grade inflammation. Taken together, these changes in key metabolic indicators demonstrate that WMSZY effectively alleviates HFHF diet-induced obesity through multiple mechanisms, including the regulation of lipid metabolism, improvement of IR, mitigation of hepatic steatosis, and suppression of chronic inflammation.

### Lipidomics

**FIGURE 4:**
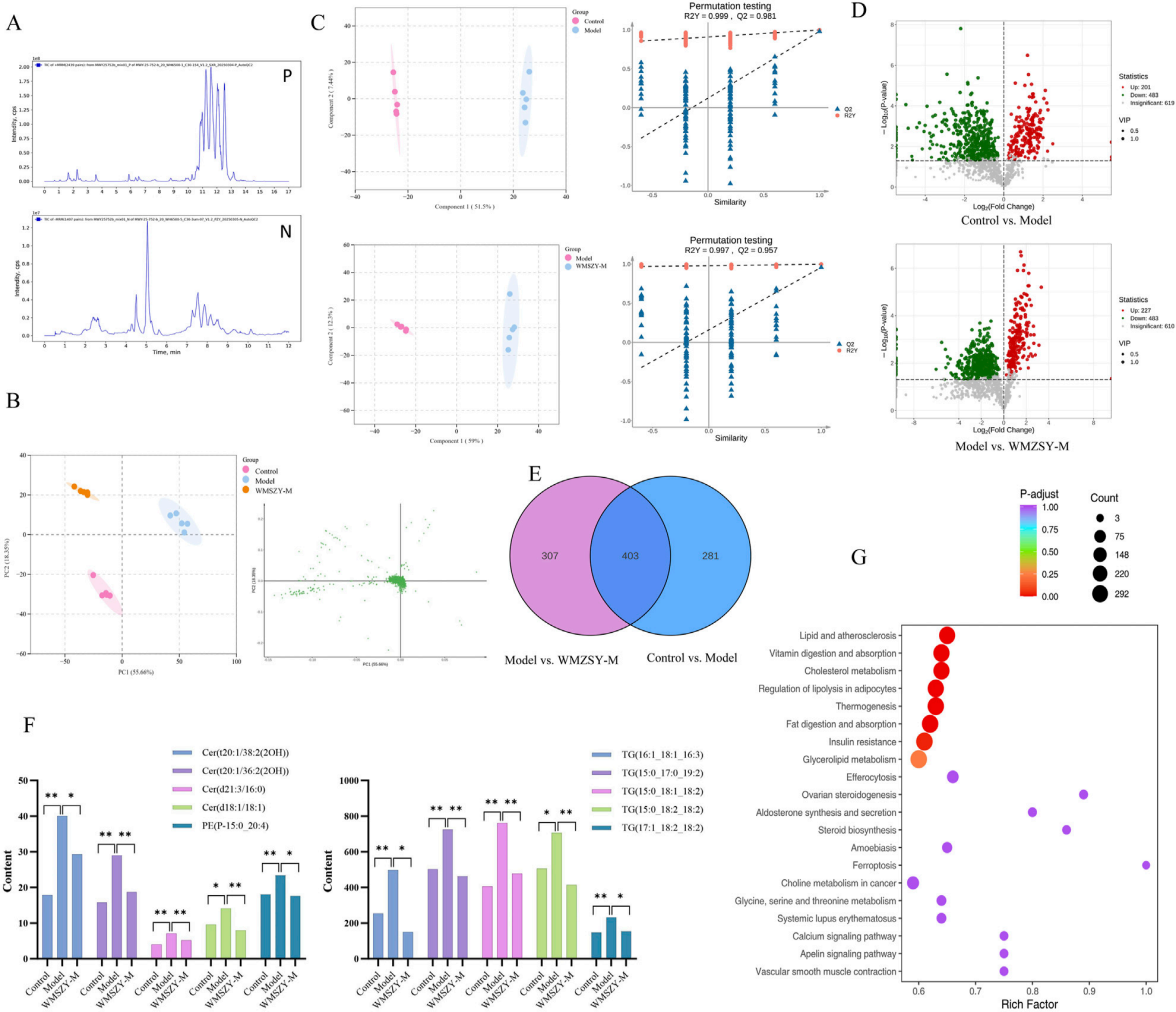
Lipidomics (n = 5). **(A)** TICs detected in positive and negative ion mode. **(B)** Scores scatter plot of lipidomic in Control, Model and WMZSY-M group as determined by PCA. **(C)** OPLS-DA plots of lipidomic in Control, Model and WMZSY-M group. **(D)** DALs volcano map of Control vs. Model and Model vs. WMZSY-M. **(E)** DALs venn diagram of Control vs. Model and Model vs. WMZSY-M. **(F)** The content changes of potential DALs after drug administration. **(G)** KEGG term pathway enriched by DALs (Model vs. WMZSY-M) in lipidomic. The results were expressed as mean ± SD. Compared with the Model group, ^*^ *p* < 0.05, ^**^ *p* < 0.01.

To further investigate the effects of WMSZY on adipose lipid metabolism, lipidomics analysis was performed of eWAT in mice. QC spectra in both positive and negative ion modes are shown Figure 4A. The PCA model delineated mice of distinct metabolic states and identified several lipids, including various ceramides and triglycerides, as the pivotal variables driving this separation (Figure 4B). OPLS-DA analysis was applied to distinguish differential metabolites. The R^2^Y and Q^2^ values for the control vs. model groups were 0.999 and 0.981, respectively, and for the model vs. WMSZY-M groups were 0.997 and 0.957, demonstrating the reliability of the OPLS-DA models. Permutation tests confirmed that the models were not overfitted and possessed strong explanatory and predictive power (Figure 4C). Using VIP >1 and *P <* 0.05 as the screening criteria, 684 differentially altered lipids (DALs) were identified between the control and model groups, including 201 upregulated and 483 downregulated species. Between the model and WMSZY-M groups, 710 DALs were identified, with 227 upregulated and 483 downregulated (Figure 4D). Venn diagram analysis revealed 403 common differential lipids among the three groups, suggesting that these molecules may participate in the core metabolic regulatory network involved in disease progression and drug intervention (Figure 4E). After WMSZY administration, changes were also observed in 10 potential differential lipid species, including Cer (Ceramides), PE (Phosphoethanolamines), and TG (Triacylglycerols), all of which were decreased compared with the model group (Figure 4F). KEGG pathway enrichment of DALs indicated that the anti-obesity mechanism of WMSZY may involve lipid and atherosclerosis pathways, cholesterol metabolism, regulation of adipocyte lipolysis, fat digestion and absorption, insulin resistance, and glycerolipid metabolism (Figure 4G).

### Transcriptomics

**FIGURE 5:**
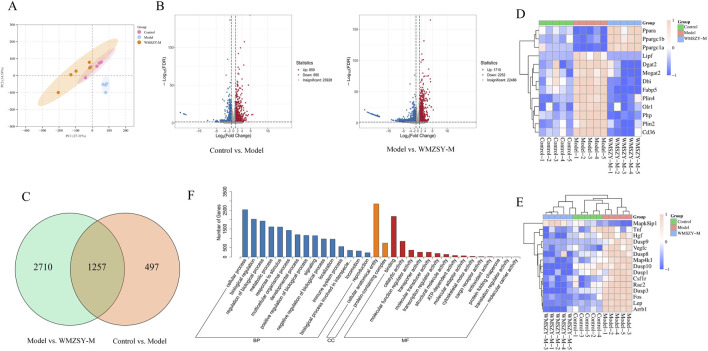
Transcriptomics (n = 5). **(A)** PCA of transcriptomes from the control, model, and WMSZY-M groups. **(B)** Volcano plots of DEGs; red indicates upregulated genes, blue indicates downregulated genes. **(C)** Venn diagram of DEGs between Control vs. Model and Model vs. WMSZY-M. **(D)** Heatmap of lipid metabolism-related DEGs. **(E)** Heatmap of inflammation-related DEGs. **(F)** GO enrichment analysis of DEGs in Model vs. WMSZY-M.

To further explore the pathways through which WMSZY ameliorates obesity, transcriptomics of eWAT in mice was performed. The results revealed that WMSZY-M significantly modulated the gene expression profile of eWAT in HFHF diet-induced obese mice. PCA demonstrated clear clustering of the control, model, and WMSZY-M groups, indicating that WMSZY intervention markedly altered the transcriptional pattern of eWAT in mice (Figure 5A). Volcano plot analysis, based on |FC| ≥ 2, *P* < 0.05, and FDR <0.05 as the criteria for differential expression, showed that 1,754 DEGs were identified between the control and model groups, including 859 upregulated and 895 downregulated genes. Comparison of the model and WMSZY-M groups yielded 3,967 DEGs, with 1,715 upregulated and 2,252 downregulated (Figure 5B). Venn diagram analysis revealed 1,257 overlapping DEGs among the three groups (Figure 5C). Heatmap analysis further illustrated the expression patterns of DEGs, with genes related to lipid metabolism and inflammation showing pronounced changes (Figures 5D,E). Gene Ontology (GO) enrichment analysis indicated that these DEGs were mainly enriched in biological processes such as “regulation of biological process” and “metabolic process”, suggesting that WMSZY may exert its anti-obesity and metabolic regulatory effects through these biological pathways (Figure 5F).

### WMSZY may ameliorate obesity *via* the AMPK/CPT1 signaling pathway

**FIGURE 6:**
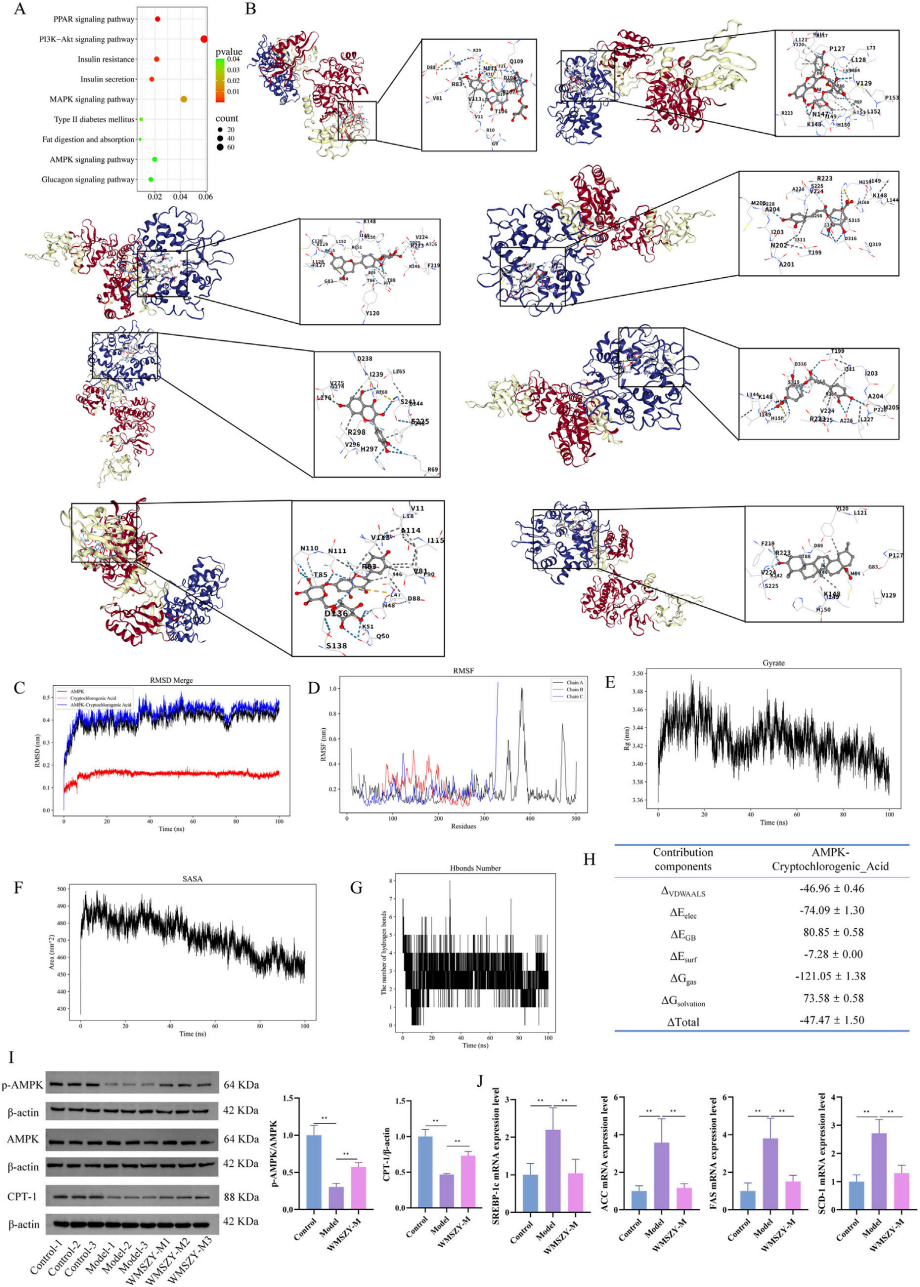
WMSZY may ameliorate obesity *via* the AMPK/CPT1 signaling pathway. **(A)** KEGG enrichment of DEGs in Model vs. WMSZY-M. **(B)** Molecular docking simulation of WMSZY metabolites with AMPK (from top left to bottom right: hesperidin −8.8, rutin −8.8, liquiritin −9.3, neochlorogenic acid −7.5, quercetin −8.1, cryptochlorogenic acid −7.5, narirutin −9.4, oleanolic acid −9.0). **(C)** RMSD of the protein–ligand complex. **(D)** RMSF of the complex. **(E)** Gyrate of the complex. **(F)** SASA of the complex. **(G)** Hydrogen bond analysis. **(H)** Energy decomposition of the complex. **(I)** Effects of WMSZY on AMPK and CPT-1 protein expression in eWAT of HFHF diet-fed mice (n = 5). **(J)** Effects of WMSZY on SREBP-1c, ACC, FAS, and SCD1 mRNA expression in eWAT of HFHF diet-fed mice (n = 5). The results were expressed as mean ± SD. Compared with the Model group, *p < 0.05, **p < 0.01.

KEGG pathway enrichment analysis of the transcriptome indicated that the anti-obesity effect of WMSZY may be associated with signaling pathways such as AMPK, MAPK, and PPAR (Figure 6A). Molecular docking simulation analysis revealed that key metabolites of WMSZY—including hesperidin, liquiritin, quercetin, rutin, neochlorogenic acid, cryptochlorogenic acid, narirutin, and oleanolic acid—showed strong binding affinities to AMPK, with docking scores all below −7 kcal/mol (Figure 6B). Further molecular dynamics simulation of cryptochlorogenic acid, a major metabolite in WMSZY, with AMPK demonstrated the formation of a stable protein-ligand complex, which reached equilibrium after 20 ns. The overall structure exhibited good stability and rigidity (RMSD 0.43 ± 0.04 nm; RMSF 0.19 ± 0.12 nm) (Figures 6C,D). The radius of gyration remained stable at 3.43 ± 0.02 nm, and the solvent-accessible surface area decreased from 490 nm^2^ to 460 nm^2^, indicating a progressively compact complex conformation (Figures 6E,F). The protein–ligand complex maintained one to eight hydrogen bonds (average 3) (Figure 6G), and binding free energy analysis revealed that van der Waals interactions (−46.96 ± 0.46 kcal/mol) and electrostatic interactions (−74.09 ± 1.30 kcal/mol) were the major driving forces, with a total binding free energy of −47.47 ± 1.50 kcal/mol, suggesting strong binding affinity (Figure 6H). Western blot analysis showed that WMSZY markedly increased AMPK phosphorylation in eWAT and enhanced the expression of CPT-1, a key regulator of fatty acid β-oxidation (Figure 6I). RT-qPCR results further demonstrated that WMSZY effectively suppressed the mRNA expression of lipid metabolism related genes, including SREBP-1c, ACC, FAS, and SCD1 (Figure 6J). Collectively, these results indicate that WMSZY alleviates lipid accumulation by activating the AMPK/CPT1 signaling pathway, suppressing *de novo* lipogenesis, and promoting fatty acid oxidation.

### Integrated lipidomics and transcriptomics analysis

**FIGURE 7:**
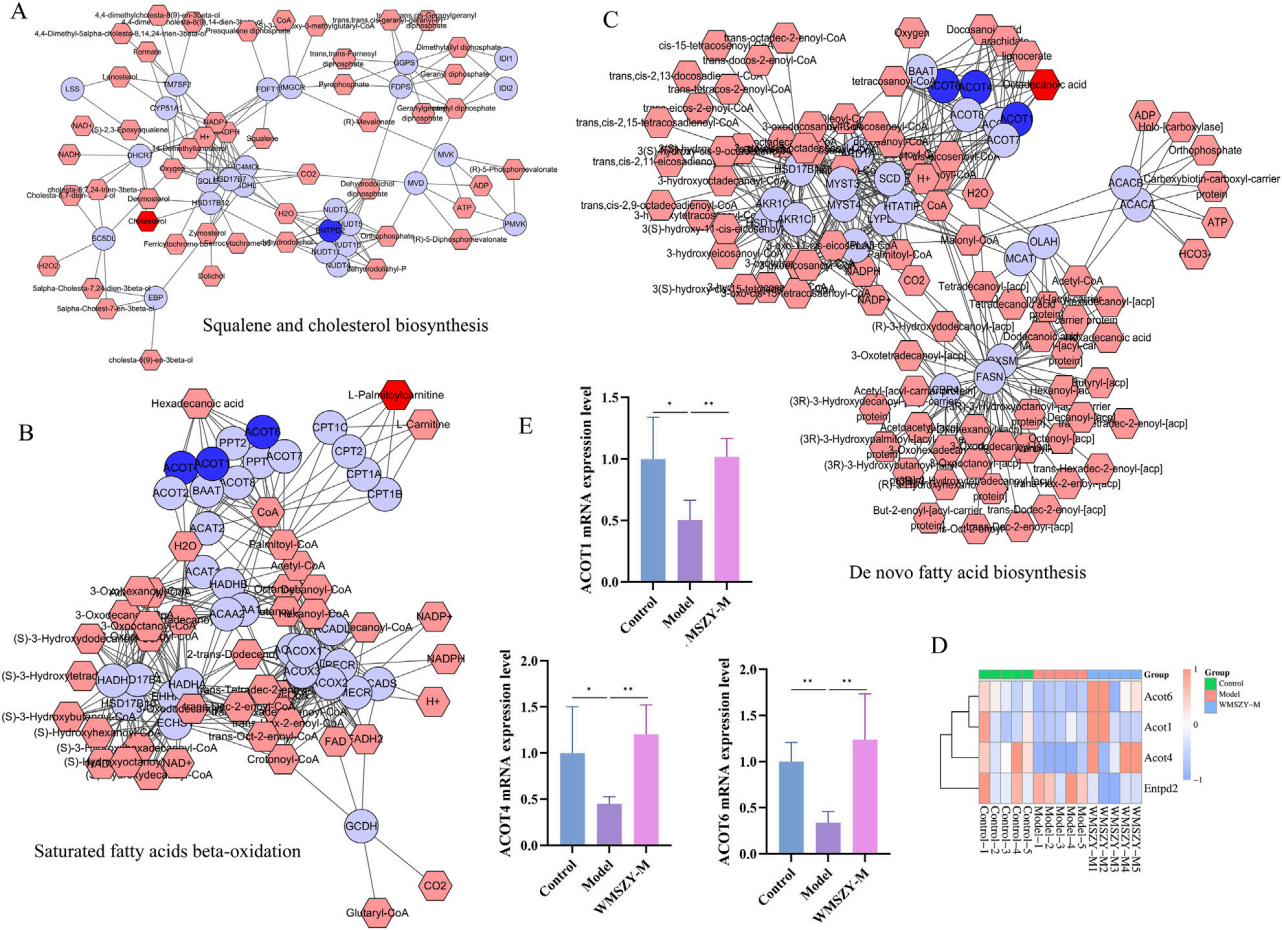
Integrated Lipidomics and Transcriptomics Analysis. **(A–C)** Compound-reaction-enzyme-gene networks for key metabolites and genes. Red hexagons represent active metabolites; blue circles represent key genes. **(D)** Heatmap for key genes. **(E)** Detection of ACOT1, ACOT4 and ACOT6 mRNA expression. n = 5. The results were expressed as mean ± SD. Compared with the Model group, *p < 0.05, **p < 0.01.

To investigate the relationship between DALs and DEGs, Metscape software was employed for further analysis. After matching the differential genes and metabolites between the model and WMSZY-M groups, three key pathways were identified: squalene and cholesterol biosynthesis, *de novo* fatty acid biosynthesis, and saturated fatty acid β-oxidation. These pathways involved four key target genes—ENTPD2, ACOT4, ACOT6, and ACOT1 (blue hexagons in Figures 7A–C)—and three key lipid metabolites—octadecanoic acid, cholesterol, and L-palmitoylcarnitine (red hexagons in Figures 7A–C). These potential targets suggest that the anti-obesity effects of WMSZY may be associated with metabolic regulation, lipid signaling, and energy homeostasis. Subsequently, a heatmap of the key genes (Figure 7D) was generated, and RT-qPCR validation confirmed that WMSZY-M treatment reversed the expression of ACOT4, ACOT6, and ACOT1 (Figure 7E). These findings were consistent with the transcriptomic results.

## Discussion

In this study, UPLC-MS/MS was employed to quantitatively identify metabolites of WMSZY, including L-malic acid, cryptochlorogenic acid, neochlorogenic acid, succinic acid, ferulic acid, shikimic acid, caffeic acid, azelaic acid, 4-hydroxybenzoic acid, oleanolic acid, cinnamic acid, hesperidin, liquiritin, narirutin, rutin, and quercetin (Figure 1). Among these, cryptochlorogenic acid (Xu et al., 2024), shikimic acid (Kim et al., 2019), rutin (Liu et al., 2024), hesperidin (Xiong et al., 2019), quercetin (Wang Y. et al., 2024) and oleanolic acid (Zhang G. et al., 2024) have been reported to regulate lipid metabolic disorders, reduce fat accumulation, and alleviate inflammation and oxidative stress, possibly through modulation of AMPK and its downstream pathways. Neochlorogenic acid has been shown to improve lipid profiles, hepatic steatosis, and inflammation in db/db mice with glucotoxicity, possibly by downregulating miR-34a and activating the SIRT1/AMPK pathway (Tsai et al., 2024; Yu et al., 2021). Succinic acid may promote browning and upregulation of mitochondria-associated genes in inguinal white adipose tissue (iWAT) by activating the PI3K-AKT/MAPK signaling pathway, thereby suppressing adipocyte hypertrophy induced by high-fat diet (Yang et al., 2024). Ferulic acid has been reported to protect the liver from iron overload-induced disturbances in lipid and bile acid metabolism by targeting FASN and CYP7A1 (Liang et al., 2024). Caffeic acid (Zhang J. et al., 2024) and azelaic acid (Muthulakshmi and Saravanan, 2013) have been shown to enhance fatty acid utilization and reduce hepatic lipid accumulation, lipotoxicity, and oxidative damage. Cinnamic acid may target PPARδ and attenuate endothelial dysfunction and oxidative stress in diabetes and obesity through activation of the Nrf2/HO-1 and Akt/eNOS signaling pathways (Bai et al., 2025).

The fundamental cause of obesity lies in an imbalance between energy intake and expenditure. Excessive accumulation of body fat can lead to a range of metabolic abnormalities and diseases, including insulin resistance, dyslipidemia, non-alcoholic fatty liver disease (NAFLD), prediabetes, and type 2 diabetes (Blüher, 2019). In this study, WMSZY was shown to significantly reduce body weight, Lee’s index, liver weight, fat depot weights, liver index, and fat indices in HFHF diet-induced mice (Figure 2). These findings were consistent with histological observations of eWAT, which revealed a reduction in adipocyte size. The liver, as a central metabolic organ, is highly susceptible to obesity-induced dysregulation of lipid metabolism. IR in adipose tissue impairs the suppression of lipolysis, resulting in elevated fatty acid flux to the liver. This promotes hepatic gluconeogenesis and increases triglyceride synthesis, contributing to the development of NAFLD and hyperlipidemia (Klein et al., 2022). The present results demonstrated that WMSZY effectively regulated the dyslipidemia and liver lipid metabolism (TC, TG, LDL-C, and HDL-C) in obese mouse models, and alleviated lipid deposition in liver and eWAT(Figures 3A–C). Cer(t20:1/38:2(2OH)), Cer(t20:1/36:2(2OH)), Cer(d21:3/16:0), and Cer(d18:1/18:1) are ceramides, which are core molecules of sphingolipids; targeted inhibition of their *de novo* synthesis in the liver has been shown to improve lipid uptake and lipogenesis (Yu et al., 2025). In HFD-induced obese mice, TG and PE levels were elevated (Pang et al., 2022). These are consistent with the lipidomics results of the present study, and WMSZY was able to reverse this outcome (Figure 4F).

In adipose tissue, various factors—such as adipocyte hypoxia, increased infiltration of pro-inflammatory immune cells (e.g., macrophages and T cells), elevated levels of inflammatory cytokines, enhanced lipolysis and free fatty acid (FFA) release into circulation, and increased secretion of adipose-derived exosomes—can contribute to systemic IR (Klein et al., 2022). Additionally, obesity is characterized by chronic low-grade inflammation. Adipose tissue inflammation involves increased reactive oxygen species (ROS) levels and altered secretion of adipokines and cytokines, including elevated levels of leptin, resistin, IL-6, and MCP-1, along with decreased adiponectin levels (Soták et al., 2025). Chronic metabolic stress leads to sustained activation of the NLRP3 inflammasome, resulting in IL-1β release, which further promotes IR (de Baat et al., 2023). Inflammation also enhances the activation of M1-like macrophages, which secrete TNF and impair insulin signaling in adipocytes (Hägglöf et al., 2022). This persistent crosstalk between chronic inflammation and insulin resistance drives the progression of metabolic diseases. The present study showed that WMSZY significantly improved blood sugar, insulin levels, and HOMA-IR index in HFHF diet-induced mice, indicating amelioration of IR. Moreover, WMSZY markedly reduced serum AST and ALT levels, suppressed the expression of TNF-α and IL-1β in eWAT, and alleviated local inflammatory responses (Figures 3F,G).

AMPK is a metabolic energy sensor that regulates lipid and glucose metabolism. It is activated by an increased intracellular AMP/ATP ratio and exerts broad effects on cellular metabolism (Canbolat and Cakıroglu, 2023). Excessive fat intake leads to the accumulation of free fatty acids, malonyl-CoA, and diacylglycerol (DAG) in tissues, which activates protein kinase C (PKC), and this activation inhibits AMPK by phosphorylating serine residues at positions 485/491 and also impairs insulin signaling by inhibiting insulin receptor substrates, contributing to insulin resistance (Coughlan et al., 2013). AMPK activation reduces macrophage infiltration and inflammation in adipose tissue and enhances SIRT1 activity by increasing the NAD^+^/NADH ratio, thereby preventing diet-induced inflammation and obesity-related metabolic dysfunction (Xu et al., 2021). In general, AMPK activation suppresses anabolic processes and promotes catabolic pathways (Canbolat and Cakıroglu, 2023). AMPK activation downregulates SREBP-1c expression and subsequently inhibits the expression of lipogenic genes such as ACC, FAS, SCD1, and HMGCR in insulin-resistant mice (Li et al., 2011). CPT-1 regulates fatty acid β-oxidation by catalyzing the conversion of acyl-CoA to acylcarnitine in the mitochondria. AMPK activation upregulates CPT-1 expression, thereby promoting fatty acid oxidation and lipolysis while limiting fatty acid uptake (Tang et al., 2020). In the present study, WMSZY was found to enhance AMPK phosphorylation, suppress the expression of genes related to fatty acid and triglyceride synthesis (SREBP-1c, ACC, FAS, and SCD1), and upregulate CPT-1 expression, collectively alleviating fat accumulation in HFHF diet-induced mice (Figures 6I,J).

To further investigate the relationship between DEGs and DALs, three differential lipid species (octadecanoic acid, cholesterol, and L-palmitoylcarnitine) and four potential targets (ACOT4, ACOT1, ACOT6, and ENTPD2) were identified. Octadecanoic acid can be obtained from the diet, synthesized *de novo via* the ACC–FAS complex, or produced by the elongation of palmitic acid through fatty acid elongases, and may subsequently be incorporated into complex lipids or desaturated into oleic acid by SCD1(Sampath and Ntambi, 2005). In the context of obesity, cholesterol synthesis is elevated while clearance and reverse cholesterol transport are impaired, leading to its abnormal accumulation in adipose tissue and circulation (Duan et al., 2022). A high-fat diet provides abundant triglycerides and fatty acids, wherein long-chain fatty acids are converted into carnitine derivatives such as L-palmitoylcarnitine, which are transported from the cytoplasm into mitochondria at the onset of fatty acid oxidation, where they undergo β-oxidation to generate medium-chain fatty acids such as capric and lauric acids, ultimately yielding acetyl-CoA (Hu et al., 2017). ACOT1, ACOT4, and ACOT6 are acyl-CoA thioesterases that catalyze the hydrolysis of acyl-CoA into FFAs and CoASH, playing key roles in fatty acid metabolism, and their regulation may help alleviate lipid metabolic disorders (Hunt et al., 2012; Pashaj et al., 2013; Sun et al., 2023; Wang et al., 2022; Zhong et al., 2023). The current study showed that WMSZY upregulated the expression of ACOT1, ACOT4, and ACOT6, thereby contributing to the regulation of fatty acid metabolism (Figure 7). ENTPD2 is a membrane-bound ectonucleotidase belonging to the ENTPDase family and functions mainly to hydrolyze extracellular ATP and ADP. It participates in a variety of physiological and pathological processes through the regulation of purinergic signaling (Chiu et al., 2017). Although the involvement of ENTPD2 in obesity or metabolic dysregulation remains poorly characterized, the integrative transcriptomic and lipidomic analysis of eWAT identified ENTPD2 as a key DEG potentially involved in adipose tissue regulation. This finding provides a novel perspective for exploring the molecular mechanisms of ENTPD2 in maintaining adipose tissue homeostasis and in the pathogenesis of metabolic diseases.

The metabolites of WMSZY identified in this study are primarily natural polyphenols and organic acids. Although we endeavored to screen for metabolites with high binding energy, it must be noted that such small-molecule polyphenols are prone to non-specific “docking” effects in computational simulations, and the prediction results warrant caution regarding potential pan-assay interference compounds (PAINS) risks. Consequently, the current docking results should be regarded as preliminary hypotheses regarding the mechanism of action. The true binding affinity of these metabolites and their specific pharmacological mechanisms must be rigorously validated through future experimental studies, such as surface plasmon resonance (SPR), isothermal titration calorimetry (ITC), and functional cellular reporter assays. Furthermore, the bioavailability, *in vivo* metabolic processes, and potential synergistic effects of the metabolites in WMSZY remain unclear, representing core issues to be addressed in future pharmacokinetic research. Finally, to ensure the reproducibility of our findings and the consistency of future preparations, establishing a more comprehensive quality control system based on fingerprinting is crucial.

## Conclusion

This study identified multiple active metabolites in WMSZY using UPLC-MS/MS, including organic acids (e.g., cryptochlorogenic acid, shikimic acid, succinic acid) and flavonoids (e.g., rutin, hesperidin). Experimental results demonstrated that WMSZY effectively reduced body weight, adipose tissue weight, and liver lipid accumulation, modulated lipid profiles (TC, TG, LDL-C, HDL-C), and improved IR and chronic low-grade inflammation. Mechanistically, this study employed an integrated transcriptomics and targeted lipidomics strategy to elucidate the anti-obesity mechanisms of WMSZY. This approach not only enhanced the reliability of the findings through mutual validation of multi-dimensional data (Migni et al., 2025), but also precisely captured the systemic regulatory effects of WMSZY at the levels of gene expression and lipid metabolic networks. These results suggest that WMSZY may ameliorate obesity through the AMPK/CPT-1 pathway. This strategy aligns with the current trend of utilizing advanced multi-omics technologies to screen and validate the pharmacological activities of natural products, providing robust evidence for comprehensively revealing the mechanisms of action of complex natural extracts.

## Data Availability

Raw data relevant to the conclusions of this study will be provided by the corresponding authors upon reasonable request.
