# Supplementary material for: Anti-obesity effects of Wumeishanzhayin: an integrated lipidomics and transcriptomics study
Source: Front Pharmacol. 2025 Oct 21;16:1697683. doi: 10.3389/fphar.2025.1697683 (PMC12584145; doi:10.3389/fphar.2025.1697683)
Supplement: Supplementary file 1 [file Supplementaryfile1.docx]

Supplementary Table 1

List of Quantified Lipids

| Index | Compounds | Class I | Class II | Q1 (Da) | Molecular Weight | Ionization model | Formula |
| --- | --- | --- | --- | --- | --- | --- | --- |
| LIPID-N-0001 | taurolithocholicacid-3-sulfate | ST | BA | 281.10 | 563.26 | [M-H]- | C26H45NO8S2 |
| LIPID-N-0005 | Chenodeoxycholic acid | ST | BA | 391.50 | 392.29 | [M-H]- | C24H40O4 |
| LIPID-N-0007 | norcholic acid | ST | BA | 393.20 | 394.27 | [M-H]- | C24H38O3 |
| LIPID-N-0010 | Ursocholic acid | ST | BA | 407.40 | 408.29 | [M-H]- | C24H40O5 |
| LIPID-N-0016 | Taurochenodeoxycholic acid | ST | BA | 498.50 | 499.30 | [M-H]- | C26H45NO6S |
| LIPID-P-1578 | BMP(22:6_22:6) | GP | BMP | 884.54 | 866.51 | [M+NH4]+ | C50H75O10P |
| LIPID-P-0036 | Carnitine C12:1-OH | FA | CAR | 358.26 | 357.25 | [M+H]+ | C19H35NO5 |
| LIPID-P-0037 | Carnitine C14:1 | FA | CAR | 370.30 | 369.29 | [M+H]+ | C21H39NO4 |
| LIPID-P-0038 | Carnitine C14:1-OH | FA | CAR | 386.29 | 385.28 | [M+H]+ | C21H39NO5 |
| LIPID-P-0039 | Carnitine C16:1 | FA | CAR | 398.33 | 397.32 | [M+H]+ | C23H43NO4 |
| LIPID-P-0040 | Carnitine C16:1-OH | FA | CAR | 414.32 | 413.31 | [M+H]+ | C23H43NO5 |
| LIPID-P-0042 | Carnitine C18:1 | FA | CAR | 426.36 | 425.35 | [M+H]+ | C25H47NO4 |
| LIPID-P-0044 | Carnitine C22:1 | FA | CAR | 482.42 | 481.41 | [M+H]+ | C29H55NO4 |
| LIPID-P-0028 | Carnitine C5:1 | FA | CAR | 244.16 | 243.15 | [M+H]+ | C12H21NO4 |
| LIPID-P-0049 | Carnitine C18:2 | FA | CAR | 424.34 | 423.33 | [M+H]+ | C25H45NO4 |
| LIPID-P-0051 | Carnitine C18:2-OH | FA | CAR | 440.34 | 439.33 | [M+H]+ | C25H45NO5 |
| LIPID-P-1300 | Carnitine C20:3 | FA | CAR | 450.36 | 449.35 | [M+H]+ | C27H47NO4 |
| LIPID-P-1302 | Carnitine C20:4 | FA | CAR | 448.34 | 447.33 | [M+H]+ | C27H45NO4 |
| LIPID-P-1307 | Carnitine C22:6 | FA | CAR | 472.34 | 471.33 | [M+H]+ | C29H45NO4 |
| LIPID-P-0045 | Carnitine C18:1-OH | FA | CAR | 442.35 | 441.35 | [M+H]+ | C25H47NO5 |
| LIPID-P-0027 | Carnitine C4:1-2OH | FA | CAR | 262.13 | 261.12 | [M+H]+ | C11H19NO6 |
| LIPID-P-0024 | Carnitine C20:0 | FA | CAR | 456.40 | 455.40 | [M+H]+ | C27H53NO4 |
| LIPID-P-0025 | Carnitine C18-OH | FA | CAR | 444.37 | 443.36 | [M+H]+ | C25H49NO5 |
| LIPID-P-0003 | Carnitine C3:0 | FA | CAR | 218.14 | 217.13 | [M+H]+ | C10H19NO4 |
| LIPID-P-0004 | Carnitine C4:0 | FA | CAR | 232.16 | 231.15 | [M+H]+ | C11H21NO4 |
| LIPID-P-0006 | Carnitine C4-OH | FA | CAR | 248.15 | 247.14 | [M+H]+ | C11H21NO5 |
| LIPID-P-0007 | Carnitine C6:0 | FA | CAR | 260.19 | 259.18 | [M+H]+ | C13H25NO4 |
| LIPID-P-0008 | Carnitine C5-OH | FA | CAR | 262.17 | 261.16 | [M+H]+ | C12H23NO5 |
| LIPID-P-0009 | Carnitine C6-OH | FA | CAR | 276.18 | 275.17 | [M+H]+ | C13H25NO5 |
| LIPID-P-0010 | Carnitine C8:0 | FA | CAR | 288.22 | 287.21 | [M+H]+ | C15H29NO4 |
| LIPID-P-0011 | Carnitine C6-2OH | FA | CAR | 290.16 | 289.15 | [M+H]+ | C13H23NO6 |
| LIPID-P-0013 | Carnitine C10:0 | FA | CAR | 316.25 | 315.24 | [M+H]+ | C17H33NO4 |
| LIPID-P-0016 | Carnitine C12-OH | FA | CAR | 360.28 | 359.27 | [M+H]+ | C19H37NO5 |
| LIPID-P-0017 | Carnitine C14:0 | FA | CAR | 372.31 | 371.30 | [M+H]+ | C21H41NO4 |
| LIPID-P-0021 | Carnitine C16:0 | FA | CAR | 400.34 | 399.33 | [M+H]+ | C23H45NO4 |
| LIPID-P-0022 | Carnitine C16-OH | FA | CAR | 416.34 | 415.33 | [M+H]+ | C23H45NO5 |
| LIPID-P-0023 | Carnitine C18:0 | FA | CAR | 428.38 | 427.37 | [M+H]+ | C25H49NO4 |
| LIPID-P-0026 | Carnitine C3:1-2OH | FA | CAR | 248.11 | 247.11 | [M+H]+ | C10H17NO6 |
| LIPID-P-0002 | Carnitine C2:0 | FA | CAR | 204.12 | 203.12 | [M+H]+ | C9H17NO4 |
| LIPID-P-0001 | DL-Carnitine | FA | CAR | 162.11 | 161.11 | [M+H]+ | C7H15NO3 |
| LIPID-P-0073 | CE(18:2) | ST | CE | 666.63 | 648.58 | [M+NH4]+ | C45H76O2 |
| LIPID-P-0056 | CE(16:0) | ST | CE | 642.63 | 624.58 | [M+NH4]+ | C43H76O2 |
| LIPID-P-0057 | CE(18:0) | ST | CE | 670.66 | 652.62 | [M+NH4]+ | C45H80O2 |
| LIPID-P-0059 | CE(20:0) | ST | CE | 698.69 | 680.65 | [M+NH4]+ | C47H84O2 |
| LIPID-P-0066 | CE(18:1) | ST | CE | 668.64 | 650.60 | [M+NH4]+ | C45H78O2 |
| LIPID-P-0084 | CE(22:4) | ST | CE | 718.66 | 700.62 | [M+NH4]+ | C49H80O2 |
| LIPID-P-0068 | CE(20:1) | ST | CE | 696.68 | 678.63 | [M+NH4]+ | C47H82O2 |
| LIPID-P-0070 | CE(24:1) | ST | CE | 752.73 | 734.69 | [M+NH4]+ | C51H90O2 |
| LIPID-P-0069 | CE(22:1) | ST | CE | 724.71 | 706.66 | [M+NH4]+ | C49H86O2 |
| LIPID-P-1503 | Cer(d18:0/22:1(2OH)) | SP | Cer-ADS | 638.61 | 637.60 | [M+H]+ | C40H79NO4 |
| LIPID-P-1967 | Cer(t20:1/40:2(2OH)) | SP | Cer-AP | 912.87 | 929.88 | [M-H2O+H]+ | C60H115NO5 |
| LIPID-P-1966 | Cer(t20:1/38:2(2OH)) | SP | Cer-AP | 884.84 | 901.85 | [M-H2O+H]+ | C58H111NO5 |
| LIPID-P-1965 | Cer(t20:1/36:2(2OH)) | SP | Cer-AP | 856.81 | 873.81 | [M-H2O+H]+ | C56H107NO5 |
| LIPID-P-1989 | Cer(t24:1/34:2(2OH)) | SP | Cer-AP | 884.84 | 901.85 | [M-H2O+H]+ | C58H111NO5 |
| LIPID-P-1962 | Cer(t18:2/42:2(2OH)) | SP | Cer-AP | 910.86 | 927.86 | [M-H2O+H]+ | C60H113NO5 |
| LIPID-P-1961 | Cer(t18:2/40:2(2OH)) | SP | Cer-AP | 882.83 | 899.83 | [M-H2O+H]+ | C58H109NO5 |
| LIPID-P-1960 | Cer(t18:2/38:2(2OH)) | SP | Cer-AP | 854.80 | 871.80 | [M-H2O+H]+ | C56H105NO5 |
| LIPID-P-1958 | Cer(t18:2/36:2(2OH)) | SP | Cer-AP | 826.76 | 843.77 | [M-H2O+H]+ | C54H101NO5 |
| LIPID-P-1957 | Cer(t18:1/40:2(2OH)) | SP | Cer-AP | 884.84 | 901.85 | [M-H2O+H]+ | C58H111NO5 |
| LIPID-P-1956 | Cer(t18:1/38:2(2OH)) | SP | Cer-AP | 856.81 | 873.81 | [M-H2O+H]+ | C56H107NO5 |
| LIPID-P-1955 | Cer(t18:1/38:1(2OH)) | SP | Cer-AP | 858.83 | 875.83 | [M-H2O+H]+ | C56H109NO5 |
| LIPID-P-1968 | Cer(t20:2/18:0(2OH)) | SP | Cer-AP | 606.55 | 623.55 | [M-H2O+H]+ | C38H73NO5 |
| LIPID-P-1970 | Cer(t20:2/32:1(2OH)) | SP | Cer-AP | 800.75 | 817.75 | [M-H2O+H]+ | C52H99NO5 |
| LIPID-P-1971 | Cer(t20:2/34:1(2OH)) | SP | Cer-AP | 828.78 | 845.78 | [M-H2O+H]+ | C54H103NO5 |
| LIPID-P-1972 | Cer(t20:2/34:2(2OH)) | SP | Cer-AP | 826.76 | 843.77 | [M-H2O+H]+ | C54H101NO5 |
| LIPID-P-1982 | Cer(t22:1/38:2(2OH)) | SP | Cer-AP | 912.87 | 929.88 | [M-H2O+H]+ | C60H115NO5 |
| LIPID-P-1983 | Cer(t22:2/34:2(2OH)) | SP | Cer-AP | 854.80 | 871.80 | [M-H2O+H]+ | C56H105NO5 |
| LIPID-P-1984 | Cer(t22:2/36:1(2OH)) | SP | Cer-AP | 884.84 | 901.85 | [M-H2O+H]+ | C58H111NO5 |
| LIPID-P-1985 | Cer(t22:2/38:2(2OH)) | SP | Cer-AP | 910.86 | 927.86 | [M-H2O+H]+ | C60H113NO5 |
| LIPID-P-1986 | Cer(t22:2/40:2(2OH)) | SP | Cer-AP | 938.89 | 955.89 | [M-H2O+H]+ | C62H117NO5 |
| LIPID-P-1988 | Cer(t24:1/32:2(2OH)) | SP | Cer-AP | 856.81 | 873.81 | [M-H2O+H]+ | C56H107NO5 |
| LIPID-P-1979 | Cer(t21:2/35:1(2OH)) | SP | Cer-AP | 856.81 | 873.81 | [M-H2O+H]+ | C56H107NO5 |
| LIPID-P-1993 | Cer(t26:1/12:1(2OH)) | SP | Cer-AP | 606.55 | 623.55 | [M-H2O+H]+ | C38H73NO5 |
| LIPID-P-1994 | Cer(t26:1/30:2(2OH)) | SP | Cer-AP | 856.81 | 873.81 | [M-H2O+H]+ | C56H107NO5 |
| LIPID-P-1954 | Cer(t18:1/36:2(2OH)) | SP | Cer-AP | 828.78 | 845.78 | [M-H2O+H]+ | C54H103NO5 |
| LIPID-P-1995 | Cer(t26:2/30:2(2OH)) | SP | Cer-AP | 854.80 | 871.80 | [M-H2O+H]+ | C56H105NO5 |
| LIPID-P-1997 | Cer(t26:2/34:2(2OH)) | SP | Cer-AP | 910.86 | 927.86 | [M-H2O+H]+ | C60H113NO5 |
| LIPID-P-1998 | Cer(t28:2/30:2(2OH)) | SP | Cer-AP | 882.83 | 899.83 | [M-H2O+H]+ | C58H109NO5 |
| LIPID-P-1999 | Cer(t29:1/27:1(2OH)) | SP | Cer-AP | 858.83 | 875.83 | [M-H2O+H]+ | C56H109NO5 |
| LIPID-P-2000 | Cer(t30:0/18:1(2OH)) | SP | Cer-AP | 748.72 | 765.72 | [M-H2O+H]+ | C48H95NO5 |
| LIPID-P-2001 | Cer(t30:2/28:2(2OH)) | SP | Cer-AP | 882.83 | 899.83 | [M-H2O+H]+ | C58H109NO5 |
| LIPID-P-1978 | Cer(t20:2/40:2(2OH)) | SP | Cer-AP | 910.86 | 927.86 | [M-H2O+H]+ | C60H113NO5 |
| LIPID-P-1976 | Cer(t20:2/38:2(2OH)) | SP | Cer-AP | 882.83 | 899.83 | [M-H2O+H]+ | C58H109NO5 |
| LIPID-P-1975 | Cer(t20:2/38:1(2OH)) | SP | Cer-AP | 884.84 | 901.85 | [M-H2O+H]+ | C58H111NO5 |
| LIPID-P-1974 | Cer(t20:2/36:2(2OH)) | SP | Cer-AP | 854.80 | 871.80 | [M-H2O+H]+ | C56H105NO5 |
| LIPID-P-1973 | Cer(t20:2/36:1(2OH)) | SP | Cer-AP | 856.81 | 873.81 | [M-H2O+H]+ | C56H107NO5 |
| LIPID-P-1996 | Cer(t26:2/32:2(2OH)) | SP | Cer-AP | 882.83 | 899.83 | [M-H2O+H]+ | C58H109NO5 |
| LIPID-P-1953 | Cer(t18:1/36:1(2OH)) | SP | Cer-AP | 830.80 | 847.80 | [M-H2O+H]+ | C54H105NO5 |
| LIPID-P-1509 | Cer(t18:0/20:0(2OH)) | SP | Cer-AP | 628.58 | 627.58 | [M+H]+ | C38H77NO5 |
| LIPID-P-1951 | Cer(t18:1/20:1(2OH)) | SP | Cer-AP | 606.55 | 623.55 | [M-H2O+H]+ | C38H73NO5 |
| LIPID-P-1952 | Cer(t18:1/34:2(2OH)) | SP | Cer-AP | 800.75 | 817.75 | [M-H2O+H]+ | C52H99NO5 |
| LIPID-P-1508 | Cer(t18:0/18:0(2OH)) | SP | Cer-AP | 600.58 | 599.55 | [M+H]+ | C36H73NO5 |
| LIPID-P-1981 | Cer(t22:1/34:2(2OH)) | SP | Cer-AP | 856.81 | 873.81 | [M-H2O+H]+ | C56H107NO5 |
| LIPID-P-1980 | Cer(t22:1/16:1(2OH)) | SP | Cer-AP | 606.55 | 623.55 | [M-H2O+H]+ | C38H73NO5 |
| LIPID-P-1532 | Cer(t18:1/26:1(2OH)) | SP | Cer-AP | 708.65 | 707.64 | [M+H]+ | C44H85NO5 |
| LIPID-P-1950 | Cer(t18:0/22:2(2OH)) | SP | Cer-AP | 634.58 | 651.58 | [M-H2O+H]+ | C40H77NO5 |
| LIPID-P-2620 | Cer(d28:2/31:0(2OH)) | SP | Cer-AS | 884.88 | 901.88 | [M-H2O+H]+ | C59H115NO4 |
| LIPID-P-2621 | Cer(d28:2/31:1(2OH)) | SP | Cer-AS | 882.86 | 899.87 | [M-H2O+H]+ | C59H113NO4 |
| LIPID-P-2622 | Cer(d28:3/31:1(2OH)) | SP | Cer-AS | 880.85 | 897.85 | [M-H2O+H]+ | C59H111NO4 |
| LIPID-P-2623 | Cer(d29:1/28:0(2OH)) | SP | Cer-AS | 858.86 | 875.87 | [M-H2O+H]+ | C57H113NO4 |
| LIPID-P-2624 | Cer(d29:1/28:1(2OH)) | SP | Cer-AS | 856.85 | 873.85 | [M-H2O+H]+ | C57H111NO4 |
| LIPID-P-2625 | Cer(d29:1/28:2(2OH)) | SP | Cer-AS | 854.83 | 871.84 | [M-H2O+H]+ | C57H109NO4 |
| LIPID-P-2626 | Cer(d29:1/30:0(2OH)) | SP | Cer-AS | 886.89 | 903.90 | [M-H2O+H]+ | C59H117NO4 |
| LIPID-P-2598 | Cer(d25:3/34:1(2OH)) | SP | Cer-AS | 880.85 | 897.85 | [M-H2O+H]+ | C59H111NO4 |
| LIPID-P-2628 | Cer(d29:2/28:0(2OH)) | SP | Cer-AS | 856.85 | 873.85 | [M-H2O+H]+ | C57H111NO4 |
| LIPID-P-2629 | Cer(d29:2/30:0(2OH)) | SP | Cer-AS | 884.88 | 901.88 | [M-H2O+H]+ | C59H115NO4 |
| LIPID-P-2630 | Cer(d29:2/30:1(2OH)) | SP | Cer-AS | 882.86 | 899.87 | [M-H2O+H]+ | C59H113NO4 |
| LIPID-P-2631 | Cer(d29:2/30:2(2OH)) | SP | Cer-AS | 880.85 | 897.85 | [M-H2O+H]+ | C59H111NO4 |
| LIPID-P-2632 | Cer(d30:1/27:1(2OH)) | SP | Cer-AS | 856.85 | 873.85 | [M-H2O+H]+ | C57H111NO4 |
| LIPID-P-2633 | Cer(d30:1/29:0(2OH)) | SP | Cer-AS | 886.89 | 903.90 | [M-H2O+H]+ | C59H117NO4 |
| LIPID-P-2634 | Cer(d30:1/29:1(2OH)) | SP | Cer-AS | 884.88 | 901.88 | [M-H2O+H]+ | C59H115NO4 |
| LIPID-P-2635 | Cer(d30:2/29:0(2OH)) | SP | Cer-AS | 884.88 | 901.88 | [M-H2O+H]+ | C59H115NO4 |
| LIPID-P-2619 | Cer(d28:2/29:0(2OH)) | SP | Cer-AS | 856.85 | 873.85 | [M-H2O+H]+ | C57H111NO4 |
| LIPID-P-2627 | Cer(d29:1/30:2(2OH)) | SP | Cer-AS | 882.86 | 899.87 | [M-H2O+H]+ | C59H113NO4 |
| LIPID-P-2618 | Cer(d28:1/29:1(2OH)) | SP | Cer-AS | 856.85 | 873.85 | [M-H2O+H]+ | C57H111NO4 |
| LIPID-P-2616 | Cer(d27:3/32:0(2OH)) | SP | Cer-AS | 882.86 | 899.87 | [M-H2O+H]+ | C59H113NO4 |
| LIPID-P-2599 | Cer(d26:1/31:0(2OH)) | SP | Cer-AS | 858.86 | 875.87 | [M-H2O+H]+ | C57H113NO4 |
| LIPID-P-2600 | Cer(d26:1/31:1(2OH)) | SP | Cer-AS | 856.85 | 873.85 | [M-H2O+H]+ | C57H111NO4 |
| LIPID-P-2601 | Cer(d26:2/31:0(2OH)) | SP | Cer-AS | 856.85 | 873.85 | [M-H2O+H]+ | C57H111NO4 |
| LIPID-P-2636 | Cer(d30:2/29:1(2OH)) | SP | Cer-AS | 882.86 | 899.87 | [M-H2O+H]+ | C59H113NO4 |
| LIPID-P-2602 | Cer(d26:2/33:1(2OH)) | SP | Cer-AS | 882.86 | 899.87 | [M-H2O+H]+ | C59H113NO4 |
| LIPID-P-2603 | Cer(d26:3/33:0(2OH)) | SP | Cer-AS | 882.86 | 899.87 | [M-H2O+H]+ | C59H113NO4 |
| LIPID-P-2604 | Cer(d26:3/33:1(2OH)) | SP | Cer-AS | 880.85 | 897.85 | [M-H2O+H]+ | C59H111NO4 |
| LIPID-P-2605 | Cer(d26:3/35:1(2OH)) | SP | Cer-AS | 908.88 | 925.88 | [M-H2O+H]+ | C61H115NO4 |
| LIPID-P-2606 | Cer(d27:1/30:0(2OH)) | SP | Cer-AS | 858.86 | 875.87 | [M-H2O+H]+ | C57H113NO4 |
| LIPID-P-2607 | Cer(d27:1/30:1(2OH)) | SP | Cer-AS | 856.85 | 873.85 | [M-H2O+H]+ | C57H111NO4 |
| LIPID-P-2608 | Cer(d27:1/30:2(2OH)) | SP | Cer-AS | 854.83 | 871.84 | [M-H2O+H]+ | C57H109NO4 |
| LIPID-P-2609 | Cer(d27:1/32:2(2OH)) | SP | Cer-AS | 882.86 | 899.87 | [M-H2O+H]+ | C59H113NO4 |
| LIPID-P-2610 | Cer(d27:2/30:0(2OH)) | SP | Cer-AS | 856.85 | 873.85 | [M-H2O+H]+ | C57H111NO4 |
| LIPID-P-2611 | Cer(d27:2/30:1(2OH)) | SP | Cer-AS | 854.83 | 871.84 | [M-H2O+H]+ | C57H109NO4 |
| LIPID-P-2612 | Cer(d27:2/32:1(2OH)) | SP | Cer-AS | 882.86 | 899.87 | [M-H2O+H]+ | C59H113NO4 |
| LIPID-P-2617 | Cer(d28:1/27:1(2OH)) | SP | Cer-AS | 828.82 | 845.82 | [M-H2O+H]+ | C55H107NO4 |
| LIPID-P-2613 | Cer(d27:2/32:2(2OH)) | SP | Cer-AS | 880.85 | 897.85 | [M-H2O+H]+ | C59H111NO4 |
| LIPID-P-2595 | Cer(d25:2/34:2(2OH)) | SP | Cer-AS | 880.85 | 897.85 | [M-H2O+H]+ | C59H111NO4 |
| LIPID-P-2571 | Cer(d22:2/35:1(2OH)) | SP | Cer-AS | 854.83 | 871.84 | [M-H2O+H]+ | C57H109NO4 |
| LIPID-P-2570 | Cer(d22:1/35:1(2OH)) | SP | Cer-AS | 856.85 | 873.85 | [M-H2O+H]+ | C57H111NO4 |
| LIPID-P-2569 | Cer(d22:1/35:0(2OH)) | SP | Cer-AS | 858.86 | 875.87 | [M-H2O+H]+ | C57H113NO4 |
| LIPID-P-2568 | Cer(d22:1/23:1(2OH)) | SP | Cer-AS | 688.66 | 705.66 | [M-H2O+H]+ | C45H87NO4 |
| LIPID-P-2566 | Cer(d21:3/38:0(2OH)) | SP | Cer-AS | 882.86 | 899.87 | [M-H2O+H]+ | C59H113NO4 |
| LIPID-P-2565 | Cer(d21:2/38:2(2OH)) | SP | Cer-AS | 880.85 | 897.85 | [M-H2O+H]+ | C59H111NO4 |
| LIPID-P-2564 | Cer(d21:2/38:1(2OH)) | SP | Cer-AS | 882.86 | 899.87 | [M-H2O+H]+ | C59H113NO4 |
| LIPID-P-2573 | Cer(d23:2/34:0(2OH)) | SP | Cer-AS | 856.85 | 873.85 | [M-H2O+H]+ | C57H111NO4 |
| LIPID-P-2563 | Cer(d21:2/36:0(2OH)) | SP | Cer-AS | 856.85 | 873.85 | [M-H2O+H]+ | C57H111NO4 |
| LIPID-P-2561 | Cer(d21:1/38:1(2OH)) | SP | Cer-AS | 884.88 | 901.88 | [M-H2O+H]+ | C59H115NO4 |
| LIPID-P-2560 | Cer(d21:1/36:1(2OH)) | SP | Cer-AS | 856.85 | 873.85 | [M-H2O+H]+ | C57H111NO4 |
| LIPID-P-2559 | Cer(d21:1/36:0(2OH)) | SP | Cer-AS | 858.86 | 875.87 | [M-H2O+H]+ | C57H113NO4 |
| LIPID-P-2558 | Cer(d20:2/19:1(2OH)) | SP | Cer-AS | 602.55 | 619.55 | [M-H2O+H]+ | C39H73NO4 |
| LIPID-P-2554 | Cer(d20:1/23:1(2OH)) | SP | Cer-AS | 660.63 | 677.63 | [M-H2O+H]+ | C43H83NO4 |
| LIPID-P-2551 | Cer(d19:3/40:1(2OH)) | SP | Cer-AS | 880.85 | 897.85 | [M-H2O+H]+ | C59H111NO4 |
| LIPID-P-2550 | Cer(d19:3/40:0(2OH)) | SP | Cer-AS | 882.86 | 899.87 | [M-H2O+H]+ | C59H113NO4 |
| LIPID-P-2562 | Cer(d21:1/38:2(2OH)) | SP | Cer-AS | 882.86 | 899.87 | [M-H2O+H]+ | C59H113NO4 |
| LIPID-P-2574 | Cer(d23:2/36:0(2OH)) | SP | Cer-AS | 884.88 | 901.88 | [M-H2O+H]+ | C59H115NO4 |
| LIPID-P-2576 | Cer(d23:2/36:2(2OH)) | SP | Cer-AS | 880.85 | 897.85 | [M-H2O+H]+ | C59H111NO4 |
| LIPID-P-2594 | Cer(d25:2/34:1(2OH)) | SP | Cer-AS | 882.86 | 899.87 | [M-H2O+H]+ | C59H113NO4 |
| LIPID-P-2593 | Cer(d25:2/32:0(2OH)) | SP | Cer-AS | 856.85 | 873.85 | [M-H2O+H]+ | C57H111NO4 |
| LIPID-P-2592 | Cer(d25:1/34:2(2OH)) | SP | Cer-AS | 882.86 | 899.87 | [M-H2O+H]+ | C59H113NO4 |
| LIPID-P-2591 | Cer(d25:1/34:1(2OH)) | SP | Cer-AS | 884.88 | 901.88 | [M-H2O+H]+ | C59H115NO4 |
| LIPID-P-2590 | Cer(d25:1/32:1(2OH)) | SP | Cer-AS | 856.85 | 873.85 | [M-H2O+H]+ | C57H111NO4 |
| LIPID-P-2588 | Cer(d24:3/33:0(2OH)) | SP | Cer-AS | 854.83 | 871.84 | [M-H2O+H]+ | C57H109NO4 |
| LIPID-P-2586 | Cer(d24:3/15:1(2OH)) | SP | Cer-AS | 600.54 | 617.54 | [M-H2O+H]+ | C39H71NO4 |
| LIPID-P-2575 | Cer(d23:2/36:1(2OH)) | SP | Cer-AS | 882.86 | 899.87 | [M-H2O+H]+ | C59H113NO4 |
| LIPID-P-2585 | Cer(d24:2/35:1(2OH)) | SP | Cer-AS | 882.86 | 899.87 | [M-H2O+H]+ | C59H113NO4 |
| LIPID-P-2583 | Cer(d24:2/33:0(2OH)) | SP | Cer-AS | 856.85 | 873.85 | [M-H2O+H]+ | C57H111NO4 |
| LIPID-P-2582 | Cer(d24:1/35:1(2OH)) | SP | Cer-AS | 884.88 | 901.88 | [M-H2O+H]+ | C59H115NO4 |
| LIPID-P-2581 | Cer(d24:1/33:1(2OH)) | SP | Cer-AS | 856.85 | 873.85 | [M-H2O+H]+ | C57H111NO4 |
| LIPID-P-2580 | Cer(d24:1/33:0(2OH)) | SP | Cer-AS | 858.86 | 875.87 | [M-H2O+H]+ | C57H113NO4 |
| LIPID-P-2579 | Cer(d24:1/18:0(2OH)) | SP | Cer-AS | 648.63 | 665.63 | [M-H2O+H]+ | C42H83NO4 |
| LIPID-P-2578 | Cer(d23:3/36:1(2OH)) | SP | Cer-AS | 880.85 | 897.85 | [M-H2O+H]+ | C59H111NO4 |
| LIPID-P-2577 | Cer(d23:3/36:0(2OH)) | SP | Cer-AS | 882.86 | 899.87 | [M-H2O+H]+ | C59H113NO4 |
| LIPID-P-2584 | Cer(d24:2/35:0(2OH)) | SP | Cer-AS | 884.88 | 901.88 | [M-H2O+H]+ | C59H115NO4 |
| LIPID-P-2542 | Cer(d19:1/38:0(2OH)) | SP | Cer-AS | 858.86 | 875.87 | [M-H2O+H]+ | C57H113NO4 |
| LIPID-P-2536 | Cer(d18:2/42:2(2OH)) | SP | Cer-AS | 894.86 | 911.87 | [M-H2O+H]+ | C60H113NO4 |
| LIPID-P-2535 | Cer(d18:2/40:2(2OH)) | SP | Cer-AS | 866.83 | 883.84 | [M-H2O+H]+ | C58H109NO4 |
| LIPID-P-2495 | Cer(d17:2/42:1(2OH)) | SP | Cer-AS | 882.86 | 899.87 | [M-H2O+H]+ | C59H113NO4 |
| LIPID-P-2494 | Cer(d17:2/42:0(2OH)) | SP | Cer-AS | 884.88 | 901.88 | [M-H2O+H]+ | C59H115NO4 |
| LIPID-P-2493 | Cer(d17:2/40:0(2OH)) | SP | Cer-AS | 856.85 | 873.85 | [M-H2O+H]+ | C57H111NO4 |
| LIPID-P-2492 | Cer(d17:2/22:1(2OH)) | SP | Cer-AS | 602.55 | 619.55 | [M-H2O+H]+ | C39H73NO4 |
| LIPID-P-2490 | Cer(d17:1/42:1(2OH)) | SP | Cer-AS | 884.88 | 901.88 | [M-H2O+H]+ | C59H115NO4 |
| LIPID-P-2597 | Cer(d25:3/34:0(2OH)) | SP | Cer-AS | 882.86 | 899.87 | [M-H2O+H]+ | C59H113NO4 |
| LIPID-P-2496 | Cer(d17:3/22:0(2OH)) | SP | Cer-AS | 602.55 | 619.55 | [M-H2O+H]+ | C39H73NO4 |
| LIPID-P-2487 | Cer(d17:1/40:0(2OH)) | SP | Cer-AS | 858.86 | 875.87 | [M-H2O+H]+ | C57H113NO4 |
| LIPID-P-2485 | Cer(d17:1/22:2(2OH)) | SP | Cer-AS | 602.55 | 619.55 | [M-H2O+H]+ | C39H73NO4 |
| LIPID-P-2478 | Cer(d15:2/42:0(2OH)) | SP | Cer-AS | 856.85 | 873.85 | [M-H2O+H]+ | C57H111NO4 |
| LIPID-P-2477 | Cer(d15:1/42:2(2OH)) | SP | Cer-AS | 854.83 | 871.84 | [M-H2O+H]+ | C57H109NO4 |
| LIPID-P-2476 | Cer(d15:1/42:1(2OH)) | SP | Cer-AS | 856.85 | 873.85 | [M-H2O+H]+ | C57H111NO4 |
| LIPID-P-2475 | Cer(d15:1/42:0(2OH)) | SP | Cer-AS | 858.86 | 875.87 | [M-H2O+H]+ | C57H113NO4 |
| LIPID-P-2474 | Cer(d14:3/27:1(2OH)) | SP | Cer-AS | 628.57 | 645.57 | [M-H2O+H]+ | C41H75NO4 |
| LIPID-P-2486 | Cer(d17:1/38:0(2OH)) | SP | Cer-AS | 830.83 | 847.84 | [M-H2O+H]+ | C55H109NO4 |
| LIPID-P-2497 | Cer(d17:3/42:1(2OH)) | SP | Cer-AS | 880.85 | 897.85 | [M-H2O+H]+ | C59H111NO4 |
| LIPID-P-2498 | Cer(d17:3/42:2(2OH)) | SP | Cer-AS | 878.83 | 895.84 | [M-H2O+H]+ | C59H109NO4 |
| LIPID-P-2501 | Cer(d18:1/15:1(2OH)) | SP | Cer-AS | 520.47 | 537.48 | [M-H2O+H]+ | C33H63NO4 |
| LIPID-P-2534 | Cer(d18:2/40:1(2OH)) | SP | Cer-AS | 868.85 | 885.85 | [M-H2O+H]+ | C58H111NO4 |
| LIPID-P-2533 | Cer(d18:2/24:0(2OH)) | SP | Cer-AS | 646.61 | 663.62 | [M-H2O+H]+ | C42H81NO4 |
| LIPID-P-2532 | Cer(d18:2/23:1(2OH)) | SP | Cer-AS | 630.58 | 647.59 | [M-H2O+H]+ | C41H77NO4 |
| LIPID-P-2528 | Cer(d18:2/15:0(2OH)) | SP | Cer-AS | 520.47 | 537.48 | [M-H2O+H]+ | C33H63NO4 |
| LIPID-P-2527 | Cer(d18:1/42:2(2OH)) | SP | Cer-AS | 896.88 | 913.88 | [M-H2O+H]+ | C60H115NO4 |
| LIPID-P-2526 | Cer(d18:1/42:1(2OH)) | SP | Cer-AS | 898.89 | 915.90 | [M-H2O+H]+ | C60H117NO4 |
| LIPID-P-2525 | Cer(d18:1/40:2(2OH)) | SP | Cer-AS | 868.85 | 885.85 | [M-H2O+H]+ | C58H111NO4 |
| LIPID-P-2524 | Cer(d18:1/40:1(2OH)) | SP | Cer-AS | 870.86 | 887.87 | [M-H2O+H]+ | C58H113NO4 |
| LIPID-P-2523 | Cer(d18:1/40:0(2OH)) | SP | Cer-AS | 872.88 | 889.88 | [M-H2O+H]+ | C58H115NO4 |
| LIPID-P-2522 | Cer(d18:1/38:2(2OH)) | SP | Cer-AS | 840.82 | 857.82 | [M-H2O+H]+ | C56H107NO4 |
| LIPID-P-2511 | Cer(d18:1/21:1(2OH)) | SP | Cer-AS | 604.57 | 621.57 | [M-H2O+H]+ | C39H75NO4 |
| LIPID-P-2510 | Cer(d18:1/21:0(2OH)) | SP | Cer-AS | 606.58 | 623.59 | [M-H2O+H]+ | C39H77NO4 |
| LIPID-P-2508 | Cer(d18:1/20:0(2OH)) | SP | Cer-AS | 592.57 | 609.57 | [M-H2O+H]+ | C38H75NO4 |
| LIPID-P-2506 | Cer(d18:1/18:0(2OH)) | SP | Cer-AS | 564.54 | 581.54 | [M-H2O+H]+ | C36H71NO4 |
| LIPID-P-2596 | Cer(d25:2/36:1(2OH)) | SP | Cer-AS | 910.89 | 927.90 | [M-H2O+H]+ | C61H117NO4 |
| LIPID-P-2488 | Cer(d17:1/40:1(2OH)) | SP | Cer-AS | 856.85 | 873.85 | [M-H2O+H]+ | C57H111NO4 |
| LIPID-P-2637 | Cer(d30:3/29:0(2OH)) | SP | Cer-AS | 882.86 | 899.87 | [M-H2O+H]+ | C59H113NO4 |
| LIPID-P-2032 | Cer(d25:0/18:0) | SP | Cer-NDS | 648.67 | 665.67 | [M-H2O+H]+ | C43H87NO3 |
| LIPID-P-2029 | Cer(d24:0/18:0) | SP | Cer-NDS | 634.65 | 651.65 | [M-H2O+H]+ | C42H85NO3 |
| LIPID-P-2027 | Cer(d23:0/18:0) | SP | Cer-NDS | 620.63 | 637.64 | [M-H2O+H]+ | C41H83NO3 |
| LIPID-P-2020 | Cer(d18:0/23:0) | SP | Cer-NDS | 620.63 | 637.64 | [M-H2O+H]+ | C41H83NO3 |
| LIPID-P-2017 | Cer(d18:0/18:1) | SP | Cer-NDS | 548.54 | 565.54 | [M-H2O+H]+ | C36H71NO3 |
| LIPID-P-2013 | Cer(d17:0/18:0) | SP | Cer-NDS | 536.54 | 553.54 | [M-H2O+H]+ | C35H71NO3 |
| LIPID-P-2007 | Cer(d16:0/18:2) | SP | Cer-NDS | 518.49 | 535.50 | [M-H2O+H]+ | C34H65NO3 |
| LIPID-P-2006 | Cer(d16:0/18:1) | SP | Cer-NDS | 520.51 | 537.51 | [M-H2O+H]+ | C34H67NO3 |
| LIPID-P-2404 | Cer(t16:1/25:0) | SP | Cer-NP | 634.61 | 651.62 | [M-H2O+H]+ | C41H81NO4 |
| LIPID-P-2465 | Cer(t24:0/18:0) | SP | Cer-NP | 650.64 | 667.65 | [M-H2O+H]+ | C42H85NO4 |
| LIPID-P-2455 | Cer(t18:1/32:1) | SP | Cer-NP | 758.74 | 775.74 | [M-H2O+H]+ | C50H97NO4 |
| LIPID-P-2393 | Cer(t14:1/21:0) | SP | Cer-NP | 550.52 | 567.52 | [M-H2O+H]+ | C35H69NO4 |
| LIPID-P-2451 | Cer(t18:1/15:1) | SP | Cer-NP | 520.47 | 537.48 | [M-H2O+H]+ | C33H63NO4 |
| LIPID-P-2446 | Cer(t18:0/23:1) | SP | Cer-NP | 634.61 | 651.62 | [M-H2O+H]+ | C41H81NO4 |
| LIPID-P-2445 | Cer(t18:0/22:0) | SP | Cer-NP | 640.62 | 639.62 | [M+H]+ | C40H81NO4 |
| LIPID-P-2452 | Cer(t18:1/17:0) | SP | Cer-NP | 550.52 | 567.52 | [M-H2O+H]+ | C35H69NO4 |
| LIPID-P-2438 | Cer(t17:2/35:0) | SP | Cer-NP | 786.77 | 803.77 | [M-H2O+H]+ | C52H101NO4 |
| LIPID-P-2439 | Cer(t17:2/35:1) | SP | Cer-NP | 784.75 | 801.76 | [M-H2O+H]+ | C52H99NO4 |
| LIPID-P-2411 | Cer(t17:0/22:2) | SP | Cer-NP | 604.57 | 621.57 | [M-H2O+H]+ | C39H75NO4 |
| LIPID-P-1548 | Cer(t18:1/24:0) | SP | Cer-NP | 666.64 | 665.63 | [M+H]+ | C42H83NO4 |
| LIPID-P-2418 | Cer(t17:1/22:1) | SP | Cer-NP | 604.57 | 621.57 | [M-H2O+H]+ | C39H75NO4 |
| LIPID-P-2419 | Cer(t17:1/22:2) | SP | Cer-NP | 602.55 | 619.55 | [M-H2O+H]+ | C39H73NO4 |
| LIPID-P-2406 | Cer(t16:1/26:0) | SP | Cer-NP | 648.63 | 665.63 | [M-H2O+H]+ | C42H83NO4 |
| LIPID-P-2433 | Cer(t17:2/24:1) | SP | Cer-NP | 630.58 | 647.59 | [M-H2O+H]+ | C41H77NO4 |
| LIPID-P-2434 | Cer(t17:2/25:0) | SP | Cer-NP | 646.61 | 663.62 | [M-H2O+H]+ | C42H81NO4 |
| LIPID-P-2436 | Cer(t17:2/31:0) | SP | Cer-NP | 730.71 | 747.71 | [M-H2O+H]+ | C48H93NO4 |
| LIPID-P-2437 | Cer(t17:2/33:0) | SP | Cer-NP | 758.74 | 775.74 | [M-H2O+H]+ | C50H97NO4 |
| LIPID-P-2430 | Cer(t17:2/22:1) | SP | Cer-NP | 602.55 | 619.55 | [M-H2O+H]+ | C39H73NO4 |
| LIPID-P-0140 | Cer(d18:2/23:0) | SP | Cer-NS | 634.60 | 633.61 | [M+H]+ | C41H79NO3 |
| LIPID-P-0139 | Cer(d18:2/22:0) | SP | Cer-NS | 620.60 | 619.59 | [M+H]+ | C40H77NO3 |
| LIPID-P-0138 | Cer(d18:2/21:0) | SP | Cer-NS | 606.60 | 605.57 | [M+H]+ | C39H75NO3 |
| LIPID-P-0137 | Cer(d18:2/20:0) | SP | Cer-NS | 592.60 | 591.56 | [M+H]+ | C38H73NO3 |
| LIPID-P-0135 | Cer(d18:2/17:0) | SP | Cer-NS | 550.50 | 549.51 | [M+H]+ | C35H67NO3 |
| LIPID-P-0134 | Cer(d18:2/16:0) | SP | Cer-NS | 536.50 | 535.50 | [M+H]+ | C34H65NO3 |
| LIPID-P-0132 | Cer(d16:1/24:1) | SP | Cer-NS | 620.60 | 619.59 | [M+H]+ | C40H77NO3 |
| LIPID-P-0141 | Cer(d18:2/24:0) | SP | Cer-NS | 648.60 | 647.62 | [M+H]+ | C42H81NO3 |
| LIPID-P-0136 | Cer(d18:2/18:0) | SP | Cer-NS | 564.60 | 563.53 | [M+H]+ | C36H69NO3 |
| LIPID-P-0142 | Cer(d18:2/24:1) | SP | Cer-NS | 646.60 | 645.61 | [M+H]+ | C42H79NO3 |
| LIPID-P-2087 | Cer(d18:2/26:1) | SP | Cer-NS | 656.63 | 673.64 | [M-H2O+H]+ | C44H83NO3 |
| LIPID-P-2082 | Cer(d18:2/19:0) | SP | Cer-NS | 560.54 | 577.54 | [M-H2O+H]+ | C37H71NO3 |
| LIPID-P-2083 | Cer(d18:2/22:1) | SP | Cer-NS | 600.57 | 617.57 | [M-H2O+H]+ | C40H75NO3 |
| LIPID-P-2084 | Cer(d18:2/24:2) | SP | Cer-NS | 626.59 | 643.59 | [M-H2O+H]+ | C42H77NO3 |
| LIPID-P-2085 | Cer(d18:2/25:0) | SP | Cer-NS | 644.63 | 661.64 | [M-H2O+H]+ | C43H83NO3 |
| LIPID-P-2086 | Cer(d18:2/25:1) | SP | Cer-NS | 642.62 | 659.62 | [M-H2O+H]+ | C43H81NO3 |
| LIPID-P-2106 | Cer(d20:1/25:0) | SP | Cer-NS | 674.68 | 691.68 | [M-H2O+H]+ | C45H89NO3 |
| LIPID-P-2108 | Cer(d20:1/26:0) | SP | Cer-NS | 688.70 | 705.70 | [M-H2O+H]+ | C46H91NO3 |
| LIPID-P-2113 | Cer(d20:2/24:0) | SP | Cer-NS | 658.65 | 675.65 | [M-H2O+H]+ | C44H85NO3 |
| LIPID-P-2116 | Cer(d21:3/16:0) | SP | Cer-NS | 558.52 | 575.53 | [M-H2O+H]+ | C37H69NO3 |
| LIPID-P-2117 | Cer(d22:1/26:0) | SP | Cer-NS | 716.73 | 733.73 | [M-H2O+H]+ | C48H95NO3 |
| LIPID-P-0143 | Cer(d18:2/26:0) | SP | Cer-NS | 676.60 | 675.65 | [M+H]+ | C44H85NO3 |
| LIPID-P-0130 | Cer(d16:1/23:0) | SP | Cer-NS | 608.60 | 607.59 | [M+H]+ | C39H77NO3 |
| LIPID-P-0131 | Cer(d16:1/24:0) | SP | Cer-NS | 622.60 | 621.61 | [M+H]+ | C40H79NO3 |
| LIPID-P-0128 | Cer(d16:1/20:0) | SP | Cer-NS | 566.60 | 565.54 | [M+H]+ | C36H71NO3 |
| LIPID-P-2064 | Cer(d17:1/25:0) | SP | Cer-NS | 632.63 | 649.64 | [M-H2O+H]+ | C42H83NO3 |
| LIPID-P-2062 | Cer(d17:1/24:1) | SP | Cer-NS | 616.60 | 633.61 | [M-H2O+H]+ | C41H79NO3 |
| LIPID-P-0129 | Cer(d16:1/22:0) | SP | Cer-NS | 594.60 | 593.57 | [M+H]+ | C38H75NO3 |
| LIPID-P-2060 | Cer(d17:1/23:1) | SP | Cer-NS | 602.59 | 619.59 | [M-H2O+H]+ | C40H77NO3 |
| LIPID-P-2055 | Cer(d17:1/20:0) | SP | Cer-NS | 562.56 | 579.56 | [M-H2O+H]+ | C37H73NO3 |
| LIPID-P-2047 | Cer(d16:1/26:0) | SP | Cer-NS | 632.63 | 649.64 | [M-H2O+H]+ | C42H83NO3 |
| LIPID-P-0118 | Cer(d18:1/25:0) | SP | Cer-NS | 664.66 | 663.65 | [M+H]+ | C43H85NO3 |
| LIPID-P-0117 | Cer(d18:1/24:0) | SP | Cer-NS | 650.65 | 649.64 | [M+H]+ | C42H83NO3 |
| LIPID-P-0116 | Cer(d18:1/23:0) | SP | Cer-NS | 636.63 | 635.62 | [M+H]+ | C41H81NO3 |
| LIPID-P-0115 | Cer(d18:1/22:0) | SP | Cer-NS | 622.61 | 621.61 | [M+H]+ | C40H79NO3 |
| LIPID-P-0114 | Cer(d18:1/21:0) | SP | Cer-NS | 608.60 | 607.59 | [M+H]+ | C39H77NO3 |
| LIPID-P-0113 | Cer(d18:1/20:0) | SP | Cer-NS | 594.58 | 593.57 | [M+H]+ | C38H75NO3 |
| LIPID-P-0112 | Cer(d18:1/19:0) | SP | Cer-NS | 580.60 | 579.56 | [M+H]+ | C37H73NO3 |
| LIPID-P-0111 | Cer(d18:1/18:0) | SP | Cer-NS | 566.55 | 565.54 | [M+H]+ | C36H71NO3 |
| LIPID-P-0110 | Cer(d18:1/17:0) | SP | Cer-NS | 552.54 | 551.53 | [M+H]+ | C35H69NO3 |
| LIPID-P-0109 | Cer(d18:1/16:0) | SP | Cer-NS | 538.52 | 537.51 | [M+H]+ | C34H67NO3 |
| LIPID-P-0108 | Cer(d18:1/14:0) | SP | Cer-NS | 510.49 | 509.48 | [M+H]+ | C32H63NO3 |
| LIPID-P-2065 | Cer(d17:1/25:1) | SP | Cer-NS | 630.62 | 647.62 | [M-H2O+H]+ | C42H81NO3 |
| LIPID-P-2069 | Cer(d17:2/24:1) | SP | Cer-NS | 614.59 | 631.59 | [M-H2O+H]+ | C41H77NO3 |
| LIPID-P-2067 | Cer(d17:1/26:2) | SP | Cer-NS | 642.62 | 659.62 | [M-H2O+H]+ | C43H81NO3 |
| LIPID-P-2077 | Cer(d18:1/26:2) | SP | Cer-NS | 656.63 | 673.64 | [M-H2O+H]+ | C44H83NO3 |
| LIPID-P-2078 | Cer(d18:1/28:0) | SP | Cer-NS | 688.70 | 705.70 | [M-H2O+H]+ | C46H91NO3 |
| LIPID-P-0120 | Cer(d18:1/16:1) | SP | Cer-NS | 536.50 | 535.50 | [M+H]+ | C34H65NO3 |
| LIPID-P-0121 | Cer(d18:1/18:1) | SP | Cer-NS | 564.54 | 563.53 | [M+H]+ | C36H69NO3 |
| LIPID-P-0122 | Cer(d18:1/20:1) | SP | Cer-NS | 592.57 | 591.56 | [M+H]+ | C38H73NO3 |
| LIPID-P-0123 | Cer(d18:1/22:1) | SP | Cer-NS | 620.60 | 619.59 | [M+H]+ | C40H77NO3 |
| LIPID-P-2076 | Cer(d18:1/25:1) | SP | Cer-NS | 644.63 | 661.64 | [M-H2O+H]+ | C43H83NO3 |
| LIPID-P-0124 | Cer(d18:1/24:1) | SP | Cer-NS | 648.63 | 647.62 | [M+H]+ | C42H81NO3 |
| LIPID-P-0126 | Cer(d16:1/16:0) | SP | Cer-NS | 510.60 | 509.48 | [M+H]+ | C32H63NO3 |
| LIPID-P-2074 | Cer(d18:1/23:1) | SP | Cer-NS | 616.60 | 633.61 | [M-H2O+H]+ | C41H79NO3 |
| LIPID-P-2073 | Cer(d18:1/22:2) | SP | Cer-NS | 600.57 | 617.57 | [M-H2O+H]+ | C40H75NO3 |
| LIPID-P-2072 | Cer(d18:1/18:2) | SP | Cer-NS | 544.51 | 561.51 | [M-H2O+H]+ | C36H67NO3 |
| LIPID-P-0125 | Cer(d18:1/26:1) | SP | Cer-NS | 676.66 | 675.65 | [M+H]+ | C44H85NO3 |
| LIPID-P-2075 | Cer(d18:1/24:2) | SP | Cer-NS | 628.60 | 645.61 | [M-H2O+H]+ | C42H79NO3 |
| LIPID-N-0613 | CerP(d18:1/20:3) | SP | CerP | 666.49 | 667.49 | [M-H]- | C38H70NO6P |
| LIPID-N-0609 | CerP(d18:1/18:1) | SP | CerP | 642.49 | 643.49 | [M-H]- | C36H70NO6P |
| LIPID-P-0052 | Cholesterol | ST | Cholesterol | 369.40 | 386.35 | [M-H2O+H]+ | C27H46O |
| LIPID-P-0161 | CoenzymeQ10 | PR | CoQ | 863.69 | 862.68 | [M+H]+ | C59H90O4 |
| LIPID-P-0159 | CoenzymeQ8 | PR | CoQ | 727.57 | 780.61 | [M+H]+ | C53H80O4 |
| LIPID-P-0160 | CoenzymeQ9 | PR | CoQ | 795.63 | 794.62 | [M+H]+ | C54H82O4 |
| LIPID-P-0167 | DG(14:0_16:0) | GL | DG | 558.52 | 540.48 | [M+NH4]+ | C33H64O5 |
| LIPID-P-0169 | DG(16:0_16:0) | GL | DG | 586.55 | 568.51 | [M+NH4]+ | C35H68O5 |
| LIPID-P-0182 | DG(21:0_16:0) | GL | DG | 656.61 | 638.58 | [M+NH4]+ | C40H78O5 |
| LIPID-P-0181 | DG(19:0_18:0) | GL | DG | 656.61 | 638.58 | [M+NH4]+ | C40H78O5 |
| LIPID-P-0178 | DG(18:0_18:0) | GL | DG | 642.61 | 624.57 | [M+NH4]+ | C39H76O5 |
| LIPID-P-0174 | DG(16:0_18:0) | GL | DG | 614.58 | 596.54 | [M+NH4]+ | C37H72O5 |
| LIPID-P-0171 | DG(12:0_20:0) | GL | DG | 586.55 | 568.51 | [M+NH4]+ | C35H68O5 |
| LIPID-P-0170 | DG(14:0_18:0) | GL | DG | 586.55 | 568.51 | [M+NH4]+ | C35H68O5 |
| LIPID-P-0191 | DG(14:0_18:1) | GL | DG | 584.53 | 566.49 | [M+NH4]+ | C35H66O5 |
| LIPID-P-0238 | DG(16:1_18:3) | GL | DG | 606.52 | 588.48 | [M+NH4]+ | C37H64O5 |
| LIPID-P-0239 | DG(16:0_20:4) | GL | DG | 634.55 | 616.51 | [M+NH4]+ | C39H68O5 |
| LIPID-P-0240 | DG(18:2_18:2) | GL | DG | 634.55 | 616.51 | [M+NH4]+ | C39H68O5 |
| LIPID-P-0241 | DG(16:1_20:3) | GL | DG | 634.54 | 616.51 | [M+NH4]+ | C39H68O5 |
| LIPID-P-0242 | DG(18:1_20:3) | GL | DG | 662.58 | 644.54 | [M+NH4]+ | C41H72O5 |
| LIPID-P-0188 | DG(14:0_16:1) | GL | DG | 556.50 | 538.46 | [M+NH4]+ | C33H62O5 |
| LIPID-P-0247 | DG(18:2_18:3) | GL | DG | 632.53 | 614.49 | [M+NH4]+ | C39H66O5 |
| LIPID-P-0248 | DG(16:1_20:4) | GL | DG | 632.53 | 614.49 | [M+NH4]+ | C39H66O5 |
| LIPID-P-0252 | DG(18:1_20:4) | GL | DG | 660.57 | 642.52 | [M+NH4]+ | C41H70O5 |
| LIPID-P-0253 | DG(16:0_22:5) | GL | DG | 660.56 | 642.52 | [M+NH4]+ | C41H70O5 |
| LIPID-P-0257 | DG(18:1_22:4) | GL | DG | 688.60 | 670.55 | [M+NH4]+ | C43H74O5 |
| LIPID-P-0259 | DG(18:2_20:4) | GL | DG | 658.55 | 640.51 | [M+NH4]+ | C41H68O5 |
| LIPID-P-0261 | DG(16:0_22:6) | GL | DG | 658.55 | 640.51 | [M+NH4]+ | C41H68O5 |
| LIPID-P-0268 | DG(16:1_22:6) | GL | DG | 656.52 | 638.49 | [M+NH4]+ | C41H66O5 |
| LIPID-P-0269 | DG(18:1_22:6) | GL | DG | 684.56 | 666.52 | [M+NH4]+ | C43H70O5 |
| LIPID-P-1437 | DG(12:0_18:2) | GL | DG | 554.48 | 536.44 | [M+NH4]+ | C33H60O5 |
| LIPID-P-1439 | DG(15:0_18:2) | GL | DG | 596.53 | 578.49 | [M+NH4]+ | C36H66O5 |
| LIPID-P-1440 | DG(17:0_18:2) | GL | DG | 624.56 | 606.52 | [M+NH4]+ | C38H70O5 |
| LIPID-P-1443 | DG(20:0_18:2) | GL | DG | 666.60 | 648.57 | [M+NH4]+ | C41H76O5 |
| LIPID-P-1451 | DG(14:1_18:2) | GL | DG | 580.49 | 562.46 | [M+NH4]+ | C35H62O5 |
| LIPID-P-0234 | DG(18:0_20:3) | GL | DG | 664.59 | 646.55 | [M+NH4]+ | C41H74O5 |
| LIPID-P-0233 | DG(18:1_20:2) | GL | DG | 664.60 | 646.55 | [M+NH4]+ | C41H74O5 |
| LIPID-P-0243 | DG(18:0_20:4) | GL | DG | 662.58 | 644.54 | [M+NH4]+ | C41H72O5 |
| LIPID-P-0230 | DG(14:1_22:2) | GL | DG | 636.57 | 618.52 | [M+NH4]+ | C39H70O5 |
| LIPID-P-0196 | DG(16:0_18:1) | GL | DG | 612.57 | 594.52 | [M+NH4]+ | C37H70O5 |
| LIPID-P-0195 | DG(16:1_18:0) | GL | DG | 612.57 | 594.52 | [M+NH4]+ | C37H70O5 |
| LIPID-P-0193 | DG(16:0_17:1) | GL | DG | 598.53 | 580.51 | [M+NH4]+ | C36H68O5 |
| LIPID-P-0192 | DG(16:0_16:1) | GL | DG | 584.53 | 566.49 | [M+NH4]+ | C35H66O5 |
| LIPID-P-0232 | DG(18:2_20:1) | GL | DG | 664.60 | 646.55 | [M+NH4]+ | C41H74O5 |
| LIPID-P-0200 | DG(16:0_20:1) | GL | DG | 640.60 | 622.55 | [M+NH4]+ | C39H74O5 |
| LIPID-P-0201 | DG(18:0_18:1) | GL | DG | 640.60 | 622.55 | [M+NH4]+ | C39H74O5 |
| LIPID-P-0205 | DG(18:1_20:0) | GL | DG | 668.63 | 650.58 | [M+NH4]+ | C41H78O5 |
| LIPID-P-0210 | DG(14:0_18:2) | GL | DG | 582.52 | 564.48 | [M+NH4]+ | C35H64O5 |
| LIPID-P-0197 | DG(17:0_18:1) | GL | DG | 626.57 | 608.54 | [M+NH4]+ | C38H72O5 |
| LIPID-P-0212 | DG(16:1_17:1) | GL | DG | 596.52 | 578.49 | [M+NH4]+ | C36H66O5 |
| LIPID-P-0211 | DG(16:1_16:1) | GL | DG | 582.52 | 564.48 | [M+NH4]+ | C35H64O5 |
| LIPID-P-0214 | DG(16:0_18:2) | GL | DG | 610.55 | 592.51 | [M+NH4]+ | C37H68O5 |
| LIPID-P-0215 | DG(17:1_18:1) | GL | DG | 624.55 | 606.52 | [M+NH4]+ | C38H70O5 |
| LIPID-P-0217 | DG(18:0_18:2) | GL | DG | 638.58 | 620.54 | [M+NH4]+ | C39H72O5 |
| LIPID-P-0218 | DG(18:1_18:1) | GL | DG | 638.58 | 620.54 | [M+NH4]+ | C39H72O5 |
| LIPID-P-0219 | DG(18:1_20:1) | GL | DG | 666.61 | 648.57 | [M+NH4]+ | C41H76O5 |
| LIPID-P-0224 | DG(16:1_18:2) | GL | DG | 608.53 | 590.49 | [M+NH4]+ | C37H66O5 |
| LIPID-P-0225 | DG(16:0_18:3) | GL | DG | 608.53 | 590.49 | [M+NH4]+ | C37H66O5 |
| LIPID-P-0227 | DG(16:1_20:2) | GL | DG | 636.57 | 618.52 | [M+NH4]+ | C39H70O5 |
| LIPID-P-0228 | DG(16:0_20:3) | GL | DG | 636.57 | 618.52 | [M+NH4]+ | C39H70O5 |
| LIPID-P-0229 | DG(18:1_18:2) | GL | DG | 636.57 | 618.52 | [M+NH4]+ | C39H70O5 |
| LIPID-P-0213 | DG(16:1_18:1) | GL | DG | 610.55 | 592.51 | [M+NH4]+ | C37H68O5 |
| LIPID-P-1676 | DG(O-20:0_18:2) | GL | DG-O | 652.62 | 634.59 | [M+NH4]+ | C41H78O4 |
| LIPID-P-1643 | DG(O-21:0_16:0) | GL | DG-O | 642.64 | 624.61 | [M+NH4]+ | C40H80O4 |
| LIPID-P-1639 | DG(O-19:0_16:0) | GL | DG-O | 614.61 | 596.57 | [M+NH4]+ | C38H76O4 |
| LIPID-P-1693 | DG(O-20:0_22:4) | GL | DG-O | 704.66 | 686.62 | [M+NH4]+ | C45H82O4 |
| LIPID-P-1691 | DG(O-19:2_18:2) | GL | DG-O | 634.58 | 616.54 | [M+NH4]+ | C40H72O4 |
| LIPID-P-1678 | DG(O-19:2_20:0) | GL | DG-O | 666.64 | 648.61 | [M+NH4]+ | C42H80O4 |
| LIPID-N-0026 | 9,10-DiHOME | FA | Eicosanoid | 313.24 | 314.25 | [M-H]- | C18H34O4 |
| LIPID-N-0028 | (±)12-HEPE | FA | Eicosanoid | 317.21 | 318.22 | [M-H]- | C20H30O3 |
| LIPID-N-0034 | (±)12-HETE | FA | Eicosanoid | 319.23 | 320.24 | [M-H]- | C20H32O3 |
| LIPID-N-0035 | (±)15-HETE | FA | Eicosanoid | 319.23 | 320.24 | [M-H]- | C20H32O3 |
| LIPID-N-0041 | 11(S)-HETE | FA | Eicosanoid | 319.23 | 320.24 | [M-H]- | C20H32O3 |
| LIPID-N-0050 | 14(S)-HDHA | FA | Eicosanoid | 343.23 | 344.24 | [M-H]- | C22H32O3 |
| LIPID-N-0055 | PGD2 | FA | Eicosanoid | 351.22 | 352.22 | [M-H]- | C20H32O5 |
| LIPID-N-0023 | 9,10-EpOME | FA | Eicosanoid | 295.23 | 296.24 | [M-H]- | C18H32O3 |
| LIPID-N-0062 | TxB3 | FA | Eicosanoid | 367.21 | 368.22 | [M-H]- | C20H32O6 |
| LIPID-N-0056 | PGE2 | FA | Eicosanoid | 351.22 | 352.22 | [M-H]- | C20H32O5 |
| LIPID-N-0022 | 12,13-EpOME | FA | Eicosanoid | 295.23 | 296.24 | [M-H]- | C18H32O3 |
| LIPID-N-0074 | FFA(17:0) | FA | FFA | 269.25 | 270.26 | [M-H]- | C17H34O2 |
| LIPID-N-0075 | FFA(18:0) | FA | FFA | 283.26 | 284.27 | [M-H]- | C18H36O2 |
| LIPID-N-0101 | FFA(20:5) | FA | FFA | 301.22 | 302.22 | [M-H]- | C20H30O2 |
| LIPID-N-0094 | FFA(18:3) | FA | FFA | 277.22 | 278.22 | [M-H]- | C18H30O2 |
| LIPID-N-0081 | FFA(15:1) | FA | FFA | 239.20 | 240.21 | [M-H]- | C15H28O2 |
| LIPID-N-0082 | FFA(16:1) | FA | FFA | 253.22 | 254.22 | [M-H]- | C16H30O2 |
| LIPID-N-0083 | FFA(17:1) | FA | FFA | 267.23 | 268.24 | [M-H]- | C17H32O2 |
| LIPID-N-0084 | FFA(18:1) | FA | FFA | 281.25 | 282.26 | [M-H]- | C18H34O2 |
| LIPID-N-0099 | FFA(22:4) | FA | FFA | 331.26 | 332.27 | [M-H]- | C22H36O2 |
| LIPID-N-0105 | FFA(24:6) | FA | FFA | 355.26 | 356.27 | [M-H]- | C24H36O2 |
| LIPID-N-0104 | FFA(22:6) | FA | FFA | 327.23 | 328.24 | [M-H]- | C22H32O2 |
| LIPID-N-0103 | FFA(24:5) | FA | FFA | 357.28 | 358.29 | [M-H]- | C24H38O2 |
| LIPID-N-0102 | FFA(22:5) | FA | FFA | 329.25 | 330.26 | [M-H]- | C22H34O2 |
| LIPID-N-0085 | FFA(19:1) | FA | FFA | 295.26 | 296.27 | [M-H]- | C19H36O2 |
| LIPID-N-0086 | FFA(20:1) | FA | FFA | 309.28 | 310.29 | [M-H]- | C20H38O2 |
| LIPID-N-0087 | FFA(22:1) | FA | FFA | 337.31 | 338.32 | [M-H]- | C22H42O2 |
| LIPID-N-0098 | FFA(20:4) | FA | FFA | 303.23 | 304.24 | [M-H]- | C20H32O2 |
| LIPID-N-0095 | FFA(20:3) | FA | FFA | 305.25 | 306.26 | [M-H]- | C20H34O2 |
| LIPID-N-0089 | FFA(16:2) | FA | FFA | 251.20 | 252.21 | [M-H]- | C16H28O2 |
| LIPID-N-0090 | FFA(18:2) | FA | FFA | 279.23 | 280.24 | [M-H]- | C18H32O2 |
| LIPID-N-0091 | FFA(20:2) | FA | FFA | 307.26 | 308.27 | [M-H]- | C20H36O2 |
| LIPID-N-0092 | FFA(22:2) | FA | FFA | 335.30 | 336.30 | [M-H]- | C22H40O2 |
| LIPID-N-0073 | FFA(16:0) | FA | FFA | 255.23 | 256.24 | [M-H]- | C16H32O2 |
| LIPID-N-0072 | FFA(15:0) | FA | FFA | 241.22 | 242.22 | [M-H]- | C15H30O2 |
| LIPID-N-0096 | FFA(22:3) | FA | FFA | 333.28 | 334.29 | [M-H]- | C22H38O2 |
| LIPID-N-0714 | FFA(32:1) | FA | FFA | 477.47 | 478.47 | [M-H]- | C32H62O2 |
| LIPID-N-0711 | FFA(26:1) | FA | FFA | 393.37 | 394.38 | [M-H]- | C26H50O2 |
| LIPID-N-0715 | FFA(34:1) | FA | FFA | 505.50 | 506.51 | [M-H]- | C34H66O2 |
| LIPID-N-0700 | FFA(28:0) | FA | FFA | 423.42 | 424.43 | [M-H]- | C28H56O2 |
| LIPID-N-0720 | FFA(21:2) | FA | FFA | 321.28 | 322.29 | [M-H]- | C21H38O2 |
| LIPID-N-0698 | FFA(26:0) | FA | FFA | 395.39 | 396.40 | [M-H]- | C26H52O2 |
| LIPID-N-0071 | FFA(14:0) | FA | FFA | 227.20 | 228.21 | [M-H]- | C14H28O2 |
| LIPID-N-0724 | FFA(16:4) | FA | FFA | 247.17 | 248.18 | [M-H]- | C16H24O2 |
| LIPID-N-0695 | FFA(19:0) | FA | FFA | 297.28 | 298.29 | [M-H]- | C19H38O2 |
| LIPID-N-0067 | FFA(10:0) | FA | FFA | 171.14 | 172.15 | [M-H]- | C10H20O2 |
| LIPID-P-0320 | Hex2Cer(d18:1/22:0) | SP | Hex2Cer | 946.70 | 945.71 | [M+H]+ | C52H99NO13 |
| LIPID-P-2168 | HexCer(t22:1/16:1(2OH)) | SP | HexCer-AP | 606.55 | 785.60 | [M-H2O+H]+ | C44H83NO10 |
| LIPID-P-2175 | HexCer(t25:1/33:1(2OH)) | SP | HexCer-AP | 886.86 | 1065.91 | [M-H2O+H]+ | C64H123NO10 |
| LIPID-P-2180 | HexCer(t29:1/29:1(2OH)) | SP | HexCer-AP | 886.86 | 1065.91 | [M-H2O+H]+ | C64H123NO10 |
| LIPID-P-2124 | HexCer(t14:0/24:2(2OH)) | SP | HexCer-AP | 606.55 | 785.60 | [M-H2O+H]+ | C44H83NO10 |
| LIPID-P-2159 | HexCer(t18:1/20:0(2OH)) | SP | HexCer-AP | 608.56 | 787.62 | [M-H2O+H]+ | C44H85NO10 |
| LIPID-P-2156 | HexCer(t18:0/20:1(2OH)) | SP | HexCer-AP | 608.56 | 787.62 | [M-H2O+H]+ | C44H85NO10 |
| LIPID-P-2125 | HexCer(t14:1/17:0(2OH)) | SP | HexCer-AP | 510.45 | 689.51 | [M-H2O+H]+ | C37H71NO10 |
| LIPID-P-2161 | HexCer(t18:2/20:0(2OH)) | SP | HexCer-AP | 606.55 | 785.60 | [M-H2O+H]+ | C44H83NO10 |
| LIPID-P-2150 | HexCer(t17:2/40:1(2OH)) | SP | HexCer-AP | 870.83 | 1049.88 | [M-H2O+H]+ | C63H119NO10 |
| LIPID-P-2128 | HexCer(t14:1/24:0(2OH)) | SP | HexCer-AP | 608.56 | 787.62 | [M-H2O+H]+ | C44H85NO10 |
| LIPID-P-2132 | HexCer(t14:2/30:2(2OH)) | SP | HexCer-AP | 686.61 | 865.66 | [M-H2O+H]+ | C50H91NO10 |
| LIPID-P-2151 | HexCer(t17:2/42:1(2OH)) | SP | HexCer-AP | 898.86 | 1077.91 | [M-H2O+H]+ | C65H123NO10 |
| LIPID-P-2154 | HexCer(t18:0/18:0(2OH)) | SP | HexCer-AP | 582.55 | 761.60 | [M-H2O+H]+ | C42H83NO10 |
| LIPID-P-2134 | HexCer(t16:0/22:2(2OH)) | SP | HexCer-AP | 606.55 | 785.60 | [M-H2O+H]+ | C44H83NO10 |
| LIPID-P-2148 | HexCer(t17:2/32:2(2OH)) | SP | HexCer-AP | 756.69 | 935.74 | [M-H2O+H]+ | C55H101NO10 |
| LIPID-P-2149 | HexCer(t17:2/34:2(2OH)) | SP | HexCer-AP | 784.72 | 963.77 | [M-H2O+H]+ | C57H105NO10 |
| LIPID-P-0301 | HexCer(d18:1/26:0) | SP | HexCer-NS | 840.73 | 839.72 | [M+H]+ | C50H97NO8 |
| LIPID-P-0285 | HexCer(d16:1/18:0) | SP | HexCer-NS | 700.60 | 699.56 | [M+H]+ | C40H77NO8 |
| LIPID-P-0289 | HexCer(d16:1/24:1) | SP | HexCer-NS | 782.70 | 781.64 | [M+H]+ | C46H87NO8 |
| LIPID-P-0292 | HexCer(d18:1/16:0) | SP | HexCer-NS | 700.57 | 699.56 | [M+H]+ | C40H77NO8 |
| LIPID-P-0294 | HexCer(d18:1/18:0) | SP | HexCer-NS | 728.60 | 727.60 | [M+H]+ | C42H81NO8 |
| LIPID-P-0296 | HexCer(d18:1/20:0) | SP | HexCer-NS | 756.64 | 755.63 | [M+H]+ | C44H85NO8 |
| LIPID-P-2262 | HexCer(d20:1/24:0) | SP | HexCer-NS | 660.67 | 839.72 | [M-H2O+H]+ | C50H97NO8 |
| LIPID-P-2241 | HexCer(d18:2/25:0) | SP | HexCer-NS | 644.63 | 823.69 | [M-H2O+H]+ | C49H93NO8 |
| LIPID-P-2240 | HexCer(d18:2/24:2) | SP | HexCer-NS | 626.59 | 805.64 | [M-H2O+H]+ | C48H87NO8 |
| LIPID-P-0313 | HexCer(d18:2/24:1) | SP | HexCer-NS | 808.70 | 807.66 | [M+H]+ | C48H89NO8 |
| LIPID-P-0306 | HexCer(d18:1/24:1) | SP | HexCer-NS | 810.68 | 809.67 | [M+H]+ | C48H91NO8 |
| LIPID-P-0303 | HexCer(d18:1/18:1) | SP | HexCer-NS | 726.59 | 725.58 | [M+H]+ | C42H79NO8 |
| LIPID-P-0297 | HexCer(d18:1/22:0) | SP | HexCer-NS | 784.67 | 783.66 | [M+H]+ | C46H89NO8 |
| LIPID-P-0298 | HexCer(d18:1/23:0) | SP | HexCer-NS | 798.68 | 797.67 | [M+H]+ | C47H91NO8 |
| LIPID-P-0299 | HexCer(d18:1/24:0) | SP | HexCer-NS | 812.70 | 811.69 | [M+H]+ | C48H93NO8 |
| LIPID-P-0300 | HexCer(d18:1/25:0) | SP | HexCer-NS | 826.71 | 825.71 | [M+H]+ | C49H95NO8 |
| LIPID-P-2237 | HexCer(d18:2/22:1) | SP | HexCer-NS | 600.57 | 779.63 | [M-H2O+H]+ | C46H85NO8 |
| LIPID-P-0305 | HexCer(d18:1/22:1) | SP | HexCer-NS | 782.65 | 781.64 | [M+H]+ | C46H87NO8 |
| LIPID-N-0969 | LNAPE(20:5/N-18:1) | GP | LNAPE | 762.51 | 763.52 | [M-H]- | C43H74NO8P |
| LIPID-N-0975 | LNAPE(22:6/N-18:2) | GP | LNAPE | 786.51 | 787.52 | [M-H]- | C45H74NO8P |
| LIPID-N-0973 | LNAPE(22:6/N-20:1) | GP | LNAPE | 816.55 | 817.56 | [M-H]- | C47H80NO8P |
| LIPID-N-0957 | LNAPE(16:0/N-16:0) | GP | LNAPE | 690.51 | 691.52 | [M-H]- | C37H74NO8P |
| LIPID-N-0958 | LNAPE(16:1/N-16:0) | GP | LNAPE | 688.49 | 689.50 | [M-H]- | C37H72NO8P |
| LIPID-N-0959 | LNAPE(16:0/N-18:1) | GP | LNAPE | 716.52 | 717.53 | [M-H]- | C39H76NO8P |
| LIPID-N-0963 | LNAPE(22:1/N-18:1) | GP | LNAPE | 798.60 | 799.61 | [M-H]- | C45H86NO8P |
| LIPID-N-0964 | LNAPE(18:1/N-18:2) | GP | LNAPE | 740.52 | 741.53 | [M-H]- | C41H76NO8P |
| LIPID-N-0968 | LNAPE(20:4/N-18:2) | GP | LNAPE | 762.51 | 763.52 | [M-H]- | C43H74NO8P |
| LIPID-N-0965 | LNAPE(18:2/N-18:2) | GP | LNAPE | 738.51 | 739.52 | [M-H]- | C41H74NO8P |
| LIPID-N-0659 | LPA(22:5) | GP | LPA | 483.24 | 482.24 | [M-H]- | C25H39O7P |
| LIPID-N-0619 | LPA(20:4) | GP | LPA | 457.24 | 458.24 | [M-H]- | C23H39O7P |
| LIPID-N-0618 | LPA(18:2) | GP | LPA | 433.24 | 434.24 | [M-H]- | C21H39O7P |
| LIPID-N-0616 | LPA(18:0) | GP | LPA | 437.27 | 438.27 | [M-H]- | C21H43O7P |
| LIPID-N-0615 | LPA(16:0) | GP | LPA | 409.24 | 410.24 | [M-H]- | C19H39O7P |
| LIPID-P-0388 | LPC(22:4/0:0) | GP | LPC | 572.37 | 571.36 | [M+H]+ | C30H54NO7P |
| LIPID-P-0386 | LPC(20:4) | GP | LPC | 544.34 | 543.33 | [M+H]+ | C28H50NO7P |
| LIPID-P-0392 | LPC(22:6) | GP | LPC | 568.34 | 567.33 | [M+H]+ | C30H50NO7P |
| LIPID-P-0391 | LPC(22:5/0:0) | GP | LPC | 570.36 | 569.35 | [M+H]+ | C30H52NO7P |
| LIPID-P-0387 | LPC(0:0/22:4) | GP | LPC | 572.37 | 571.36 | [M+H]+ | C30H54NO7P |
| LIPID-P-0384 | LPC(20:3) | GP | LPC | 546.36 | 545.35 | [M+H]+ | C28H52NO7P |
| LIPID-P-0351 | LPC(0:0/15:0) | GP | LPC | 482.32 | 481.32 | [M+H]+ | C23H48NO7P |
| LIPID-P-0350 | LPC(14:0) | GP | LPC | 468.31 | 467.30 | [M+H]+ | C22H46NO7P |
| LIPID-P-1929 | LPC(16:2) | GP | LPC | 492.31 | 491.30 | [M+H]+ | C24H46NO7P |
| LIPID-P-0381 | LPC(20:2/0:0) | GP | LPC | 548.37 | 547.36 | [M+H]+ | C28H54NO7P |
| LIPID-P-0380 | LPC(0:0/20:2) | GP | LPC | 548.37 | 547.36 | [M+H]+ | C28H54NO7P |
| LIPID-P-0379 | LPC(18:2/0:0) | GP | LPC | 520.34 | 519.33 | [M+H]+ | C26H50NO7P |
| LIPID-P-0378 | LPC(0:0/18:2) | GP | LPC | 520.34 | 519.33 | [M+H]+ | C26H50NO7P |
| LIPID-P-0377 | LPC(24:1) | GP | LPC | 606.45 | 605.44 | [M+H]+ | C32H64NO7P |
| LIPID-P-0376 | LPC(22:1/0:0) | GP | LPC | 578.42 | 577.41 | [M+H]+ | C30H60NO7P |
| LIPID-P-0383 | LPC(18:3) | GP | LPC | 518.32 | 517.32 | [M+H]+ | C26H48NO7P |
| LIPID-P-0374 | LPC(20:1/0:0) | GP | LPC | 550.39 | 549.38 | [M+H]+ | C28H56NO7P |
| LIPID-P-0373 | LPC(0:0/20:1) | GP | LPC | 550.39 | 549.38 | [M+H]+ | C28H56NO7P |
| LIPID-P-0372 | LPC(19:1) | GP | LPC | 536.37 | 535.36 | [M+H]+ | C27H54NO7P |
| LIPID-P-0371 | LPC(18:1/0:0) | GP | LPC | 522.36 | 521.35 | [M+H]+ | C26H52NO7P |
| LIPID-P-0370 | LPC(0:0/18:1) | GP | LPC | 522.36 | 521.35 | [M+H]+ | C26H52NO7P |
| LIPID-P-0375 | LPC(0:0/22:1) | GP | LPC | 578.42 | 577.41 | [M+H]+ | C30H60NO7P |
| LIPID-P-0368 | LPC(16:1) | GP | LPC | 494.32 | 493.32 | [M+H]+ | C24H48NO7P |
| LIPID-P-0390 | LPC(0:0/22:5) | GP | LPC | 570.36 | 569.35 | [M+H]+ | C30H52NO7P |
| LIPID-P-0352 | LPC(15:0/0:0) | GP | LPC | 482.32 | 481.32 | [M+H]+ | C23H48NO7P |
| LIPID-P-0369 | LPC(17:1) | GP | LPC | 508.34 | 507.33 | [M+H]+ | C25H50NO7P |
| LIPID-P-0353 | LPC(0:0/16:0) | GP | LPC | 496.34 | 495.33 | [M+H]+ | C24H50NO7P |
| LIPID-P-0354 | LPC(16:0/0:0) | GP | LPC | 496.34 | 495.33 | [M+H]+ | C24H50NO7P |
| LIPID-P-0355 | LPC(0:0/17:0) | GP | LPC | 510.36 | 509.35 | [M+H]+ | C25H52NO7P |
| LIPID-P-0356 | LPC(17:0/0:0) | GP | LPC | 510.36 | 509.35 | [M+H]+ | C25H52NO7P |
| LIPID-P-0389 | LPC(20:5) | GP | LPC | 542.32 | 541.32 | [M+H]+ | C28H48NO7P |
| LIPID-P-0359 | LPC(0:0/19:0) | GP | LPC | 538.39 | 537.38 | [M+H]+ | C27H56NO7P |
| LIPID-P-0360 | LPC(19:0/0:0) | GP | LPC | 538.39 | 537.38 | [M+H]+ | C27H56NO7P |
| LIPID-P-0361 | LPC(0:0/20:0) | GP | LPC | 552.40 | 551.40 | [M+H]+ | C28H58NO7P |
| LIPID-P-0362 | LPC(20:0/0:0) | GP | LPC | 552.40 | 551.40 | [M+H]+ | C28H58NO7P |
| LIPID-P-0366 | LPC(14:1) | GP | LPC | 466.29 | 465.29 | [M+H]+ | C22H44NO7P |
| LIPID-P-0363 | LPC(22:0) | GP | LPC | 580.43 | 579.43 | [M+H]+ | C30H62NO7P |
| LIPID-P-0358 | LPC(18:0/0:0) | GP | LPC | 524.37 | 523.36 | [M+H]+ | C26H54NO7P |
| LIPID-P-0393 | LPC(O-14:0) | GP | LPC-O | 454.33 | 453.32 | [M+H]+ | C22H48NO6P |
| LIPID-P-0394 | LPC(O-16:0) | GP | LPC-O | 482.36 | 481.35 | [M+H]+ | C24H52NO6P |
| LIPID-P-0395 | LPC(O-18:0) | GP | LPC-O | 510.39 | 509.38 | [M+H]+ | C26H56NO6P |
| LIPID-P-0398 | LPC(O-14:1) | GP | LPC-O | 452.31 | 451.31 | [M+H]+ | C22H46NO6P |
| LIPID-P-0399 | LPC(O-16:1) | GP | LPC-O | 480.35 | 479.34 | [M+H]+ | C24H50NO6P |
| LIPID-P-0400 | LPC(O-18:1) | GP | LPC-O | 508.38 | 507.37 | [M+H]+ | C26H54NO6P |
| LIPID-P-0401 | LPC(O-20:1) | GP | LPC-O | 536.41 | 535.40 | [M+H]+ | C28H58NO6P |
| LIPID-P-0403 | LPC(O-18:2) | GP | LPC-O | 506.36 | 505.35 | [M+H]+ | C26H52NO6P |
| LIPID-P-0396 | LPC(O-20:0) | GP | LPC-O | 538.42 | 537.42 | [M+H]+ | C28H60NO6P |
| LIPID-P-0406 | LPC(O-20:3) | GP | LPC-O | 532.38 | 531.37 | [M+H]+ | C28H54NO6P |
| LIPID-P-0411 | LPE(18:0/0:0) | GP | LPE | 482.32 | 481.32 | [M+H]+ | C23H48NO7P |
| LIPID-P-0408 | LPE(16:0/0:0) | GP | LPE | 454.29 | 453.29 | [M+H]+ | C21H44NO7P |
| LIPID-P-0407 | LPE(0:0/16:0) | GP | LPE | 454.29 | 453.29 | [M+H]+ | C21H44NO7P |
| LIPID-P-0437 | LPE(22:6) | GP | LPE | 526.29 | 525.29 | [M+H]+ | C27H44NO7P |
| LIPID-P-0436 | LPE(22:5/0:0) | GP | LPE | 528.31 | 527.30 | [M+H]+ | C27H46NO7P |
| LIPID-P-0435 | LPE(0:0/22:5) | GP | LPE | 528.31 | 527.30 | [M+H]+ | C27H46NO7P |
| LIPID-P-0434 | LPE(20:5) | GP | LPE | 500.28 | 499.27 | [M+H]+ | C25H42NO7P |
| LIPID-P-0432 | LPE(20:4) | GP | LPE | 502.29 | 501.29 | [M+H]+ | C25H44NO7P |
| LIPID-P-0431 | LPE(22:3) | GP | LPE | 532.34 | 531.33 | [M+H]+ | C27H50NO7P |
| LIPID-P-0429 | LPE(0:0/20:3) | GP | LPE | 504.31 | 503.30 | [M+H]+ | C25H46NO7P |
| LIPID-P-0428 | LPE(18:3) | GP | LPE | 476.28 | 475.27 | [M+H]+ | C23H42NO7P |
| LIPID-P-0426 | LPE(20:2/0:0) | GP | LPE | 506.32 | 505.32 | [M+H]+ | C25H48NO7P |
| LIPID-P-0433 | LPE(22:4) | GP | LPE | 530.32 | 529.32 | [M+H]+ | C27H48NO7P |
| LIPID-P-0423 | LPE(0:0/18:2) | GP | LPE | 478.29 | 477.29 | [M+H]+ | C23H44NO7P |
| LIPID-P-0419 | LPE(0:0/20:1) | GP | LPE | 508.34 | 507.33 | [M+H]+ | C25H50NO7P |
| LIPID-P-0418 | LPE(18:1/0:0) | GP | LPE | 480.31 | 479.30 | [M+H]+ | C23H46NO7P |
| LIPID-P-0409 | LPE(17:0) | GP | LPE | 468.30 | 467.30 | [M+H]+ | C22H46NO7P |
| LIPID-P-0424 | LPE(18:2/0:0) | GP | LPE | 478.29 | 477.29 | [M+H]+ | C23H44NO7P |
| LIPID-P-0417 | LPE(0:0/18:1) | GP | LPE | 480.31 | 479.30 | [M+H]+ | C23H46NO7P |
| LIPID-P-0415 | LPE(16:1) | GP | LPE | 452.28 | 451.27 | [M+H]+ | C21H42NO7P |
| LIPID-P-0410 | LPE(0:0/18:0) | GP | LPE | 482.32 | 481.32 | [M+H]+ | C23H48NO7P |
| LIPID-P-0445 | LPE(P-18:1) | GP | LPE-P | 464.30 | 463.31 | [M+H]+ | C23H46NO6P |
| LIPID-P-0439 | LPE(P-17:0) | GP | LPE-P | 452.31 | 451.31 | [M+H]+ | C22H46NO6P |
| LIPID-P-0440 | LPE(P-18:0) | GP | LPE-P | 466.30 | 465.32 | [M+H]+ | C23H48NO6P |
| LIPID-P-0438 | LPE(P-16:0) | GP | LPE-P | 438.30 | 437.29 | [M+H]+ | C21H44NO6P |
| LIPID-N-0668 | LPG(22:4) | GP | LPG | 559.30 | 560.31 | [M-H]- | C28H49O9P |
| LIPID-N-0167 | LPG(18:0) | GP | LPG | 511.30 | 512.31 | [M-H]- | C24H49O9P |
| LIPID-N-0665 | LPG(20:2) | GP | LPG | 535.30 | 536.31 | [M-H]- | C26H49O9P |
| LIPID-N-0666 | LPG(20:3) | GP | LPG | 533.29 | 534.30 | [M-H]- | C26H47O9P |
| LIPID-N-0660 | LPG(15:0) | GP | LPG | 469.26 | 470.26 | [M-H]- | C21H43O9P |
| LIPID-N-0172 | LPG(20:4) | GP | LPG | 531.27 | 532.28 | [M-H]- | C26H45O9P |
| LIPID-N-0171 | LPG(18:2) | GP | LPG | 507.27 | 508.28 | [M-H]- | C24H45O9P |
| LIPID-N-0170 | LPG(18:1) | GP | LPG | 509.29 | 510.30 | [M-H]- | C24H47O9P |
| LIPID-N-0169 | LPG(16:1) | GP | LPG | 481.26 | 482.26 | [M-H]- | C22H43O9P |
| LIPID-N-0166 | LPG(16:0) | GP | LPG | 483.27 | 484.28 | [M-H]- | C22H45O9P |
| LIPID-N-0174 | LPG(22:6) | GP | LPG | 557.29 | 556.28 | [M-H]- | C28H45O9P |
| LIPID-N-0179 | LPI(18:2) | GP | LPI | 595.29 | 596.30 | [M-H]- | C27H49O12P |
| LIPID-N-0178 | LPI(18:1) | GP | LPI | 597.30 | 598.31 | [M-H]- | C27H51O12P |
| LIPID-N-0679 | LPI(14:0) | GP | LPI | 543.26 | 544.26 | [M-H]- | C23H45O12P |
| LIPID-N-0177 | LPI(16:1) | GP | LPI | 569.27 | 570.28 | [M-H]- | C25H47O12P |
| LIPID-N-0180 | LPI(20:3) | GP | LPI | 621.30 | 622.31 | [M-H]- | C29H51O12P |
| LIPID-N-0175 | LPI(16:0) | GP | LPI | 571.29 | 572.30 | [M-H]- | C25H49O12P |
| LIPID-N-0176 | LPI(18:0) | GP | LPI | 599.32 | 600.33 | [M-H]- | C27H53O12P |
| LIPID-N-0185 | LPI(22:6) | GP | LPI | 643.29 | 644.30 | [M-H]- | C31H49O12P |
| LIPID-N-0182 | LPI(22:4) | GP | LPI | 647.32 | 648.33 | [M-H]- | C31H53O12P |
| LIPID-N-0181 | LPI(20:4) | GP | LPI | 619.29 | 620.30 | [M-H]- | C29H49O12P |
| LIPID-N-0196 | LPS(18:2) | GP | LPS | 520.27 | 521.28 | [M-H]- | C24H44NO9P |
| LIPID-N-0186 | LPS(16:0) | GP | LPS | 496.27 | 497.28 | [M-H]- | C22H44NO9P |
| LIPID-N-0197 | LPS(20:3) | GP | LPS | 546.28 | 547.29 | [M-H]- | C26H46NO9P |
| LIPID-N-0199 | LPS(20:5) | GP | LPS | 542.25 | 543.26 | [M-H]- | C26H42NO9P |
| LIPID-N-0201 | LPS(22:6) | GP | LPS | 568.27 | 569.28 | [M-H]- | C28H44NO9P |
| LIPID-N-0192 | LPS(18:1) | GP | LPS | 522.28 | 523.29 | [M-H]- | C24H46NO9P |
| LIPID-N-0198 | LPS(20:4) | GP | LPS | 544.27 | 545.28 | [M-H]- | C26H44NO9P |
| LIPID-P-0276 | MG(18:0) | GL | MG | 376.35 | 358.31 | [M+NH4]+ | C21H42O4 |
| LIPID-P-0274 | MG(16:0) | GL | MG | 348.32 | 330.28 | [M+NH4]+ | C19H38O4 |
| LIPID-N-1486 | PA(20:0_20:4) | GP | PA | 751.53 | 752.54 | [M-H]- | C43H77O8P |
| LIPID-N-1449 | PA(18:0_21:0) | GP | PA | 745.57 | 746.58 | [M-H]- | C42H83O8P |
| LIPID-N-1450 | PA(18:0_23:0) | GP | PA | 773.61 | 774.61 | [M-H]- | C44H87O8P |
| LIPID-N-1457 | PA(20:0_18:1) | GP | PA | 729.54 | 730.55 | [M-H]- | C41H79O8P |
| LIPID-N-1462 | PA(16:0_20:2) | GP | PA | 699.50 | 700.50 | [M-H]- | C39H73O8P |
| LIPID-N-1465 | PA(20:0_18:2) | GP | PA | 727.53 | 728.54 | [M-H]- | C41H77O8P |
| LIPID-N-1472 | PA(20:1_18:2) | GP | PA | 725.51 | 726.52 | [M-H]- | C41H75O8P |
| LIPID-N-1480 | PA(18:1_16:3) | GP | PA | 667.43 | 668.44 | [M-H]- | C37H65O8P |
| LIPID-N-0635 | PA(18:1_18:1) | GP | PA | 699.50 | 700.50 | [M-H]- | C39H73O8P |
| LIPID-N-0640 | PA(18:0_20:4) | GP | PA | 723.50 | 724.50 | [M-H]- | C41H73O8P |
| LIPID-N-0688 | PA(22:0_18:1) | GP | PA | 757.57 | 758.58 | [M-H]- | C43H83O8P |
| LIPID-N-0647 | PA(18:0_22:6) | GP | PA | 747.50 | 748.50 | [M-H]- | C43H73O8P |
| LIPID-N-0692 | PA(18:1_18:2) | GP | PA | 697.48 | 698.49 | [M-H]- | C39H71O8P |
| LIPID-N-1513 | PA(20:4_22:5) | GP | PA | 769.48 | 770.49 | [M-H]- | C45H71O8P |
| LIPID-N-1511 | PA(20:2_22:5) | GP | PA | 773.51 | 774.52 | [M-H]- | C45H75O8P |
| LIPID-N-1484 | PA(18:0_22:4) | GP | PA | 751.53 | 752.54 | [M-H]- | C43H77O8P |
| LIPID-N-1510 | PA(18:2_22:5) | GP | PA | 745.48 | 746.49 | [M-H]- | C43H71O8P |
| LIPID-N-1504 | PA(20:2_20:4) | GP | PA | 747.50 | 748.50 | [M-H]- | C43H73O8P |
| LIPID-N-1500 | PA(16:1_22:5) | GP | PA | 719.47 | 720.47 | [M-H]- | C41H69O8P |
| LIPID-N-1494 | PA(20:1_20:4) | GP | PA | 749.51 | 750.52 | [M-H]- | C43H75O8P |
| LIPID-N-1492 | PA(16:0_22:5) | GP | PA | 721.48 | 722.49 | [M-H]- | C41H71O8P |
| LIPID-N-1489 | PA(20:0_22:4) | GP | PA | 779.56 | 780.57 | [M-H]- | C45H81O8P |
| LIPID-N-0218 | PC(16:0_18:1) | GP | PC | 804.58 | 759.58 | [M+COOH]- | C42H82NO8P |
| LIPID-N-0208 | PC(16:0_18:0) | GP | PC | 806.59 | 761.59 | [M+COOH]- | C42H84NO8P |
| LIPID-N-0984 | PC(13:0_16:0) | GP | PC | 736.51 | 691.52 | [M+COOH]- | C37H74NO8P |
| LIPID-N-0206 | PC(16:0_16:0) | GP | PC | 778.56 | 733.56 | [M+COOH]- | C40H80NO8P |
| LIPID-N-0205 | PC(15:0_16:0) | GP | PC | 764.54 | 719.55 | [M+COOH]- | C39H78NO8P |
| LIPID-N-0203 | PC(16:0_14:0) | GP | PC | 750.53 | 705.53 | [M+COOH]- | C38H76NO8P |
| LIPID-N-0220 | PC(18:0_18:1) | GP | PC | 832.61 | 787.61 | [M+COOH]- | C44H86NO8P |
| LIPID-N-0228 | PC(16:1_16:1) | GP | PC | 774.53 | 729.53 | [M+COOH]- | C40H76NO8P |
| LIPID-N-0231 | PC(16:1_18:1) | GP | PC | 802.56 | 757.56 | [M+COOH]- | C42H80NO8P |
| LIPID-N-1042 | PC(15:1_22:4) | GP | PC | 838.56 | 793.56 | [M+COOH]- | C45H80NO8P |
| LIPID-N-1041 | PC(18:2_19:2) | GP | PC | 840.58 | 795.58 | [M+COOH]- | C45H82NO8P |
| LIPID-N-1033 | PC(18:1_17:2) | GP | PC | 814.56 | 769.56 | [M+COOH]- | C43H80NO8P |
| LIPID-N-1016 | PC(18:0_15:1) | GP | PC | 790.56 | 745.56 | [M+COOH]- | C41H80NO8P |
| LIPID-N-1014 | PC(16:0_17:1) | GP | PC | 790.56 | 745.56 | [M+COOH]- | C41H80NO8P |
| LIPID-N-1002 | PC(10:0_22:0) | GP | PC | 778.56 | 733.56 | [M+COOH]- | C40H80NO8P |
| LIPID-N-1001 | PC(13:1_18:2) | GP | PC | 758.50 | 713.50 | [M+COOH]- | C39H72NO8P |
| LIPID-N-1000 | PC(15:1_16:1) | GP | PC | 760.51 | 715.52 | [M+COOH]- | C39H74NO8P |
| LIPID-N-0996 | PC(13:0_18:1) | GP | PC | 762.53 | 717.53 | [M+COOH]- | C39H76NO8P |
| LIPID-N-0994 | PC(13:0_18:0) | GP | PC | 764.54 | 719.55 | [M+COOH]- | C39H78NO8P |
| LIPID-N-0989 | PC(12:0_18:1) | GP | PC | 748.51 | 703.52 | [M+COOH]- | C38H74NO8P |
| LIPID-N-1044 | PC(15:0_22:6) | GP | PC | 836.54 | 791.55 | [M+COOH]- | C45H78NO8P |
| LIPID-N-0980 | PC(10:0_18:0) | GP | PC | 722.50 | 677.50 | [M+COOH]- | C36H72NO8P |
| LIPID-N-0304 | PC(18:2_20:4) | GP | PC | 850.56 | 805.56 | [M+COOH]- | C46H80NO8P |
| LIPID-N-0232 | PC(16:0_18:2) | GP | PC | 802.56 | 757.56 | [M+COOH]- | C42H80NO8P |
| LIPID-N-0235 | PC(18:1_18:1) | GP | PC | 830.59 | 785.59 | [M+COOH]- | C44H84NO8P |
| LIPID-N-0236 | PC(18:0_18:2) | GP | PC | 830.59 | 785.59 | [M+COOH]- | C44H84NO8P |
| LIPID-N-0237 | PC(19:0_18:2) | GP | PC | 844.61 | 799.61 | [M+COOH]- | C45H86NO8P |
| LIPID-N-0250 | PC(16:1_18:2) | GP | PC | 800.54 | 755.55 | [M+COOH]- | C42H78NO8P |
| LIPID-N-0254 | PC(18:1_18:2) | GP | PC | 828.58 | 783.58 | [M+COOH]- | C44H82NO8P |
| LIPID-N-0269 | PC(15:0_20:4) | GP | PC | 812.54 | 767.55 | [M+COOH]- | C43H78NO8P |
| LIPID-N-0270 | PC(18:2_18:2) | GP | PC | 826.56 | 781.56 | [M+COOH]- | C44H80NO8P |
| LIPID-N-0272 | PC(16:0_20:4) | GP | PC | 826.56 | 781.56 | [M+COOH]- | C44H80NO8P |
| LIPID-N-0275 | PC(18:0_20:4) | GP | PC | 854.59 | 809.59 | [M+COOH]- | C46H84NO8P |
| LIPID-N-0277 | PC(19:0_20:4) | GP | PC | 868.61 | 823.61 | [M+COOH]- | C47H86NO8P |
| LIPID-N-0303 | PC(16:0_22:6) | GP | PC | 850.56 | 805.56 | [M+COOH]- | C46H80NO8P |
| LIPID-N-1010 | PC(14:1_18:1) | GP | PC | 774.53 | 729.53 | [M+COOH]- | C40H76NO8P |
| LIPID-P-0569 | PC(O-18:1_22:6) | GP | PC-O | 818.61 | 817.60 | [M+H]+ | C48H84NO7P |
| LIPID-P-0538 | PC(O-18:1_16:0) | GP | PC-O | 746.61 | 745.60 | [M+H]+ | C42H84NO7P |
| LIPID-P-0546 | PC(O-18:1_20:1) | GP | PC-O | 800.65 | 799.65 | [M+H]+ | C46H90NO7P |
| LIPID-P-0536 | PC(O-16:0_14:1) | GP | PC-O | 690.54 | 689.54 | [M+H]+ | C38H76NO7P |
| LIPID-P-0535 | PC(O-16:0_22:0) | GP | PC-O | 804.68 | 803.68 | [M+H]+ | C46H94NO7P |
| LIPID-P-0532 | PC(O-16:0_16:0) | GP | PC-O | 720.59 | 719.58 | [M+H]+ | C40H82NO7P |
| LIPID-P-0531 | PC(O-16:0_14:0) | GP | PC-O | 692.56 | 691.55 | [M+H]+ | C38H78NO7P |
| LIPID-P-0549 | PC(O-16:1_18:2) | GP | PC-O | 742.58 | 741.57 | [M+H]+ | C42H80NO7P |
| LIPID-P-0553 | PC(O-14:0_20:4) | GP | PC-O | 740.56 | 739.55 | [M+H]+ | C42H78NO7P |
| LIPID-P-0554 | PC(O-16:0_20:4) | GP | PC-O | 768.59 | 767.58 | [M+H]+ | C44H82NO7P |
| LIPID-P-0555 | PC(O-18:0_20:4) | GP | PC-O | 796.62 | 795.61 | [M+H]+ | C46H86NO7P |
| LIPID-P-0541 | PC(O-18:1_22:0) | GP | PC-O | 830.70 | 829.69 | [M+H]+ | C48H96NO7P |
| LIPID-P-0559 | PC(O-16:1_20:4) | GP | PC-O | 766.58 | 765.57 | [M+H]+ | C44H80NO7P |
| LIPID-P-0562 | PC(O-22:1_20:4) | GP | PC-O | 850.67 | 849.66 | [M+H]+ | C50H92NO7P |
| LIPID-P-0565 | PC(O-18:2_20:4) | GP | PC-O | 792.59 | 791.58 | [M+H]+ | C46H82NO7P |
| LIPID-P-0568 | PC(O-16:1_22:6) | GP | PC-O | 790.58 | 789.57 | [M+H]+ | C46H80NO7P |
| LIPID-P-0560 | PC(O-18:1_20:4) | GP | PC-O | 794.61 | 793.60 | [M+H]+ | C46H84NO7P |
| LIPID-P-0544 | PC(O-16:0_18:2) | GP | PC-O | 744.59 | 743.58 | [M+H]+ | C42H82NO7P |
| LIPID-N-0411 | PE(15:0_22:6) | GP | PE | 748.49 | 749.50 | [M-H]- | C42H72NO8P |
| LIPID-N-0412 | PE(20:5_18:1) | GP | PE | 762.51 | 763.52 | [M-H]- | C43H74NO8P |
| LIPID-N-0413 | PE(22:6_16:0) | GP | PE | 762.51 | 763.52 | [M-H]- | C43H74NO8P |
| LIPID-N-0414 | PE(17:0_22:6) | GP | PE | 776.52 | 777.53 | [M-H]- | C44H76NO8P |
| LIPID-N-0415 | PE(22:6_18:0) | GP | PE | 790.54 | 791.55 | [M-H]- | C45H78NO8P |
| LIPID-N-0416 | PE(18:1_22:5) | GP | PE | 790.54 | 791.55 | [M-H]- | C45H78NO8P |
| LIPID-N-0396 | PE(16:1_20:4) | GP | PE | 736.49 | 737.50 | [M-H]- | C41H72NO8P |
| LIPID-N-0403 | PE(18:0_22:5) | GP | PE | 792.55 | 793.56 | [M-H]- | C45H80NO8P |
| LIPID-N-0368 | PE(18:1_18:2) | GP | PE | 740.52 | 741.53 | [M-H]- | C41H76NO8P |
| LIPID-N-0372 | PE(20:3_18:0) | GP | PE | 768.55 | 769.56 | [M-H]- | C43H80NO8P |
| LIPID-N-0373 | PE(20:1_18:2) | GP | PE | 768.55 | 769.56 | [M-H]- | C43H80NO8P |
| LIPID-N-0382 | PE(18:2_18:2) | GP | PE | 738.51 | 739.52 | [M-H]- | C41H74NO8P |
| LIPID-N-0383 | PE(16:0_20:4) | GP | PE | 738.51 | 739.52 | [M-H]- | C41H74NO8P |
| LIPID-N-0384 | PE(18:3_18:1) | GP | PE | 738.51 | 739.52 | [M-H]- | C41H74NO8P |
| LIPID-N-0422 | PE(16:1_22:6) | GP | PE | 760.49 | 761.50 | [M-H]- | C43H72NO8P |
| LIPID-N-0386 | PE(17:0_20:4) | GP | PE | 752.52 | 753.53 | [M-H]- | C42H76NO8P |
| LIPID-N-0389 | PE(16:0_22:4) | GP | PE | 766.54 | 767.55 | [M-H]- | C43H78NO8P |
| LIPID-N-0392 | PE(18:0_22:4) | GP | PE | 794.57 | 795.58 | [M-H]- | C45H82NO8P |
| LIPID-N-1107 | PE(20:4_22:6) | GP | PE | 810.51 | 811.52 | [M-H]- | C47H74NO8P |
| LIPID-N-0399 | PE(18:1_20:4) | GP | PE | 764.52 | 765.53 | [M-H]- | C43H76NO8P |
| LIPID-N-0400 | PE(18:0_20:5) | GP | PE | 764.52 | 765.53 | [M-H]- | C43H76NO8P |
| LIPID-N-0401 | PE(16:0_22:5) | GP | PE | 764.52 | 765.53 | [M-H]- | C43H76NO8P |
| LIPID-N-0387 | PE(20:4_18:0) | GP | PE | 766.54 | 767.55 | [M-H]- | C43H78NO8P |
| LIPID-N-0426 | PE(22:6_18:1) | GP | PE | 788.52 | 789.53 | [M-H]- | C45H76NO8P |
| LIPID-N-0421 | PE(20:4_22:2) | GP | PE | 818.57 | 819.58 | [M-H]- | C47H82NO8P |
| LIPID-N-0332 | PE(14:0_18:1) | GP | PE | 688.49 | 689.50 | [M-H]- | C37H72NO8P |
| LIPID-N-0323 | PE(16:0_16:0) | GP | PE | 690.51 | 691.52 | [M-H]- | C37H74NO8P |
| LIPID-N-0327 | PE(18:0_18:0) | GP | PE | 746.57 | 747.58 | [M-H]- | C41H82NO8P |
| LIPID-N-0328 | PE(20:0_18:0) | GP | PE | 774.60 | 775.61 | [M-H]- | C43H86NO8P |
| LIPID-N-0331 | PE(16:1_16:0) | GP | PE | 688.49 | 689.50 | [M-H]- | C37H72NO8P |
| LIPID-N-0334 | PE(16:0_18:1) | GP | PE | 716.52 | 717.53 | [M-H]- | C39H76NO8P |
| LIPID-N-0335 | PE(16:1_18:0) | GP | PE | 716.52 | 717.53 | [M-H]- | C39H76NO8P |
| LIPID-N-0337 | PE(20:1_16:0) | GP | PE | 744.55 | 745.56 | [M-H]- | C41H80NO8P |
| LIPID-N-0338 | PE(18:1_18:0) | GP | PE | 744.55 | 745.56 | [M-H]- | C41H80NO8P |
| LIPID-N-0339 | PE(20:1_18:0) | GP | PE | 772.59 | 773.59 | [M-H]- | C43H84NO8P |
| LIPID-N-0349 | PE(18:1_16:1) | GP | PE | 714.51 | 715.52 | [M-H]- | C39H74NO8P |
| LIPID-N-0350 | PE(18:2_16:0) | GP | PE | 714.51 | 715.52 | [M-H]- | C39H74NO8P |
| LIPID-N-0352 | PE(18:1_18:1) | GP | PE | 742.54 | 743.55 | [M-H]- | C41H78NO8P |
| LIPID-N-0353 | PE(18:0_18:2) | GP | PE | 742.54 | 743.55 | [M-H]- | C41H78NO8P |
| LIPID-N-0357 | PE(20:0_18:2) | GP | PE | 770.57 | 771.58 | [M-H]- | C43H82NO8P |
| LIPID-N-1105 | PE(20:2_22:6) | GP | PE | 814.54 | 815.55 | [M-H]- | C47H78NO8P |
| LIPID-N-0358 | PE(20:1_18:1) | GP | PE | 770.57 | 771.58 | [M-H]- | C43H82NO8P |
| LIPID-N-0362 | PE(18:1_22:1) | GP | PE | 798.60 | 799.61 | [M-H]- | C45H86NO8P |
| LIPID-N-0340 | PE(20:0_18:1) | GP | PE | 772.59 | 773.59 | [M-H]- | C43H84NO8P |
| LIPID-N-0365 | PE(18:3_16:0) | GP | PE | 712.49 | 713.50 | [M-H]- | C39H72NO8P |
| LIPID-N-1102 | PE(22:3_20:4) | GP | PE | 816.55 | 817.56 | [M-H]- | C47H80NO8P |
| LIPID-N-1096 | PE(20:2_20:4) | GP | PE | 790.54 | 791.55 | [M-H]- | C45H78NO8P |
| LIPID-N-1099 | PE(20:2_20:5) | GP | PE | 788.52 | 789.53 | [M-H]- | C45H76NO8P |
| LIPID-N-1063 | PE(17:0_18:2) | GP | PE | 728.52 | 729.53 | [M-H]- | C40H76NO8P |
| LIPID-N-1079 | PE(15:0_22:4) | GP | PE | 752.52 | 753.53 | [M-H]- | C42H76NO8P |
| LIPID-N-1086 | PE(15:1_22:4) | GP | PE | 750.51 | 751.52 | [M-H]- | C42H74NO8P |
| LIPID-N-1088 | PE(16:1_22:4) | GP | PE | 764.52 | 765.53 | [M-H]- | C43H76NO8P |
| LIPID-N-1092 | PE(20:1_20:4) | GP | PE | 792.55 | 793.56 | [M-H]- | C45H80NO8P |
| LIPID-N-0366 | PE(16:1_18:2) | GP | PE | 712.49 | 713.50 | [M-H]- | C39H72NO8P |
| LIPID-N-0845 | PE(O-20:1_22:5) | GP | PE-O | 804.59 | 805.60 | [M-H]- | C47H84NO7P |
| LIPID-N-0840 | PE(O-16:1_22:5) | GP | PE-O | 748.53 | 749.54 | [M-H]- | C43H76NO7P |
| LIPID-N-0837 | PE(O-18:2_20:4) | GP | PE-O | 748.53 | 749.54 | [M-H]- | C43H76NO7P |
| LIPID-N-0831 | PE(O-16:1_20:5) | GP | PE-O | 720.50 | 721.50 | [M-H]- | C41H72NO7P |
| LIPID-N-0828 | PE(O-18:1_24:4) | GP | PE-O | 806.61 | 807.61 | [M-H]- | C47H86NO7P |
| LIPID-N-0825 | PE(O-22:1_20:4) | GP | PE-O | 806.61 | 807.61 | [M-H]- | C47H86NO7P |
| LIPID-N-0857 | PE(O-16:1_22:6) | GP | PE-O | 746.51 | 747.52 | [M-H]- | C43H74NO7P |
| LIPID-N-0871 | PE(O-18:2_22:6) | GP | PE-O | 772.53 | 773.54 | [M-H]- | C45H76NO7P |
| LIPID-N-0865 | PE(O-20:1_22:6) | GP | PE-O | 802.58 | 803.58 | [M-H]- | C47H82NO7P |
| LIPID-N-0862 | PE(O-18:3_22:4) | GP | PE-O | 774.54 | 775.55 | [M-H]- | C45H78NO7P |
| LIPID-N-0859 | PE(O-18:1_22:6) | GP | PE-O | 774.54 | 775.55 | [M-H]- | C45H78NO7P |
| LIPID-N-0846 | PE(O-20:0_22:6) | GP | PE-O | 804.59 | 805.60 | [M-H]- | C47H84NO7P |
| LIPID-N-0847 | PE(O-18:1_24:5) | GP | PE-O | 804.59 | 805.60 | [M-H]- | C47H84NO7P |
| LIPID-N-0855 | PE(O-18:3_20:4) | GP | PE-O | 746.51 | 747.52 | [M-H]- | C43H74NO7P |
| LIPID-N-0734 | PE(O-16:1_16:0) | GP | PE-O | 674.51 | 675.52 | [M-H]- | C37H74NO7P |
| LIPID-N-0821 | PE(O-20:1_20:4) | GP | PE-O | 778.58 | 779.58 | [M-H]- | C45H82NO7P |
| LIPID-N-0738 | PE(O-18:0_18:1) | GP | PE-O | 730.58 | 731.58 | [M-H]- | C41H82NO7P |
| LIPID-N-0820 | PE(O-18:1_22:4) | GP | PE-O | 778.58 | 779.58 | [M-H]- | C45H82NO7P |
| LIPID-N-0759 | PE(O-16:1_22:1) | GP | PE-O | 756.59 | 757.60 | [M-H]- | C43H84NO7P |
| LIPID-N-0758 | PE(O-18:1_20:1) | GP | PE-O | 756.59 | 757.60 | [M-H]- | C43H84NO7P |
| LIPID-N-0757 | PE(O-20:0_18:2) | GP | PE-O | 756.59 | 757.60 | [M-H]- | C43H84NO7P |
| LIPID-N-0755 | PE(O-16:1_20:1) | GP | PE-O | 728.56 | 729.57 | [M-H]- | C41H80NO7P |
| LIPID-N-0753 | PE(O-18:1_18:1) | GP | PE-O | 728.56 | 729.57 | [M-H]- | C41H80NO7P |
| LIPID-N-0748 | PE(O-16:1_16:1) | GP | PE-O | 672.50 | 673.50 | [M-H]- | C37H72NO7P |
| LIPID-N-0741 | PE(O-20:0_18:1) | GP | PE-O | 758.61 | 759.61 | [M-H]- | C43H86NO7P |
| LIPID-N-0739 | PE(O-18:1_18:0) | GP | PE-O | 730.58 | 731.58 | [M-H]- | C41H82NO7P |
| LIPID-N-0773 | PE(O-18:1_18:2) | GP | PE-O | 726.54 | 727.55 | [M-H]- | C41H78NO7P |
| LIPID-N-0750 | PE(O-16:1_18:1) | GP | PE-O | 700.53 | 701.54 | [M-H]- | C39H76NO7P |
| LIPID-N-0778 | PE(O-18:2_20:1) | GP | PE-O | 754.58 | 755.58 | [M-H]- | C43H82NO7P |
| LIPID-N-0783 | PE(O-18:1_22:2) | GP | PE-O | 782.61 | 783.61 | [M-H]- | C45H86NO7P |
| LIPID-N-0789 | PE(O-16:1_20:3) | GP | PE-O | 724.53 | 725.54 | [M-H]- | C41H76NO7P |
| LIPID-N-0790 | PE(O-18:3_18:1) | GP | PE-O | 724.53 | 725.54 | [M-H]- | C41H76NO7P |
| LIPID-N-0796 | PE(O-18:0_22:4) | GP | PE-O | 780.59 | 781.60 | [M-H]- | C45H84NO7P |
| LIPID-N-0730 | PE(O-18:0_16:0) | GP | PE-O | 704.56 | 705.57 | [M-H]- | C39H80NO7P |
| LIPID-N-0797 | PE(O-20:0_20:4) | GP | PE-O | 780.59 | 781.60 | [M-H]- | C45H84NO7P |
| LIPID-N-0808 | PE(O-18:3_18:2) | GP | PE-O | 722.51 | 723.52 | [M-H]- | C41H74NO7P |
| LIPID-N-0809 | PE(O-16:1_20:4) | GP | PE-O | 722.51 | 723.52 | [M-H]- | C41H74NO7P |
| LIPID-N-0777 | PE(O-20:1_18:2) | GP | PE-O | 754.58 | 755.58 | [M-H]- | C43H82NO7P |
| LIPID-N-0814 | PE(O-18:1_20:4) | GP | PE-O | 750.54 | 751.55 | [M-H]- | C43H78NO7P |
| LIPID-P-1386 | PE(P-14:0_22:6) | GP | PE-P | 720.50 | 719.49 | [M+H]+ | C41H70NO7P |
| LIPID-P-1387 | PE(P-18:1_20:5) | GP | PE-P | 748.53 | 747.52 | [M+H]+ | C43H74NO7P |
| LIPID-P-1407 | PE(P-20:2_22:6) | GP | PE-P | 800.56 | 799.55 | [M+H]+ | C47H78NO7P |
| LIPID-P-1390 | PE(P-18:1_22:5) | GP | PE-P | 776.56 | 775.55 | [M+H]+ | C45H78NO7P |
| LIPID-P-1401 | PE(P-20:1_22:6) | GP | PE-P | 802.58 | 801.57 | [M+H]+ | C47H80NO7P |
| LIPID-P-1405 | PE(P-18:2_22:6) | GP | PE-P | 772.53 | 771.52 | [M+H]+ | C45H74NO7P |
| LIPID-P-1389 | PE(P-17:0_22:6) | GP | PE-P | 762.54 | 761.54 | [M+H]+ | C44H76NO7P |
| LIPID-P-1377 | PE(P-18:1_22:4) | GP | PE-P | 778.58 | 777.57 | [M+H]+ | C45H80NO7P |
| LIPID-P-1357 | PE(P-18:1_20:3) | GP | PE-P | 752.56 | 751.55 | [M+H]+ | C43H78NO7P |
| LIPID-P-1375 | PE(P-18:0_20:5) | GP | PE-P | 750.54 | 749.54 | [M+H]+ | C43H76NO7P |
| LIPID-P-1309 | PE(P-16:0_16:0) | GP | PE-P | 676.53 | 675.52 | [M+H]+ | C37H74NO7P |
| LIPID-P-1315 | PE(P-14:0_18:1) | GP | PE-P | 674.51 | 673.50 | [M+H]+ | C37H72NO7P |
| LIPID-P-1317 | PE(P-17:0_18:1) | GP | PE-P | 716.56 | 715.55 | [M+H]+ | C40H78NO7P |
| LIPID-P-1318 | PE(P-16:0_20:1) | GP | PE-P | 730.58 | 729.57 | [M+H]+ | C41H80NO7P |
| LIPID-P-1322 | PE(P-20:0_18:1) | GP | PE-P | 758.61 | 757.60 | [M+H]+ | C43H84NO7P |
| LIPID-P-1323 | PE(P-18:0_20:1) | GP | PE-P | 758.61 | 757.60 | [M+H]+ | C43H84NO7P |
| LIPID-P-1329 | PE(P-18:1_16:1) | GP | PE-P | 700.53 | 699.52 | [M+H]+ | C39H74NO7P |
| LIPID-P-1330 | PE(P-16:0_20:2) | GP | PE-P | 728.56 | 727.55 | [M+H]+ | C41H78NO7P |
| LIPID-P-1331 | PE(P-18:0_18:2) | GP | PE-P | 728.56 | 727.55 | [M+H]+ | C41H78NO7P |
| LIPID-P-1376 | PE(P-16:0_24:5) | GP | PE-P | 778.58 | 777.57 | [M+H]+ | C45H80NO7P |
| LIPID-P-1340 | PE(P-16:0_20:3) | GP | PE-P | 726.54 | 725.54 | [M+H]+ | C41H76NO7P |
| LIPID-P-1343 | PE(P-16:1_22:2) | GP | PE-P | 754.58 | 753.57 | [M+H]+ | C43H80NO7P |
| LIPID-P-1345 | PE(P-16:0_22:3) | GP | PE-P | 754.58 | 753.57 | [M+H]+ | C43H80NO7P |
| LIPID-P-1352 | PE(P-14:0_20:4) | GP | PE-P | 696.50 | 695.49 | [M+H]+ | C39H70NO7P |
| LIPID-P-1353 | PE(P-18:2_18:2) | GP | PE-P | 724.53 | 723.52 | [M+H]+ | C41H74NO7P |
| LIPID-P-1355 | PE(P-16:0_22:4) | GP | PE-P | 752.56 | 751.55 | [M+H]+ | C43H78NO7P |
| LIPID-P-1359 | PE(P-18:2_20:2) | GP | PE-P | 752.56 | 751.55 | [M+H]+ | C43H78NO7P |
| LIPID-P-1373 | PE(P-16:1_20:4) | GP | PE-P | 722.51 | 721.50 | [M+H]+ | C41H72NO7P |
| LIPID-P-1374 | PE(P-16:0_22:5) | GP | PE-P | 750.54 | 749.54 | [M+H]+ | C43H76NO7P |
| LIPID-P-0652 | PE(P-16:0_20:4) | GP | PE-P | 724.53 | 723.52 | [M+H]+ | C41H74NO7P |
| LIPID-P-0654 | PE(P-18:0_20:4) | GP | PE-P | 752.56 | 751.55 | [M+H]+ | C43H78NO7P |
| LIPID-P-0646 | PE(P-15:0_20:3) | GP | PE-P | 712.53 | 711.52 | [M+H]+ | C40H74NO7P |
| LIPID-P-0630 | PE(P-18:0_16:0) | GP | PE-P | 704.56 | 703.55 | [M+H]+ | C39H78NO7P |
| LIPID-P-0631 | PE(P-18:0_18:0) | GP | PE-P | 732.59 | 731.58 | [M+H]+ | C41H82NO7P |
| LIPID-P-0632 | PE(P-18:0_20:0) | GP | PE-P | 760.62 | 759.61 | [M+H]+ | C43H86NO7P |
| LIPID-P-0651 | PE(P-15:0_20:4) | GP | PE-P | 710.51 | 709.50 | [M+H]+ | C40H72NO7P |
| LIPID-P-0661 | PE(P-16:0_22:6) | GP | PE-P | 748.53 | 747.52 | [M+H]+ | C43H74NO7P |
| LIPID-P-0650 | PE(P-18:0_22:3) | GP | PE-P | 782.61 | 781.60 | [M+H]+ | C45H84NO7P |
| LIPID-P-0649 | PE(P-18:0_20:3) | GP | PE-P | 754.58 | 753.57 | [M+H]+ | C43H80NO7P |
| LIPID-P-0637 | PE(P-18:0_18:1) | GP | PE-P | 730.58 | 729.57 | [M+H]+ | C41H80NO7P |
| LIPID-P-0640 | PE(P-16:0_18:2) | GP | PE-P | 700.53 | 699.52 | [M+H]+ | C39H74NO7P |
| LIPID-P-0664 | PE(P-18:1_22:6) | GP | PE-P | 774.54 | 773.54 | [M+H]+ | C45H76NO7P |
| LIPID-P-0648 | PE(P-17:0_20:3) | GP | PE-P | 740.56 | 739.55 | [M+H]+ | C42H78NO7P |
| LIPID-P-0660 | PE(P-18:0_22:5) | GP | PE-P | 778.58 | 777.57 | [M+H]+ | C45H80NO7P |
| LIPID-P-0658 | PE(P-18:1_20:4) | GP | PE-P | 750.54 | 749.54 | [M+H]+ | C43H76NO7P |
| LIPID-P-0655 | PE(P-18:0_22:4) | GP | PE-P | 780.59 | 779.58 | [M+H]+ | C45H82NO7P |
| LIPID-P-0647 | PE(P-18:1_18:2) | GP | PE-P | 726.54 | 725.54 | [M+H]+ | C41H76NO7P |
| LIPID-N-0432 | PG(16:0_16:0) | GP | PG | 721.51 | 722.51 | [M-H]- | C38H75O10P |
| LIPID-N-0438 | PG(16:0_18:1) | GP | PG | 747.53 | 748.53 | [M-H]- | C40H77O10P |
| LIPID-N-0442 | PG(18:1_16:1) | GP | PG | 745.51 | 746.51 | [M-H]- | C40H75O10P |
| LIPID-N-0444 | PG(18:2_16:0) | GP | PG | 745.51 | 746.51 | [M-H]- | C40H75O10P |
| LIPID-N-0446 | PG(18:1_18:1) | GP | PG | 773.54 | 774.54 | [M-H]- | C42H79O10P |
| LIPID-N-0447 | PG(18:0_18:2) | GP | PG | 773.54 | 774.54 | [M-H]- | C42H79O10P |
| LIPID-N-1140 | PG(16:0_22:1) | GP | PG | 803.58 | 804.59 | [M-H]- | C44H85O10P |
| LIPID-N-1114 | PG(17:0_18:0) | GP | PG | 763.55 | 764.56 | [M-H]- | C41H81O10P |
| LIPID-N-1115 | PG(16:0_20:0) | GP | PG | 777.56 | 778.57 | [M-H]- | C42H83O10P |
| LIPID-N-1147 | PG(18:0_22:1) | GP | PG | 831.61 | 832.62 | [M-H]- | C46H89O10P |
| LIPID-N-1152 | PG(16:0_16:2) | GP | PG | 717.47 | 718.48 | [M-H]- | C38H71O10P |
| LIPID-N-1155 | PG(16:0_17:2) | GP | PG | 731.49 | 732.49 | [M-H]- | C39H73O10P |
| LIPID-N-1164 | PG(18:1_21:1) | GP | PG | 815.58 | 816.59 | [M-H]- | C45H85O10P |
| LIPID-N-1171 | PG(16:1_16:2) | GP | PG | 715.46 | 716.46 | [M-H]- | C38H69O10P |
| LIPID-N-0449 | PG(20:1_18:1) | GP | PG | 801.57 | 802.57 | [M-H]- | C44H83O10P |
| LIPID-N-1173 | PG(16:0_18:3) | GP | PG | 743.49 | 744.49 | [M-H]- | C40H73O10P |
| LIPID-N-1178 | PG(20:1_18:2) | GP | PG | 799.55 | 800.56 | [M-H]- | C44H81O10P |
| LIPID-N-1179 | PG(18:1_21:2) | GP | PG | 813.56 | 814.57 | [M-H]- | C45H83O10P |
| LIPID-N-1183 | PG(22:1_18:2) | GP | PG | 827.58 | 828.59 | [M-H]- | C46H85O10P |
| LIPID-N-1134 | PG(19:0_15:1) | GP | PG | 747.52 | 748.53 | [M-H]- | C40H77O10P |
| LIPID-N-1119 | PG(18:0_19:0) | GP | PG | 791.58 | 792.59 | [M-H]- | C43H85O10P |
| LIPID-N-1174 | PG(16:1_18:2) | GP | PG | 743.49 | 744.49 | [M-H]- | C40H73O10P |
| LIPID-N-0451 | PG(18:2_18:1) | GP | PG | 771.53 | 772.53 | [M-H]- | C42H77O10P |
| LIPID-N-1135 | PG(18:0_16:1) | GP | PG | 747.52 | 748.53 | [M-H]- | C40H77O10P |
| LIPID-N-0453 | PG(18:2_18:2) | GP | PG | 769.51 | 770.51 | [M-H]- | C42H75O10P |
| LIPID-N-1188 | PG(16:1_20:3) | GP | PG | 769.50 | 770.51 | [M-H]- | C42H75O10P |
| LIPID-N-1189 | PG(18:1_18:3) | GP | PG | 769.50 | 770.51 | [M-H]- | C42H75O10P |
| LIPID-N-1192 | PG(18:2_21:2) | GP | PG | 811.55 | 812.56 | [M-H]- | C45H81O10P |
| LIPID-N-1195 | PG(18:2_22:2) | GP | PG | 825.56 | 826.57 | [M-H]- | C46H83O10P |
| LIPID-N-1205 | PG(16:0_20:5) | GP | PG | 767.49 | 768.49 | [M-H]- | C42H73O10P |
| LIPID-N-1194 | PG(18:1_22:3) | GP | PG | 825.56 | 826.57 | [M-H]- | C46H83O10P |
| LIPID-N-1220 | PG(20:1_22:6) | GP | PG | 847.55 | 848.56 | [M-H]- | C48H81O10P |
| LIPID-N-1221 | PG(20:4_20:4) | GP | PG | 817.50 | 818.51 | [M-H]- | C46H75O10P |
| LIPID-N-1223 | PG(20:4_22:6) | GP | PG | 841.50 | 842.51 | [M-H]- | C48H75O10P |
| LIPID-N-1225 | PG(22:6_22:6) | GP | PG | 865.50 | 866.51 | [M-H]- | C50H75O10P |
| LIPID-N-0455 | PG(18:0_20:4) | GP | PG | 797.54 | 798.54 | [M-H]- | C44H79O10P |
| LIPID-N-0454 | PG(16:0_20:4) | GP | PG | 769.51 | 770.51 | [M-H]- | C42H75O10P |
| LIPID-N-1212 | PG(19:2_20:4) | GP | PG | 807.52 | 808.53 | [M-H]- | C45H77O10P |
| LIPID-N-1390 | PI(18:0_14:1) | GP | PI | 807.50 | 808.51 | [M-H]- | C41H77O13P |
| LIPID-N-1407 | PI(15:0_19:2) | GP | PI | 833.52 | 834.53 | [M-H]- | C43H79O13P |
| LIPID-N-1406 | PI(15:1_18:1) | GP | PI | 819.50 | 820.51 | [M-H]- | C42H77O13P |
| LIPID-N-1403 | PI(15:1_16:1) | GP | PI | 791.47 | 792.48 | [M-H]- | C40H73O13P |
| LIPID-N-1402 | PI(13:1_18:1) | GP | PI | 791.47 | 792.48 | [M-H]- | C40H73O13P |
| LIPID-N-1397 | PI(15:0_21:1) | GP | PI | 863.56 | 864.57 | [M-H]- | C45H85O13P |
| LIPID-N-1388 | PI(18:0_13:1) | GP | PI | 793.49 | 794.49 | [M-H]- | C40H75O13P |
| LIPID-N-1373 | PI(10:0_16:0) | GP | PI | 725.42 | 726.43 | [M-H]- | C35H67O13P |
| LIPID-N-1386 | PI(12:0_18:1) | GP | PI | 779.47 | 780.48 | [M-H]- | C39H73O13P |
| LIPID-N-1378 | PI(16:0_17:0) | GP | PI | 823.53 | 824.54 | [M-H]- | C42H81O13P |
| LIPID-N-1376 | PI(16:0_16:0) | GP | PI | 809.52 | 810.53 | [M-H]- | C41H79O13P |
| LIPID-N-1374 | PI(12:0_18:0) | GP | PI | 781.49 | 782.49 | [M-H]- | C39H75O13P |
| LIPID-N-0522 | PI(16:0_20:4) | GP | PI | 857.53 | 858.53 | [M-H]- | C45H79O13P |
| LIPID-N-1387 | PI(13:0_18:1) | GP | PI | 793.49 | 794.49 | [M-H]- | C40H75O13P |
| LIPID-N-1416 | PI(16:0_18:3) | GP | PI | 831.50 | 832.51 | [M-H]- | C43H77O13P |
| LIPID-N-1423 | PI(18:0_20:3) | GP | PI | 887.56 | 888.57 | [M-H]- | C47H85O13P |
| LIPID-N-0508 | PI(18:1_18:2) | GP | PI | 859.54 | 860.54 | [M-H]- | C45H81O13P |
| LIPID-N-0512 | PI(18:1_19:2) | GP | PI | 873.55 | 874.56 | [M-H]- | C46H83O13P |
| LIPID-N-0493 | PI(17:1_18:1) | GP | PI | 847.53 | 848.54 | [M-H]- | C44H81O13P |
| LIPID-N-0551 | PI(18:0_22:6) | GP | PI | 909.56 | 910.56 | [M-H]- | C49H83O13P |
| LIPID-N-0548 | PI(16:0_22:6) | GP | PI | 881.53 | 882.53 | [M-H]- | C47H79O13P |
| LIPID-N-0491 | PI(16:1_18:1) | GP | PI | 833.53 | 834.53 | [M-H]- | C43H79O13P |
| LIPID-N-0490 | PI(18:2_16:0) | GP | PI | 833.53 | 834.53 | [M-H]- | C43H79O13P |
| LIPID-N-0482 | PI(18:0_18:1) | GP | PI | 863.57 | 864.57 | [M-H]- | C45H85O13P |
| LIPID-N-0495 | PI(18:0_18:2) | GP | PI | 861.56 | 862.56 | [M-H]- | C45H83O13P |
| LIPID-N-0538 | PI(20:4_18:1) | GP | PI | 883.54 | 884.54 | [M-H]- | C47H81O13P |
| LIPID-N-0525 | PI(20:3_18:1) | GP | PI | 885.56 | 886.56 | [M-H]- | C47H83O13P |
| LIPID-N-0524 | PI(18:0_20:4) | GP | PI | 885.56 | 886.56 | [M-H]- | C47H83O13P |
| LIPID-N-0523 | PI(17:0_20.4) | GP | PI | 871.53 | 872.54 | [M-H]- | C46H81O13P |
| LIPID-N-1441 | PI(18:1_20:4) | GP | PI | 883.53 | 884.54 | [M-H]- | C47H81O13P |
| LIPID-N-1439 | PI(15:1_20:4) | GP | PI | 841.49 | 842.49 | [M-H]- | C44H75O13P |
| LIPID-N-1436 | PI(18:1_20:3) | GP | PI | 885.55 | 886.56 | [M-H]- | C47H83O13P |
| LIPID-N-1433 | PI(16:0_22:4) | GP | PI | 885.55 | 886.56 | [M-H]- | C47H83O13P |
| LIPID-N-0496 | PI(18:1_18:1) | GP | PI | 861.56 | 862.56 | [M-H]- | C45H83O13P |
| LIPID-N-0877 | PMeOH(16:0_16:0) | GP | PMeOH | 661.48 | 662.49 | [M-H]- | C36H71O8P |
| LIPID-N-1357 | PS(18:3_20:4) | GP | PS | 804.48 | 817.58 | [M-H]- | C44H84NO10P |
| LIPID-N-1361 | PS(20:3_20:4) | GP | PS | 832.51 | 825.46 | [M-H]- | C46H68NO10P |
| LIPID-N-1365 | PS(18:2_22:6) | GP | PS | 830.50 | 825.46 | [M-H]- | C46H68NO10P |
| LIPID-N-1352 | PS(20:2_22:4) | GP | PS | 862.56 | 863.57 | [M-H]- | C48H82NO10P |
| LIPID-N-1259 | PS(18:0_15:1) | GP | PS | 746.50 | 749.52 | [M-H]- | C39H76NO10P |
| LIPID-N-0599 | PS(18:1_22:6) | GP | PS | 832.51 | 833.52 | [M-H]- | C46H76NO10P |
| LIPID-N-0598 | PS(18:0_22:6) | GP | PS | 834.53 | 835.54 | [M-H]- | C46H78NO10P |
| LIPID-N-0596 | PS(16:0_22:6) | GP | PS | 806.50 | 807.51 | [M-H]- | C44H74NO10P |
| LIPID-N-0595 | PS(18:0_22:5) | GP | PS | 836.54 | 837.55 | [M-H]- | C46H80NO10P |
| LIPID-N-0589 | PS(18:0_20:4) | GP | PS | 810.53 | 811.54 | [M-H]- | C44H78NO10P |
| LIPID-N-0577 | PS(18:0_18:2) | GP | PS | 786.53 | 787.54 | [M-H]- | C42H78NO10P |
| LIPID-N-0576 | PS(18:0_17:2) | GP | PS | 772.51 | 773.52 | [M-H]- | C41H76NO10P |
| LIPID-N-0563 | PS(18:0_18:0) | GP | PS | 790.56 | 791.57 | [M-H]- | C42H82NO10P |
| LIPID-N-1368 | PS(20:3_22:6) | GP | PS | 856.51 | 863.57 | [M-H]- | C48H82NO10P |
| LIPID-N-1346 | PS(18:1_22:5) | GP | PS | 834.53 | 825.46 | [M-H]- | C46H68NO10P |
| LIPID-N-1332 | PS(18:2_20:3) | GP | PS | 808.51 | 817.58 | [M-H]- | C44H84NO10P |
| LIPID-N-1292 | PS(18:1_18:1) | GP | PS | 786.53 | 787.54 | [M-H]- | C42H78NO10P |
| LIPID-N-1342 | PS(18:2_20:4) | GP | PS | 806.50 | 817.58 | [M-H]- | C44H84NO10P |
| LIPID-N-1238 | PS(17:0_18:0) | GP | PS | 776.54 | 777.55 | [M-H]- | C41H80NO10P |
| LIPID-N-1247 | PS(18:0_21:0) | GP | PS | 832.61 | 817.58 | [M-H]- | C44H84NO10P |
| LIPID-N-1296 | PS(19:0_18:2) | GP | PS | 800.54 | 801.55 | [M-H]- | C43H80NO10P |
| LIPID-N-1233 | PS(16:0_17:0) | GP | PS | 748.51 | 749.52 | [M-H]- | C39H76NO10P |
| LIPID-N-1307 | PS(16:0_20:3) | GP | PS | 784.51 | 785.52 | [M-H]- | C42H76NO10P |
| LIPID-N-1314 | PS(18:1_21:2) | GP | PS | 826.56 | 827.57 | [M-H]- | C45H82NO10P |
| LIPID-N-1318 | PS(16:1_18:3) | GP | PS | 754.47 | 755.47 | [M-H]- | C40H70NO10P |
| LIPID-N-1321 | PS(18:2_18:2) | GP | PS | 782.50 | 785.52 | [M-H]- | C42H76NO10P |
| LIPID-N-1322 | PS(18:1_20:3) | GP | PS | 810.53 | 817.58 | [M-H]- | C44H84NO10P |
| LIPID-N-1301 | PS(21:0_18:2) | GP | PS | 828.58 | 829.58 | [M-H]- | C45H84NO10P |
| LIPID-P-0336 | SHexCer(d18:1:/24:1) | SP | SHexCer | 890.80 | 889.63 | [M+H]+ | C48H91NO11S |
| LIPID-P-0334 | SHexCer(d18:1:/24:0) | SP | SHexCer | 892.80 | 891.65 | [M+H]+ | C48H93NO11S |
| LIPID-P-0331 | SHexCer(d18:1:/16:0(OH)) | SP | SHexCer | 796.80 | 795.52 | [M+H]+ | C40H77NO12S |
| LIPID-P-0332 | SHexCer(d18:1:/16:0) | SP | SHexCer | 780.80 | 779.52 | [M+H]+ | C40H77NO11S |
| LIPID-P-0754 | SM(d18:1/20:1) | SP | SM | 757.62 | 756.61 | [M+H]+ | C43H85N2O6P |
| LIPID-P-0752 | SM(d18:1/18:1) | SP | SM | 729.59 | 728.58 | [M+H]+ | C41H81N2O6P |
| LIPID-P-0750 | SM(d18:1/16:1) | SP | SM | 701.56 | 700.55 | [M+H]+ | C39H77N2O6P |
| LIPID-P-0751 | SM(d18:1/17:1) | SP | SM | 715.58 | 714.57 | [M+H]+ | C40H79N2O6P |
| LIPID-P-0756 | SM(d18:1/22:1) | SP | SM | 785.65 | 784.65 | [M+H]+ | C45H89N2O6P |
| LIPID-P-0763 | SM(d18:2/22:1) | SP | SM | 783.64 | 782.63 | [M+H]+ | C45H87N2O6P |
| LIPID-P-0758 | SM(d18:1/24:1) | SP | SM | 813.68 | 812.68 | [M+H]+ | C47H93N2O6P |
| LIPID-P-0759 | SM(d18:1/25:1) | SP | SM | 827.70 | 826.69 | [M+H]+ | C48H95N2O6P |
| LIPID-P-0760 | SM(d18:1/26:1) | SP | SM | 841.72 | 840.71 | [M+H]+ | C49H97N2O6P |
| LIPID-P-0761 | SM(d18:2/18:1) | SP | SM | 727.58 | 726.57 | [M+H]+ | C41H79N2O6P |
| LIPID-P-0762 | SM(d18:2/20:1) | SP | SM | 755.61 | 754.60 | [M+H]+ | C43H83N2O6P |
| LIPID-P-0749 | SM(d18:2/14:0) | SP | SM | 673.53 | 672.52 | [M+H]+ | C37H73N2O6P |
| LIPID-P-0764 | SM(d18:2/23:1) | SP | SM | 797.65 | 796.65 | [M+H]+ | C46H89N2O6P |
| LIPID-P-0765 | SM(d18:2/24:1) | SP | SM | 811.67 | 810.66 | [M+H]+ | C47H91N2O6P |
| LIPID-P-0766 | SM(d18:2/25:1) | SP | SM | 825.68 | 824.68 | [M+H]+ | C48H93N2O6P |
| LIPID-P-0767 | SM(d18:2/26:1) | SP | SM | 839.70 | 838.69 | [M+H]+ | C49H95N2O6P |
| LIPID-P-0768 | SM(d18:2/24:3) | SP | SM | 807.64 | 806.63 | [M+H]+ | C47H87N2O6P |
| LIPID-P-0757 | SM(d18:1/23:1) | SP | SM | 799.67 | 798.66 | [M+H]+ | C46H91N2O6P |
| LIPID-P-0747 | SM(d18:1/25:0) | SP | SM | 829.72 | 828.71 | [M+H]+ | C48H97N2O6P |
| LIPID-P-0748 | SM(d20:0/24:1) | SP | SM | 843.73 | 842.72 | [M+H]+ | C49H99N2O6P |
| LIPID-P-0730 | SM(d18:0/16:0) | SP | SM | 705.59 | 704.58 | [M+H]+ | C39H81N2O6P |
| LIPID-P-0729 | SM(d18:0/14:0) | SP | SM | 677.56 | 676.55 | [M+H]+ | C37H77N2O6P |
| LIPID-P-0746 | SM(d18:1/24:0) | SP | SM | 815.70 | 814.69 | [M+H]+ | C47H95N2O6P |
| LIPID-P-0731 | SM(d18:0/17:0) | SP | SM | 719.61 | 718.60 | [M+H]+ | C40H83N2O6P |
| LIPID-P-0732 | SM(d18:0/18:0) | SP | SM | 733.62 | 732.61 | [M+H]+ | C41H85N2O6P |
| LIPID-P-0733 | SM(d18:0/20:0) | SP | SM | 761.65 | 760.65 | [M+H]+ | C43H89N2O6P |
| LIPID-P-0734 | SM(d18:0/22:0) | SP | SM | 789.68 | 788.68 | [M+H]+ | C45H93N2O6P |
| LIPID-P-0736 | SM(d18:1/14:0) | SP | SM | 675.54 | 674.54 | [M+H]+ | C37H75N2O6P |
| LIPID-P-0737 | SM(d18:1/15:0) | SP | SM | 689.56 | 688.55 | [M+H]+ | C38H77N2O6P |
| LIPID-P-0738 | SM(d18:1/16:0) | SP | SM | 703.58 | 702.57 | [M+H]+ | C39H79N2O6P |
| LIPID-P-0735 | SM(d18:1/12:0) | SP | SM | 647.51 | 646.50 | [M+H]+ | C35H71N2O6P |
| LIPID-P-0740 | SM(d18:1/18:0) | SP | SM | 731.61 | 730.60 | [M+H]+ | C41H83N2O6P |
| LIPID-P-0739 | SM(d18:1/17:0) | SP | SM | 717.59 | 716.58 | [M+H]+ | C40H81N2O6P |
| LIPID-P-0744 | SM(d18:1/22:0) | SP | SM | 787.67 | 786.66 | [M+H]+ | C45H91N2O6P |
| LIPID-P-0743 | SM(d18:1/21:0) | SP | SM | 773.65 | 772.65 | [M+H]+ | C44H89N2O6P |
| LIPID-P-0742 | SM(d18:1/20:0) | SP | SM | 759.64 | 758.63 | [M+H]+ | C43H87N2O6P |
| LIPID-P-0745 | SM(d18:1/23:0) | SP | SM | 801.68 | 800.68 | [M+H]+ | C46H93N2O6P |
| LIPID-P-0741 | SM(d18:1/19:0) | SP | SM | 745.62 | 744.61 | [M+H]+ | C42H85N2O6P |
| LIPID-P-0096 | SPH(d18:1) | SP | SPH | 300.29 | 299.28 | [M+H]+ | C18H37NO2 |
| LIPID-P-0097 | SPH(d18:2) | SP | SPH | 298.27 | 297.27 | [M+H]+ | C18H35NO2 |
| LIPID-P-0100 | S1P(d18:1) | SP | SPH | 380.26 | 379.25 | [M+H]+ | C18H38NO5P |
| LIPID-P-1281 | SPH(d20:1) | SP | SPH | 328.32 | 327.31 | [M+H]+ | C20H41NO2 |
| LIPID-P-0094 | SPH(d18:0) | SP | SPH | 302.31 | 301.30 | [M+H]+ | C18H39NO2 |
| LIPID-P-2369 | TG(18:1_18:2_24:5) | GL | TG | 976.85 | 958.80 | [M+NH4]+ | C63H106O6 |
| LIPID-P-2370 | TG(18:1_18:3_20:5) | GL | TG | 918.74 | 900.72 | [M+NH4]+ | C59H96O6 |
| LIPID-P-2371 | TG(18:1_18:3_22:3) | GL | TG | 950.82 | 932.78 | [M+NH4]+ | C61H104O6 |
| LIPID-P-2372 | TG(18:1_19:1_22:6) | GL | TG | 962.82 | 944.78 | [M+NH4]+ | C62H104O6 |
| LIPID-P-2373 | TG(18:1_20:1_20:2) | GL | TG | 956.87 | 938.83 | [M+NH4]+ | C61H110O6 |
| LIPID-P-2375 | TG(18:1_20:1_22:4) | GL | TG | 980.87 | 962.83 | [M+NH4]+ | C63H110O6 |
| LIPID-P-2390 | TG(8:0_10:0_10:0) | GL | TG | 544.46 | 526.42 | [M+NH4]+ | C31H58O6 |
| LIPID-P-2368 | TG(18:1_18:2_22:3) | GL | TG | 952.82 | 934.80 | [M+NH4]+ | C61H106O6 |
| LIPID-P-2377 | TG(18:1_20:2_20:4) | GL | TG | 950.81 | 932.78 | [M+NH4]+ | C61H104O6 |
| LIPID-P-2387 | TG(20:0_18:1_22:6) | GL | TG | 978.84 | 960.81 | [M+NH4]+ | C63H108O6 |
| LIPID-P-2385 | TG(18:3_20:4_20:4) | GL | TG | 942.76 | 924.72 | [M+NH4]+ | C61H96O6 |
| LIPID-P-2384 | TG(18:2_20:3_22:5) | GL | TG | 972.80 | 954.77 | [M+NH4]+ | C63H102O6 |
| LIPID-P-2382 | TG(18:2_18:2_24:5) | GL | TG | 974.82 | 956.78 | [M+NH4]+ | C63H104O6 |
| LIPID-P-2381 | TG(18:1_20:5_22:5) | GL | TG | 970.78 | 952.75 | [M+NH4]+ | C63H100O6 |
| LIPID-P-2378 | TG(18:1_20:2_22:4) | GL | TG | 978.84 | 960.81 | [M+NH4]+ | C63H108O6 |
| LIPID-P-2376 | TG(18:1_20:1_24:5) | GL | TG | 1006.87 | 988.85 | [M+NH4]+ | C65H112O6 |
| LIPID-P-2367 | TG(18:1_18:2_19:2) | GL | TG | 912.81 | 894.77 | [M+NH4]+ | C58H102O6 |
| LIPID-P-2346 | TG(18:0_20:2_18:3) | GL | TG | 926.82 | 908.78 | [M+NH4]+ | C59H104O6 |
| LIPID-P-2304 | TG(15:1_18:1_18:2) | GL | TG | 858.76 | 840.72 | [M+NH4]+ | C54H96O6 |
| LIPID-P-2303 | TG(16:0_15:1_18:2) | GL | TG | 832.73 | 814.71 | [M+NH4]+ | C52H94O6 |
| LIPID-P-2302 | TG(15:1_17:1_17:1) | GL | TG | 832.74 | 814.71 | [M+NH4]+ | C52H94O6 |
| LIPID-P-2300 | TG(14:0_17:2_18:2) | GL | TG | 830.73 | 812.69 | [M+NH4]+ | C52H92O6 |
| LIPID-P-2299 | TG(15:1_15:1_19:2) | GL | TG | 830.72 | 812.69 | [M+NH4]+ | C52H92O6 |
| LIPID-P-2297 | TG(15:0_18:1_20:5) | GL | TG | 882.75 | 864.72 | [M+NH4]+ | C56H96O6 |
| LIPID-P-2296 | TG(15:0_18:0_18:1) | GL | TG | 864.77 | 846.77 | [M+NH4]+ | C54H102O6 |
| LIPID-P-2294 | TG(11:0_18:1_18:2) | GL | TG | 804.71 | 786.67 | [M+NH4]+ | C50H90O6 |
| LIPID-P-2293 | TG(14:1_18:2_20:4) | GL | TG | 866.71 | 848.69 | [M+NH4]+ | C55H92O6 |
| LIPID-P-2292 | TG(14:1_16:1_20:5) | GL | TG | 838.69 | 820.66 | [M+NH4]+ | C53H88O6 |
| LIPID-P-2290 | TG(14:0_20:4_22:6) | GL | TG | 916.73 | 898.71 | [M+NH4]+ | C59H94O6 |
| LIPID-P-2289 | TG(14:0_20:2_22:6) | GL | TG | 920.76 | 902.74 | [M+NH4]+ | C59H98O6 |
| LIPID-P-2306 | TG(15:1_18:2_22:6) | GL | TG | 904.74 | 886.71 | [M+NH4]+ | C58H94O6 |
| LIPID-P-2288 | TG(14:0_18:3_20:5) | GL | TG | 864.70 | 846.67 | [M+NH4]+ | C55H90O6 |
| LIPID-P-2284 | TG(14:0_14:0_16:1) | GL | TG | 766.69 | 748.66 | [M+NH4]+ | C47H88O6 |
| LIPID-P-2282 | TG(15:0_18:2_22:5) | GL | TG | 908.75 | 890.74 | [M+NH4]+ | C58H98O6 |
| LIPID-P-2281 | TG(13:0_20:2_20:5) | GL | TG | 880.73 | 862.71 | [M+NH4]+ | C56H94O6 |
| LIPID-P-2280 | TG(13:0_18:1_22:6) | GL | TG | 880.74 | 862.71 | [M+NH4]+ | C56H94O6 |
| LIPID-P-2279 | TG(13:0_18:1_18:2) | GL | TG | 832.73 | 814.71 | [M+NH4]+ | C52H94O6 |
| LIPID-P-2278 | TG(13:0_16:2_18:2) | GL | TG | 802.68 | 784.66 | [M+NH4]+ | C50H88O6 |
| LIPID-P-2276 | TG(12:0_18:1_22:6) | GL | TG | 866.72 | 848.69 | [M+NH4]+ | C55H92O6 |
| LIPID-P-2275 | TG(12:0_16:2_18:2) | GL | TG | 788.68 | 770.64 | [M+NH4]+ | C49H86O6 |
| LIPID-P-2274 | TG(12:0_16:1_22:6) | GL | TG | 838.70 | 820.66 | [M+NH4]+ | C53H88O6 |
| LIPID-P-2273 | TG(12:0_16:0_18:3) | GL | TG | 790.69 | 772.66 | [M+NH4]+ | C49H88O6 |
| LIPID-P-2272 | TG(10:0_12:0_12:0) | GL | TG | 628.55 | 610.52 | [M+NH4]+ | C37H70O6 |
| LIPID-P-2287 | TG(14:0_18:2_18:4) | GL | TG | 840.71 | 822.67 | [M+NH4]+ | C53H90O6 |
| LIPID-P-2366 | TG(18:0_16:1_24:6) | GL | TG | 950.82 | 932.78 | [M+NH4]+ | C61H104O6 |
| LIPID-P-2307 | TG(16:0_16:0_20:2) | GL | TG | 876.80 | 858.77 | [M+NH4]+ | C55H102O6 |
| LIPID-P-2310 | TG(16:0_16:2_20:5) | GL | TG | 866.73 | 848.69 | [M+NH4]+ | C55H92O6 |
| LIPID-P-2360 | TG(20:1_18:3_18:3) | GL | TG | 922.79 | 904.75 | [M+NH4]+ | C59H100O6 |
| LIPID-P-2356 | TG(18:0_18:2_20:3) | GL | TG | 926.82 | 908.78 | [M+NH4]+ | C59H104O6 |
| LIPID-P-2355 | TG(18:0_18:2_20:2) | GL | TG | 928.83 | 910.80 | [M+NH4]+ | C59H106O6 |
| LIPID-P-2350 | TG(17:2_18:2_22:6) | GL | TG | 930.75 | 912.72 | [M+NH4]+ | C60H96O6 |
| LIPID-P-2349 | TG(17:2_18:2_18:3) | GL | TG | 880.72 | 862.71 | [M+NH4]+ | C56H94O6 |
| LIPID-P-2348 | TG(17:1_19:2_19:2) | GL | TG | 912.80 | 894.77 | [M+NH4]+ | C58H102O6 |
| LIPID-P-2347 | TG(17:1_18:2_22:6) | GL | TG | 932.77 | 914.74 | [M+NH4]+ | C60H98O6 |
| LIPID-P-2345 | TG(17:0_18:3_18:3) | GL | TG | 882.76 | 864.72 | [M+NH4]+ | C56H96O6 |
| LIPID-P-2342 | TG(17:0_18:2_18:3) | GL | TG | 884.77 | 866.74 | [M+NH4]+ | C56H98O6 |
| LIPID-P-2340 | TG(17:0_18:1_20:1) | GL | TG | 918.85 | 900.81 | [M+NH4]+ | C58H108O6 |
| LIPID-P-2336 | TG(19:0_18:2_18:2) | GL | TG | 914.80 | 896.78 | [M+NH4]+ | C58H104O6 |
| LIPID-P-2335 | TG(16:1_18:3_20:4) | GL | TG | 892.74 | 874.71 | [M+NH4]+ | C57H94O6 |
| LIPID-P-2334 | TG(16:1_18:1_20:5) | GL | TG | 894.76 | 876.72 | [M+NH4]+ | C57H96O6 |
| LIPID-P-2309 | TG(16:0_16:2_18:3) | GL | TG | 842.73 | 824.69 | [M+NH4]+ | C53H92O6 |
| LIPID-P-2333 | TG(16:1_17:1_22:6) | GL | TG | 906.75 | 888.72 | [M+NH4]+ | C58H96O6 |
| LIPID-P-2327 | TG(18:0_16:1_20:1) | GL | TG | 904.81 | 886.80 | [M+NH4]+ | C57H106O6 |
| LIPID-P-2326 | TG(18:1_17:2_18:2) | GL | TG | 884.78 | 866.74 | [M+NH4]+ | C56H98O6 |
| LIPID-P-2325 | TG(18:0_17:1_18:1) | GL | TG | 890.83 | 872.78 | [M+NH4]+ | C56H104O6 |
| LIPID-P-2324 | TG(18:1_18:3_16:4) | GL | TG | 864.71 | 846.67 | [M+NH4]+ | C55H90O6 |
| LIPID-P-2321 | TG(16:0_20:2_24:5) | GL | TG | 978.86 | 960.81 | [M+NH4]+ | C63H108O6 |
| LIPID-P-2320 | TG(16:0_20:2_22:6) | GL | TG | 948.79 | 930.77 | [M+NH4]+ | C61H102O6 |
| LIPID-P-2319 | TG(16:0_20:1_21:1) | GL | TG | 946.88 | 928.85 | [M+NH4]+ | C60H112O6 |
| LIPID-P-2318 | TG(16:1_18:1_16:4) | GL | TG | 840.71 | 822.67 | [M+NH4]+ | C53H90O6 |
| LIPID-P-2317 | TG(16:0_20:0_22:6) | GL | TG | 952.83 | 934.80 | [M+NH4]+ | C61H106O6 |
| LIPID-P-2314 | TG(16:0_18:5_20:5) | GL | TG | 888.72 | 870.67 | [M+NH4]+ | C57H90O6 |
| LIPID-P-2313 | TG(16:1_18:1_16:3) | GL | TG | 842.72 | 824.69 | [M+NH4]+ | C53H92O6 |
| LIPID-P-2312 | TG(16:0_17:2_20:4) | GL | TG | 882.75 | 864.72 | [M+NH4]+ | C56H96O6 |
| LIPID-P-2311 | TG(16:0_17:1_22:6) | GL | TG | 908.78 | 890.74 | [M+NH4]+ | C58H98O6 |
| LIPID-P-2331 | TG(18:1_16:2_20:5) | GL | TG | 892.74 | 874.71 | [M+NH4]+ | C57H94O6 |
| LIPID-P-2330 | TG(18:1_17:2_19:2) | GL | TG | 898.78 | 880.75 | [M+NH4]+ | C57H100O6 |
| LIPID-P-0938 | TG(19:0_18:1_20:1) | GL | TG | 946.87 | 928.85 | [M+NH4]+ | C60H112O6 |
| LIPID-P-1243 | TG(18:2_20:5_22:6) | GL | TG | 966.75 | 948.72 | [M+NH4]+ | C63H96O6 |
| LIPID-P-0933 | TG(18:0_18:1_20:1) | GL | TG | 932.87 | 914.83 | [M+NH4]+ | C59H110O6 |
| LIPID-P-0934 | TG(18:0_18:2_20:0) | GL | TG | 932.86 | 914.83 | [M+NH4]+ | C59H110O6 |
| LIPID-P-0935 | TG(16:0_18:1_22:1) | GL | TG | 932.86 | 914.83 | [M+NH4]+ | C59H110O6 |
| LIPID-P-0937 | TG(15:0_18:1_24:1) | GL | TG | 946.87 | 928.85 | [M+NH4]+ | C60H112O6 |
| LIPID-P-0939 | TG(22:0_18:1_18:1) | GL | TG | 960.89 | 942.86 | [M+NH4]+ | C61H114O6 |
| LIPID-P-0940 | TG(18:0_22:0_18:2) | GL | TG | 960.89 | 942.86 | [M+NH4]+ | C61H114O6 |
| LIPID-P-0942 | TG(16:0_18:1_24:1) | GL | TG | 960.89 | 942.86 | [M+NH4]+ | C61H114O6 |
| LIPID-P-0943 | TG(16:0_17:1_26:1) | GL | TG | 974.90 | 956.88 | [M+NH4]+ | C62H116O6 |
| LIPID-P-0944 | TG(23:0_18:1_18:1) | GL | TG | 974.90 | 956.88 | [M+NH4]+ | C62H116O6 |
| LIPID-P-0945 | TG(24:0_18:1_18:1) | GL | TG | 988.92 | 970.89 | [M+NH4]+ | C63H118O6 |
| LIPID-P-0946 | TG(18:0_20:1_22:1) | GL | TG | 988.93 | 970.89 | [M+NH4]+ | C63H118O6 |
| LIPID-P-0947 | TG(16:0_18:1_26:1) | GL | TG | 988.92 | 970.89 | [M+NH4]+ | C63H118O6 |
| LIPID-P-0949 | TG(24:0_18:1_20:1) | GL | TG | 1016.95 | 998.92 | [M+NH4]+ | C65H122O6 |
| LIPID-P-0950 | TG(8:0_16:1_16:2) | GL | TG | 706.59 | 688.56 | [M+NH4]+ | C43H76O6 |
| LIPID-P-0951 | TG(8:0_15:1_18:2) | GL | TG | 720.61 | 702.58 | [M+NH4]+ | C44H78O6 |
| LIPID-P-0952 | TG(8:0_16:1_18:2) | GL | TG | 734.63 | 716.60 | [M+NH4]+ | C45H80O6 |
| LIPID-P-0953 | TG(10:0_14:0_18:3) | GL | TG | 734.63 | 716.60 | [M+NH4]+ | C45H80O6 |
| LIPID-P-0931 | TG(16:0_18:1_21:1) | GL | TG | 918.85 | 900.81 | [M+NH4]+ | C58H108O6 |
| LIPID-P-0929 | TG(18:0_18:1_19:1) | GL | TG | 918.85 | 900.81 | [M+NH4]+ | C58H108O6 |
| LIPID-P-0927 | TG(16:0_16:1_22:1) | GL | TG | 904.83 | 886.80 | [M+NH4]+ | C57H106O6 |
| LIPID-P-0925 | TG(16:1_18:0_20:1) | GL | TG | 904.81 | 886.80 | [M+NH4]+ | C57H106O6 |
| LIPID-P-0901 | TG(16:0_16:1_16:1) | GL | TG | 820.74 | 802.71 | [M+NH4]+ | C51H94O6 |
| LIPID-P-0902 | TG(14:0_16:0_18:2) | GL | TG | 820.74 | 802.71 | [M+NH4]+ | C51H94O6 |
| LIPID-P-0903 | TG(14:0_16:1_18:1) | GL | TG | 820.74 | 802.71 | [M+NH4]+ | C51H94O6 |
| LIPID-P-0906 | TG(16:0_16:1_17:1) | GL | TG | 834.76 | 816.72 | [M+NH4]+ | C52H96O6 |
| LIPID-P-0907 | TG(15:0_16:0_18:2) | GL | TG | 834.75 | 816.72 | [M+NH4]+ | C52H96O6 |
| LIPID-P-0908 | TG(15:0_16:1_18:1) | GL | TG | 834.75 | 816.72 | [M+NH4]+ | C52H96O6 |
| LIPID-P-0910 | TG(16:0_16:1_18:1) | GL | TG | 848.77 | 830.74 | [M+NH4]+ | C53H98O6 |
| LIPID-P-0911 | TG(14:0_18:0_18:2) | GL | TG | 848.77 | 830.74 | [M+NH4]+ | C53H98O6 |
| LIPID-P-0954 | TG(8:0_16:0_18:3) | GL | TG | 734.63 | 716.60 | [M+NH4]+ | C45H80O6 |
| LIPID-P-0912 | TG(16:0_17:1_18:1) | GL | TG | 862.78 | 844.75 | [M+NH4]+ | C54H100O6 |
| LIPID-P-0915 | TG(16:0_18:1_18:1) | GL | TG | 876.80 | 858.77 | [M+NH4]+ | C55H102O6 |
| LIPID-P-0916 | TG(16:0_18:0_18:2) | GL | TG | 876.79 | 858.77 | [M+NH4]+ | C55H102O6 |
| LIPID-P-0918 | TG(14:0_18:0_20:2) | GL | TG | 876.80 | 858.77 | [M+NH4]+ | C55H102O6 |
| LIPID-P-0920 | TG(16:0_16:1_20:1) | GL | TG | 876.80 | 858.77 | [M+NH4]+ | C55H102O6 |
| LIPID-P-0921 | TG(17:0_18:1_18:1) | GL | TG | 890.79 | 872.78 | [M+NH4]+ | C56H104O6 |
| LIPID-P-0922 | TG(16:0_18:1_19:1) | GL | TG | 890.82 | 872.78 | [M+NH4]+ | C56H104O6 |
| LIPID-P-0923 | TG(18:0_18:1_18:1) | GL | TG | 904.83 | 886.80 | [M+NH4]+ | C57H106O6 |
| LIPID-P-0924 | TG(16:0_18:1_20:1) | GL | TG | 904.84 | 886.80 | [M+NH4]+ | C57H106O6 |
| LIPID-P-0913 | TG(15:0_17:0_19:2) | GL | TG | 862.78 | 844.75 | [M+NH4]+ | C54H100O6 |
| LIPID-P-0955 | TG(14:1_14:1_16:1) | GL | TG | 762.66 | 744.63 | [M+NH4]+ | C47H84O6 |
| LIPID-P-0956 | TG(8:0_18:1_18:2) | GL | TG | 762.66 | 744.63 | [M+NH4]+ | C47H84O6 |
| LIPID-P-0957 | TG(10:0_16:0_18:3) | GL | TG | 762.66 | 744.63 | [M+NH4]+ | C47H84O6 |
| LIPID-P-0990 | TG(18:0_18:1_18:2) | GL | TG | 902.82 | 884.78 | [M+NH4]+ | C57H104O6 |
| LIPID-P-0991 | TG(16:0_18:1_20:2) | GL | TG | 902.82 | 884.78 | [M+NH4]+ | C57H104O6 |
| LIPID-P-0993 | TG(17:1_18:1_20:1) | GL | TG | 916.83 | 898.80 | [M+NH4]+ | C58H106O6 |
| LIPID-P-0994 | TG(18:1_18:1_19:1) | GL | TG | 916.82 | 898.80 | [M+NH4]+ | C58H106O6 |
| LIPID-P-0997 | TG(18:1_18:2_20:0) | GL | TG | 930.85 | 912.81 | [M+NH4]+ | C59H108O6 |
| LIPID-P-0999 | TG(18:1_18:1_20:1) | GL | TG | 930.85 | 912.81 | [M+NH4]+ | C59H108O6 |
| LIPID-P-1000 | TG(16:1_18:1_22:1) | GL | TG | 930.84 | 912.81 | [M+NH4]+ | C59H108O6 |
| LIPID-P-1001 | TG(20:1_18:2_18:0) | GL | TG | 930.84 | 912.81 | [M+NH4]+ | C59H108O6 |
| LIPID-P-0988 | TG(18:1_18:1_18:1) | GL | TG | 902.82 | 884.78 | [M+NH4]+ | C57H104O6 |
| LIPID-P-1003 | TG(21:0_18:1_18:2) | GL | TG | 944.86 | 926.83 | [M+NH4]+ | C60H110O6 |
| LIPID-P-1007 | TG(16:1_18:1_24:1) | GL | TG | 958.87 | 940.85 | [M+NH4]+ | C61H112O6 |
| LIPID-P-1010 | TG(18:1_18:2_22:0) | GL | TG | 958.88 | 940.85 | [M+NH4]+ | C61H112O6 |
| LIPID-P-1011 | TG(18:1_20:1_20:1) | GL | TG | 958.88 | 940.85 | [M+NH4]+ | C61H112O6 |
| LIPID-P-1013 | TG(17:1_18:1_24:1) | GL | TG | 972.89 | 954.86 | [M+NH4]+ | C62H114O6 |
| LIPID-P-1014 | TG(23:0_18:1_18:2) | GL | TG | 972.89 | 954.86 | [M+NH4]+ | C62H114O6 |
| LIPID-P-1016 | TG(18:1_20:1_22:1) | GL | TG | 986.91 | 968.88 | [M+NH4]+ | C63H116O6 |
| LIPID-P-1017 | TG(18:1_18:1_24:1) | GL | TG | 986.91 | 968.88 | [M+NH4]+ | C63H116O6 |
| LIPID-P-1004 | TG(16:1_17:1_24:1) | GL | TG | 944.86 | 926.83 | [M+NH4]+ | C60H110O6 |
| LIPID-P-0900 | TG(14:0_16:1_17:1) | GL | TG | 806.72 | 788.69 | [M+NH4]+ | C50H92O6 |
| LIPID-P-0987 | TG(17:0_18:1_18:2) | GL | TG | 888.80 | 870.77 | [M+NH4]+ | C56H102O6 |
| LIPID-P-0983 | TG(14:0_18:1_20:2) | GL | TG | 874.79 | 856.75 | [M+NH4]+ | C55H100O6 |
| LIPID-P-0958 | TG(9:0_18:1_18:2) | GL | TG | 776.68 | 758.64 | [M+NH4]+ | C48H86O6 |
| LIPID-P-0959 | TG(14:1_15:1_16:1) | GL | TG | 776.68 | 758.64 | [M+NH4]+ | C48H86O6 |
| LIPID-P-0960 | TG(14:1_16:1_16:1) | GL | TG | 790.69 | 772.66 | [M+NH4]+ | C49H88O6 |
| LIPID-P-0961 | TG(12:0_16:1_18:2) | GL | TG | 790.69 | 772.66 | [M+NH4]+ | C49H88O6 |
| LIPID-P-0962 | TG(10:0_18:1_18:2) | GL | TG | 790.69 | 772.66 | [M+NH4]+ | C49H88O6 |
| LIPID-P-0965 | TG(14:1_16:1_18:1) | GL | TG | 818.72 | 800.69 | [M+NH4]+ | C51H92O6 |
| LIPID-P-0966 | TG(14:0_16:1_18:2) | GL | TG | 818.72 | 800.69 | [M+NH4]+ | C51H92O6 |
| LIPID-P-0969 | TG(16:0_14:1_18:2) | GL | TG | 818.72 | 800.69 | [M+NH4]+ | C51H92O6 |
| LIPID-P-0984 | TG(17:1_18:1_18:1) | GL | TG | 888.80 | 870.77 | [M+NH4]+ | C56H102O6 |
| LIPID-P-0970 | TG(16:1_16:1_17:1) | GL | TG | 832.74 | 814.71 | [M+NH4]+ | C52H94O6 |
| LIPID-P-0972 | TG(14:0_17:1_18:2) | GL | TG | 832.74 | 814.71 | [M+NH4]+ | C52H94O6 |
| LIPID-P-0973 | TG(16:1_16:1_18:1) | GL | TG | 846.75 | 828.72 | [M+NH4]+ | C53H96O6 |
| LIPID-P-0976 | TG(16:0_16:1_18:2) | GL | TG | 846.76 | 828.72 | [M+NH4]+ | C53H96O6 |
| LIPID-P-0977 | TG(16:1_17:1_18:1) | GL | TG | 860.77 | 842.74 | [M+NH4]+ | C54H98O6 |
| LIPID-P-0978 | TG(15:0_18:1_18:2) | GL | TG | 860.77 | 842.74 | [M+NH4]+ | C54H98O6 |
| LIPID-P-0979 | TG(16:0_17:1_18:2) | GL | TG | 860.77 | 842.74 | [M+NH4]+ | C54H98O6 |
| LIPID-P-0981 | TG(16:1_18:1_18:1) | GL | TG | 874.78 | 856.75 | [M+NH4]+ | C55H100O6 |
| LIPID-P-0982 | TG(16:0_18:1_18:2) | GL | TG | 874.79 | 856.75 | [M+NH4]+ | C55H100O6 |
| LIPID-P-0971 | TG(15:0_16:1_18:2) | GL | TG | 832.74 | 814.71 | [M+NH4]+ | C52H94O6 |
| LIPID-P-1020 | TG(24:0_18:1_18:2) | GL | TG | 986.91 | 968.88 | [M+NH4]+ | C63H116O6 |
| LIPID-P-0899 | TG(15:0_16:1_16:1) | GL | TG | 806.72 | 788.69 | [M+NH4]+ | C50H92O6 |
| LIPID-P-0894 | TG(14:0_14:1_18:1) | GL | TG | 792.71 | 774.67 | [M+NH4]+ | C49H90O6 |
| LIPID-P-0804 | TG(18:0_18:0_18:0) | GL | TG | 908.86 | 890.83 | [M+NH4]+ | C57H110O6 |
| LIPID-P-0809 | TG(16:0_18:0_22:0) | GL | TG | 936.90 | 918.86 | [M+NH4]+ | C59H114O6 |
| LIPID-P-0813 | TG(16:0_18:0_24:0) | GL | TG | 964.93 | 946.89 | [M+NH4]+ | C61H118O6 |
| LIPID-P-0819 | TG(8:0_16:0_16:1) | GL | TG | 710.63 | 692.60 | [M+NH4]+ | C43H80O6 |
| LIPID-P-0820 | TG(8:0_14:0_18:1) | GL | TG | 710.63 | 692.60 | [M+NH4]+ | C43H80O6 |
| LIPID-P-0821 | TG(10:0_12:0_18:1) | GL | TG | 710.63 | 692.60 | [M+NH4]+ | C43H80O6 |
| LIPID-P-0822 | TG(8:0_16:0_18:1) | GL | TG | 738.66 | 720.63 | [M+NH4]+ | C45H84O6 |
| LIPID-P-0823 | TG(12:0_14:0_16:1) | GL | TG | 738.66 | 720.63 | [M+NH4]+ | C45H84O6 |
| LIPID-P-0824 | TG(10:0_16:0_16:1) | GL | TG | 738.66 | 720.63 | [M+NH4]+ | C45H84O6 |
| LIPID-P-0825 | TG(12:0_15:0_16:1) | GL | TG | 752.67 | 734.64 | [M+NH4]+ | C46H86O6 |
| LIPID-P-0827 | TG(12:0_16:0_16:1) | GL | TG | 766.69 | 748.66 | [M+NH4]+ | C47H88O6 |
| LIPID-P-0828 | TG(10:0_16:0_18:1) | GL | TG | 766.69 | 748.66 | [M+NH4]+ | C47H88O6 |
| LIPID-P-0829 | TG(12:0_14:0_18:1) | GL | TG | 766.69 | 748.66 | [M+NH4]+ | C47H88O6 |
| LIPID-P-0801 | TG(15:0_18:0_20:0) | GL | TG | 894.84 | 876.81 | [M+NH4]+ | C56H108O6 |
| LIPID-P-0799 | TG(14:0_16:0_22:0) | GL | TG | 880.83 | 862.80 | [M+NH4]+ | C55H106O6 |
| LIPID-P-0770 | TG(8:0_14:0_16:0) | GL | TG | 684.61 | 666.58 | [M+NH4]+ | C41H78O6 |
| LIPID-P-0771 | TG(10:0_16:0_12:0) | GL | TG | 684.61 | 666.58 | [M+NH4]+ | C41H78O6 |
| LIPID-P-0773 | TG(10:0_14:0_16:0) | GL | TG | 712.64 | 694.61 | [M+NH4]+ | C43H82O6 |
| LIPID-P-0775 | TG(8:0_14:0_18:0) | GL | TG | 712.64 | 694.61 | [M+NH4]+ | C43H82O6 |
| LIPID-P-0776 | TG(10:0_15:0_16:0) | GL | TG | 726.66 | 708.63 | [M+NH4]+ | C44H84O6 |
| LIPID-P-0777 | TG(12:0_14:0_16:0) | GL | TG | 740.68 | 722.64 | [M+NH4]+ | C45H86O6 |
| LIPID-P-0779 | TG(12:0_15:0_16:0) | GL | TG | 754.69 | 736.66 | [M+NH4]+ | C46H88O6 |
| LIPID-P-0831 | TG(12:0_15:0_18:1) | GL | TG | 780.70 | 762.67 | [M+NH4]+ | C48H90O6 |
| LIPID-P-0782 | TG(14:0_14:0_16:0) | GL | TG | 768.71 | 750.67 | [M+NH4]+ | C47H90O6 |
| LIPID-P-0785 | TG(14:0_15:0_16:0) | GL | TG | 782.72 | 764.69 | [M+NH4]+ | C48H92O6 |
| LIPID-P-0786 | TG(14:0_16:0_16:0) | GL | TG | 796.74 | 778.71 | [M+NH4]+ | C49H94O6 |
| LIPID-P-0787 | TG(15:0_16:0_16:0) | GL | TG | 810.75 | 792.72 | [M+NH4]+ | C50H96O6 |
| LIPID-P-0792 | TG(15:0_16:0_18:0) | GL | TG | 838.78 | 820.75 | [M+NH4]+ | C52H100O6 |
| LIPID-P-0796 | TG(16:0_17:0_18:0) | GL | TG | 866.82 | 848.78 | [M+NH4]+ | C54H104O6 |
| LIPID-P-0784 | TG(12:0_16:0_16:0) | GL | TG | 768.71 | 750.67 | [M+NH4]+ | C47H90O6 |
| LIPID-P-0832 | TG(14:0_15:0_16:1) | GL | TG | 780.70 | 762.67 | [M+NH4]+ | C48H90O6 |
| LIPID-P-0833 | TG(12:0_16:0_18:1) | GL | TG | 794.72 | 776.69 | [M+NH4]+ | C49H92O6 |
| LIPID-P-0834 | TG(14:0_16:0_16:1) | GL | TG | 794.72 | 776.69 | [M+NH4]+ | C49H92O6 |
| LIPID-P-0865 | TG(16:0_24:0_18:1) | GL | TG | 962.91 | 944.88 | [M+NH4]+ | C61H116O6 |
| LIPID-P-0867 | TG(16:0_20:1_22:0) | GL | TG | 962.91 | 944.88 | [M+NH4]+ | C61H116O6 |
| LIPID-P-0868 | TG(16:0_25:0_18:1) | GL | TG | 976.92 | 958.89 | [M+NH4]+ | C62H118O6 |
| LIPID-P-0871 | TG(16:0_26:0_18:1) | GL | TG | 990.94 | 972.91 | [M+NH4]+ | C63H120O6 |
| LIPID-P-0872 | TG(18:0_20:1_22:0) | GL | TG | 990.94 | 972.91 | [M+NH4]+ | C63H120O6 |
| LIPID-P-0874 | TG(8:0_16:1_16:1) | GL | TG | 708.61 | 690.58 | [M+NH4]+ | C43H78O6 |
| LIPID-P-0875 | TG(8:0_14:0_18:2) | GL | TG | 708.61 | 690.58 | [M+NH4]+ | C43H78O6 |
| LIPID-P-0877 | TG(10:0_12:0_18:2) | GL | TG | 708.61 | 690.58 | [M+NH4]+ | C43H78O6 |
| LIPID-P-0862 | TG(16:0_23:0_18:1) | GL | TG | 948.89 | 930.86 | [M+NH4]+ | C60H114O6 |
| LIPID-P-0878 | TG(8:0_16:1_18:1) | GL | TG | 736.65 | 718.61 | [M+NH4]+ | C45H82O6 |
| LIPID-P-0883 | TG(9:0_16:0_18:2) | GL | TG | 750.66 | 732.63 | [M+NH4]+ | C46H84O6 |
| LIPID-P-0884 | TG(14:0_14:1_16:1) | GL | TG | 764.67 | 746.64 | [M+NH4]+ | C47H86O6 |
| LIPID-P-0885 | TG(10:0_16:0_18:2) | GL | TG | 764.67 | 746.64 | [M+NH4]+ | C47H86O6 |
| LIPID-P-0886 | TG(12:0_14:0_18:2) | GL | TG | 764.68 | 746.64 | [M+NH4]+ | C47H86O6 |
| LIPID-P-0890 | TG(15:0_14:1_16:1) | GL | TG | 778.69 | 760.66 | [M+NH4]+ | C48H88O6 |
| LIPID-P-0891 | TG(12:0_15:0_18:2) | GL | TG | 778.69 | 760.66 | [M+NH4]+ | C48H88O6 |
| LIPID-P-0892 | TG(14:1_16:0_16:1) | GL | TG | 792.71 | 774.67 | [M+NH4]+ | C49H90O6 |
| LIPID-P-0893 | TG(12:0_16:0_18:2) | GL | TG | 792.71 | 774.67 | [M+NH4]+ | C49H90O6 |
| LIPID-P-0880 | TG(8:0_16:0_18:2) | GL | TG | 736.64 | 718.61 | [M+NH4]+ | C45H82O6 |
| LIPID-P-0898 | TG(14:0_15:0_18:2) | GL | TG | 806.72 | 788.69 | [M+NH4]+ | C50H92O6 |
| LIPID-P-0861 | TG(15:0_24:0_18:1) | GL | TG | 948.89 | 930.86 | [M+NH4]+ | C60H114O6 |
| LIPID-P-0858 | TG(16:0_18:1_22:0) | GL | TG | 934.88 | 916.85 | [M+NH4]+ | C59H112O6 |
| LIPID-P-0835 | TG(15:0_16:0_16:1) | GL | TG | 808.74 | 790.71 | [M+NH4]+ | C50H94O6 |
| LIPID-P-0836 | TG(14:0_15:0_18:1) | GL | TG | 808.74 | 790.71 | [M+NH4]+ | C50H94O6 |
| LIPID-P-0837 | TG(16:0_16:0_16:1) | GL | TG | 822.76 | 804.72 | [M+NH4]+ | C51H96O6 |
| LIPID-P-0838 | TG(14:0_16:0_18:1) | GL | TG | 822.76 | 804.72 | [M+NH4]+ | C51H96O6 |
| LIPID-P-0840 | TG(15:0_16:0_18:1) | GL | TG | 836.77 | 818.74 | [M+NH4]+ | C52H98O6 |
| LIPID-P-0842 | TG(16:0_16:0_18:1) | GL | TG | 850.79 | 832.75 | [M+NH4]+ | C53H100O6 |
| LIPID-P-0844 | TG(16:0_17:0_18:1) | GL | TG | 864.80 | 846.77 | [M+NH4]+ | C54H102O6 |
| LIPID-P-0847 | TG(16:0_18:0_18:1) | GL | TG | 878.82 | 860.78 | [M+NH4]+ | C55H104O6 |
| LIPID-P-0860 | TG(14:0_20:1_22:0) | GL | TG | 934.88 | 916.85 | [M+NH4]+ | C59H112O6 |
| LIPID-P-0848 | TG(16:0_16:1_20:0) | GL | TG | 878.82 | 860.78 | [M+NH4]+ | C55H104O6 |
| LIPID-P-0850 | TG(17:0_17:0_19:1) | GL | TG | 892.83 | 874.80 | [M+NH4]+ | C56H106O6 |
| LIPID-P-0851 | TG(16:0_18:0_20:1) | GL | TG | 906.85 | 888.81 | [M+NH4]+ | C57H108O6 |
| LIPID-P-0852 | TG(18:0_18:0_18:1) | GL | TG | 906.85 | 888.81 | [M+NH4]+ | C57H108O6 |
| LIPID-P-0853 | TG(16:0_20:0_18:1) | GL | TG | 906.85 | 888.81 | [M+NH4]+ | C57H108O6 |
| LIPID-P-0854 | TG(15:0_16:0_24:1) | GL | TG | 920.86 | 902.83 | [M+NH4]+ | C58H110O6 |
| LIPID-P-0855 | TG(16:0_18:1_21:0) | GL | TG | 920.86 | 902.83 | [M+NH4]+ | C58H110O6 |
| LIPID-P-0856 | TG(18:0_18:1_20:0) | GL | TG | 934.88 | 916.85 | [M+NH4]+ | C59H112O6 |
| LIPID-P-0857 | TG(16:0_24:0_16:1) | GL | TG | 934.87 | 916.85 | [M+NH4]+ | C59H112O6 |
| LIPID-P-0849 | TG(17:0_18:0_18:1) | GL | TG | 892.83 | 874.80 | [M+NH4]+ | C56H106O6 |
| LIPID-P-1021 | TG(18:1_22:0_20:2) | GL | TG | 986.91 | 968.88 | [M+NH4]+ | C63H116O6 |
| LIPID-P-1022 | TG(17:1_18:1_26:1) | GL | TG | 1000.91 | 982.89 | [M+NH4]+ | C64H118O6 |
| LIPID-P-1023 | TG(18:1_18:1_26:1) | GL | TG | 1014.93 | 996.91 | [M+NH4]+ | C65H120O6 |
| LIPID-P-1166 | TG(16:1_16:1_20:5) | GL | TG | 866.72 | 848.69 | [M+NH4]+ | C55H92O6 |
| LIPID-P-1167 | TG(16:2_18:2_18:3) | GL | TG | 866.72 | 848.69 | [M+NH4]+ | C55H92O6 |
| LIPID-P-1168 | TG(15:0_16:1_22:6) | GL | TG | 880.73 | 862.71 | [M+NH4]+ | C56H94O6 |
| LIPID-P-1169 | TG(18:2_18:2_18:3) | GL | TG | 894.76 | 876.72 | [M+NH4]+ | C57H96O6 |
| LIPID-P-1170 | TG(16:0_18:2_20:5) | GL | TG | 894.75 | 876.72 | [M+NH4]+ | C57H96O6 |
| LIPID-P-1171 | TG(16:0_16:1_22:6) | GL | TG | 894.76 | 876.72 | [M+NH4]+ | C57H96O6 |
| LIPID-P-1172 | TG(18:1_18:2_18:4) | GL | TG | 894.75 | 876.72 | [M+NH4]+ | C57H96O6 |
| LIPID-P-1175 | TG(17:0_18:2_20:5) | GL | TG | 908.77 | 890.74 | [M+NH4]+ | C58H98O6 |
| LIPID-P-1176 | TG(18:1_18:2_20:4) | GL | TG | 922.78 | 904.75 | [M+NH4]+ | C59H100O6 |
| LIPID-P-1177 | TG(16:0_18:1_22:6) | GL | TG | 922.79 | 904.75 | [M+NH4]+ | C59H100O6 |
| LIPID-P-1179 | TG(16:1_16:1_24:5) | GL | TG | 922.81 | 904.75 | [M+NH4]+ | C59H100O6 |
| LIPID-P-1180 | TG(17:0_18:1_22:6) | GL | TG | 936.81 | 918.77 | [M+NH4]+ | C60H102O6 |
| LIPID-P-1181 | TG(18:0_18:1_22:6) | GL | TG | 950.82 | 932.78 | [M+NH4]+ | C61H104O6 |
| LIPID-P-1182 | TG(18:1_18:1_22:5) | GL | TG | 950.82 | 932.78 | [M+NH4]+ | C61H104O6 |
| LIPID-P-1183 | TG(18:1_18:2_22:4) | GL | TG | 950.82 | 932.78 | [M+NH4]+ | C61H104O6 |
| LIPID-P-1184 | TG(16:0_20:2_22:5) | GL | TG | 950.82 | 932.78 | [M+NH4]+ | C61H104O6 |
| LIPID-P-1164 | TG(14:0_18:3_20:4) | GL | TG | 866.72 | 848.69 | [M+NH4]+ | C55H92O6 |
| LIPID-P-1187 | TG(18:1_20:1_22:5) | GL | TG | 978.85 | 960.81 | [M+NH4]+ | C63H108O6 |
| LIPID-P-1162 | TG(12:0_18:3_20:4) | GL | TG | 838.69 | 820.66 | [M+NH4]+ | C53H88O6 |
| LIPID-P-1160 | TG(18:1_18:1_24:4) | GL | TG | 980.87 | 962.83 | [M+NH4]+ | C63H110O6 |
| LIPID-P-1244 | TG(18:2_22:5_22:6) | GL | TG | 994.79 | 976.75 | [M+NH4]+ | C65H100O6 |
| LIPID-P-1139 | TG(17:1_18:2_18:3) | GL | TG | 882.74 | 864.72 | [M+NH4]+ | C56H96O6 |
| LIPID-P-1142 | TG(18:1_18:2_18:3) | GL | TG | 896.77 | 878.74 | [M+NH4]+ | C57H98O6 |
| LIPID-P-1144 | TG(16:0_18:2_20:4) | GL | TG | 896.77 | 878.74 | [M+NH4]+ | C57H98O6 |
| LIPID-P-1145 | TG(16:0_16:1_22:5) | GL | TG | 896.77 | 878.74 | [M+NH4]+ | C57H98O6 |
| LIPID-P-1146 | TG(16:0_16:0_22:6) | GL | TG | 896.77 | 878.74 | [M+NH4]+ | C57H98O6 |
| LIPID-P-1149 | TG(16:0_18:1_22:5) | GL | TG | 924.80 | 906.77 | [M+NH4]+ | C59H102O6 |
| LIPID-P-1150 | TG(18:0_18:2_20:4) | GL | TG | 924.80 | 906.77 | [M+NH4]+ | C59H102O6 |
| LIPID-P-1151 | TG(16:0_18:0_22:6) | GL | TG | 924.80 | 906.77 | [M+NH4]+ | C59H102O6 |
| LIPID-P-1152 | TG(18:2_18:2_20:2) | GL | TG | 924.80 | 906.77 | [M+NH4]+ | C59H102O6 |
| LIPID-P-1154 | TG(17:0_18:1_22:5) | GL | TG | 938.81 | 920.78 | [M+NH4]+ | C60H104O6 |
| LIPID-P-1155 | TG(18:1_18:1_22:4) | GL | TG | 952.83 | 934.80 | [M+NH4]+ | C61H106O6 |
| LIPID-P-1156 | TG(18:0_18:0_22:6) | GL | TG | 952.83 | 934.80 | [M+NH4]+ | C61H106O6 |
| LIPID-P-1157 | TG(18:0_18:1_22:5) | GL | TG | 952.83 | 934.80 | [M+NH4]+ | C61H106O6 |
| LIPID-P-1159 | TG(20:0_18:1_22:5) | GL | TG | 980.86 | 962.83 | [M+NH4]+ | C63H110O6 |
| LIPID-P-1161 | TG(20:1_22:1_18:4) | GL | TG | 980.87 | 962.83 | [M+NH4]+ | C63H110O6 |
| LIPID-P-1137 | TG(16:1_18:2_18:3) | GL | TG | 868.74 | 850.71 | [M+NH4]+ | C55H94O6 |
| LIPID-P-1188 | TG(18:0_18:1_24:6) | GL | TG | 978.84 | 960.81 | [M+NH4]+ | C63H108O6 |
| LIPID-P-1194 | TG(14:1_18:2_20:5) | GL | TG | 864.71 | 846.67 | [M+NH4]+ | C55H90O6 |
| LIPID-P-1218 | TG(18:2_20:3_20:4) | GL | TG | 946.79 | 928.75 | [M+NH4]+ | C61H100O6 |
| LIPID-P-1219 | TG(16:0_20:4_22:5) | GL | TG | 946.79 | 928.75 | [M+NH4]+ | C61H100O6 |
| LIPID-P-1220 | TG(20:1_18:2_22:6) | GL | TG | 974.82 | 956.78 | [M+NH4]+ | C63H104O6 |
| LIPID-P-1223 | TG(18:2_18:3_20:5) | GL | TG | 916.74 | 898.71 | [M+NH4]+ | C59H94O6 |
| LIPID-P-1224 | TG(16:1_20:4_20:5) | GL | TG | 916.74 | 898.71 | [M+NH4]+ | C59H94O6 |
| LIPID-P-1226 | TG(18:2_18:2_22:6) | GL | TG | 944.77 | 926.74 | [M+NH4]+ | C61H98O6 |
| LIPID-P-1227 | TG(18:2_20:4_20:4) | GL | TG | 944.77 | 926.74 | [M+NH4]+ | C61H98O6 |
| LIPID-P-1229 | TG(16:0_20:4_22:6) | GL | TG | 944.77 | 926.74 | [M+NH4]+ | C61H98O6 |
| LIPID-P-1230 | TG(18:0_20:4_22:6) | GL | TG | 972.80 | 954.77 | [M+NH4]+ | C63H102O6 |
| LIPID-P-1231 | TG(18:1_20:3_22:6) | GL | TG | 972.80 | 954.77 | [M+NH4]+ | C63H102O6 |
| LIPID-P-1233 | TG(18:2_18:3_22:6) | GL | TG | 942.76 | 924.72 | [M+NH4]+ | C61H96O6 |
| LIPID-P-1236 | TG(18:1_20:4_22:6) | GL | TG | 970.78 | 952.75 | [M+NH4]+ | C63H100O6 |
| LIPID-P-1238 | TG(18:2_20:5_20:5) | GL | TG | 940.74 | 922.71 | [M+NH4]+ | C61H94O6 |
| LIPID-P-1239 | TG(14:0_22:6_22:6) | GL | TG | 940.74 | 922.71 | [M+NH4]+ | C61H94O6 |
| LIPID-P-1240 | TG(16:0_22:6_22:6) | GL | TG | 968.77 | 950.74 | [M+NH4]+ | C63H98O6 |
| LIPID-P-1217 | TG(18:1_18:2_22:6) | GL | TG | 946.79 | 928.75 | [M+NH4]+ | C61H100O6 |
| LIPID-P-1190 | TG(16:0_22:1_22:6) | GL | TG | 978.85 | 960.81 | [M+NH4]+ | C63H108O6 |
| LIPID-P-1216 | TG(18:2_18:3_20:4) | GL | TG | 918.76 | 900.72 | [M+NH4]+ | C59H96O6 |
| LIPID-P-1214 | TG(16:1_16:2_22:6) | GL | TG | 890.72 | 872.69 | [M+NH4]+ | C57H92O6 |
| LIPID-P-1195 | TG(14:1_16:1_22:6) | GL | TG | 864.71 | 846.67 | [M+NH4]+ | C55H90O6 |
| LIPID-P-1196 | TG(18:2_18:3_18:3) | GL | TG | 892.74 | 874.71 | [M+NH4]+ | C57H94O6 |
| LIPID-P-1197 | TG(16:1_16:1_22:6) | GL | TG | 892.73 | 874.71 | [M+NH4]+ | C57H94O6 |
| LIPID-P-1200 | TG(16:0_18:2_22:6) | GL | TG | 920.77 | 902.74 | [M+NH4]+ | C59H98O6 |
| LIPID-P-1201 | TG(16:1_18:1_22:6) | GL | TG | 920.77 | 902.74 | [M+NH4]+ | C59H98O6 |
| LIPID-P-1202 | TG(16:1_16:1_24:6) | GL | TG | 920.77 | 902.74 | [M+NH4]+ | C59H98O6 |
| LIPID-P-1203 | TG(17:0_18:2_22:6) | GL | TG | 934.79 | 916.75 | [M+NH4]+ | C60H100O6 |
| LIPID-P-1204 | TG(18:1_18:2_22:5) | GL | TG | 948.80 | 930.77 | [M+NH4]+ | C61H102O6 |
| LIPID-P-1205 | TG(18:1_18:1_22:6) | GL | TG | 948.80 | 930.77 | [M+NH4]+ | C61H102O6 |
| LIPID-P-1206 | TG(18:0_18:2_22:6) | GL | TG | 948.80 | 930.77 | [M+NH4]+ | C61H102O6 |
| LIPID-P-1207 | TG(18:2_18:2_22:4) | GL | TG | 948.80 | 930.77 | [M+NH4]+ | C61H102O6 |
| LIPID-P-1208 | TG(17:1_17:1_24:6) | GL | TG | 948.80 | 930.77 | [M+NH4]+ | C61H102O6 |
| LIPID-P-1209 | TG(18:1_20:1_22:6) | GL | TG | 976.83 | 958.80 | [M+NH4]+ | C63H106O6 |
| LIPID-P-1210 | TG(18:1_20:3_22:4) | GL | TG | 976.83 | 958.80 | [M+NH4]+ | C63H106O6 |
| LIPID-P-1212 | TG(18:2_18:3_18:4) | GL | TG | 890.73 | 872.69 | [M+NH4]+ | C57H92O6 |
| LIPID-P-1213 | TG(16:0_18:4_20:5) | GL | TG | 890.72 | 872.69 | [M+NH4]+ | C57H92O6 |
| LIPID-P-1215 | TG(16:1_18:2_22:6) | GL | TG | 918.75 | 900.72 | [M+NH4]+ | C59H96O6 |
| LIPID-P-1135 | TG(16:0_16:1_20:5) | GL | TG | 868.74 | 850.71 | [M+NH4]+ | C55H94O6 |
| LIPID-P-1138 | TG(16:0_18:2_18:4) | GL | TG | 868.74 | 850.71 | [M+NH4]+ | C55H94O6 |
| LIPID-P-1132 | TG(12:0_16:0_22:6) | GL | TG | 840.71 | 822.67 | [M+NH4]+ | C53H90O6 |
| LIPID-P-1050 | TG(15:0_19:2_19:2) | GL | TG | 886.79 | 868.75 | [M+NH4]+ | C56H100O6 |
| LIPID-P-1053 | TG(18:1_18:1_18:2) | GL | TG | 900.80 | 882.77 | [M+NH4]+ | C57H102O6 |
| LIPID-P-1054 | TG(16:0_16:1_22:3) | GL | TG | 900.80 | 882.77 | [M+NH4]+ | C57H102O6 |
| LIPID-P-1055 | TG(14:0_20:0_20:4) | GL | TG | 900.80 | 882.77 | [M+NH4]+ | C57H102O6 |
| LIPID-P-1056 | TG(16:0_18:0_20:4) | GL | TG | 900.80 | 882.77 | [M+NH4]+ | C57H102O6 |
| LIPID-P-1057 | TG(17:1_18:1_20:2) | GL | TG | 914.81 | 896.78 | [M+NH4]+ | C58H104O6 |
| LIPID-P-1058 | TG(17:0_19:2_19:2) | GL | TG | 914.82 | 896.78 | [M+NH4]+ | C58H104O6 |
| LIPID-P-1060 | TG(16:0_18:1_22:3) | GL | TG | 928.83 | 910.80 | [M+NH4]+ | C59H106O6 |
| LIPID-P-1062 | TG(18:0_18:1_20:3) | GL | TG | 928.83 | 910.80 | [M+NH4]+ | C59H106O6 |
| LIPID-P-1066 | TG(17:1_18:1_22:2) | GL | TG | 942.84 | 924.81 | [M+NH4]+ | C60H108O6 |
| LIPID-P-1067 | TG(18:1_21:1_18:2) | GL | TG | 942.85 | 924.81 | [M+NH4]+ | C60H108O6 |
| LIPID-P-1069 | TG(18:1_22:1_18:2) | GL | TG | 956.86 | 938.83 | [M+NH4]+ | C61H110O6 |
| LIPID-P-1070 | TG(18:0_18:1_22:3) | GL | TG | 956.86 | 938.83 | [M+NH4]+ | C61H110O6 |
| LIPID-P-1071 | TG(18:1_18:1_22:2) | GL | TG | 956.86 | 938.83 | [M+NH4]+ | C61H110O6 |
| LIPID-P-1134 | TG(15:1_18:2_18:3) | GL | TG | 854.72 | 836.69 | [M+NH4]+ | C54H92O6 |
| LIPID-P-1049 | TG(17:1_18:1_18:2) | GL | TG | 886.79 | 868.75 | [M+NH4]+ | C56H100O6 |
| LIPID-P-1074 | TG(20:1_21:1_18:2) | GL | TG | 970.88 | 952.85 | [M+NH4]+ | C62H112O6 |
| LIPID-P-1048 | TG(16:0_18:2_18:2) | GL | TG | 872.77 | 854.74 | [M+NH4]+ | C55H98O6 |
| LIPID-P-1044 | TG(16:1_18:1_18:2) | GL | TG | 872.77 | 854.74 | [M+NH4]+ | C55H98O6 |
| LIPID-P-1024 | TG(18:1_20:1_24:1) | GL | TG | 1014.94 | 996.91 | [M+NH4]+ | C65H120O6 |
| LIPID-P-1026 | TG(10:0_16:2_18:2) | GL | TG | 760.64 | 742.61 | [M+NH4]+ | C47H82O6 |
| LIPID-P-1027 | TG(14:1_16:1_16:2) | GL | TG | 788.67 | 770.64 | [M+NH4]+ | C49H86O6 |
| LIPID-P-1028 | TG(10:0_18:1_18:3) | GL | TG | 788.67 | 770.64 | [M+NH4]+ | C49H86O6 |
| LIPID-P-1029 | TG(16:1_16:1_16:2) | GL | TG | 816.71 | 798.67 | [M+NH4]+ | C51H90O6 |
| LIPID-P-1030 | TG(14:1_16:1_18:2) | GL | TG | 816.71 | 798.67 | [M+NH4]+ | C51H90O6 |
| LIPID-P-1031 | TG(12:0_18:2_18:2) | GL | TG | 816.71 | 798.67 | [M+NH4]+ | C51H90O6 |
| LIPID-P-1032 | TG(12:0_18:1_18:3) | GL | TG | 816.71 | 798.67 | [M+NH4]+ | C51H90O6 |
| LIPID-P-1033 | TG(14:0_16:1_18:3) | GL | TG | 816.71 | 798.67 | [M+NH4]+ | C51H90O6 |
| LIPID-P-1034 | TG(16:1_17:1_16:2) | GL | TG | 830.72 | 812.69 | [M+NH4]+ | C52H92O6 |
| LIPID-P-1035 | TG(15:0_16:1_18:3) | GL | TG | 830.73 | 812.69 | [M+NH4]+ | C52H92O6 |
| LIPID-P-1036 | TG(14:0_18:2_18:2) | GL | TG | 844.74 | 826.71 | [M+NH4]+ | C53H94O6 |
| LIPID-P-1038 | TG(16:1_16:1_18:2) | GL | TG | 844.74 | 826.71 | [M+NH4]+ | C53H94O6 |
| LIPID-P-1040 | TG(14:0_16:0_20:4) | GL | TG | 844.74 | 826.71 | [M+NH4]+ | C53H94O6 |
| LIPID-P-1041 | TG(16:0_16:1_18:3) | GL | TG | 844.74 | 826.71 | [M+NH4]+ | C53H94O6 |
| LIPID-P-1042 | TG(15:0_18:2_18:2) | GL | TG | 858.76 | 840.72 | [M+NH4]+ | C54H96O6 |
| LIPID-P-1043 | TG(16:1_17:1_18:2) | GL | TG | 858.75 | 840.72 | [M+NH4]+ | C54H96O6 |
| LIPID-P-1046 | TG(16:0_16:0_20:4) | GL | TG | 872.77 | 854.74 | [M+NH4]+ | C55H98O6 |
| LIPID-P-1075 | TG(18:1_24:1_18:2) | GL | TG | 984.90 | 966.86 | [M+NH4]+ | C63H114O6 |
| LIPID-P-1073 | TG(17:1_18:1_24:2) | GL | TG | 970.88 | 952.85 | [M+NH4]+ | C62H112O6 |
| LIPID-P-1078 | TG(21:1_22:1_18:2) | GL | TG | 998.91 | 980.88 | [M+NH4]+ | C64H116O6 |
| LIPID-P-1076 | TG(16:1_18:1_26:2) | GL | TG | 984.89 | 966.86 | [M+NH4]+ | C63H114O6 |
| LIPID-P-1106 | TG(16:1_18:1_20:3) | GL | TG | 898.79 | 880.75 | [M+NH4]+ | C57H100O6 |
| LIPID-P-1108 | TG(17:0_18:1_20:4) | GL | TG | 912.80 | 894.77 | [M+NH4]+ | C58H102O6 |
| LIPID-P-1109 | TG(19:1_18:2_18:2) | GL | TG | 912.80 | 894.77 | [M+NH4]+ | C58H102O6 |
| LIPID-P-1111 | TG(18:1_18:2_20:2) | GL | TG | 926.81 | 908.78 | [M+NH4]+ | C59H104O6 |
| LIPID-P-1113 | TG(18:0_18:3_20:2) | GL | TG | 926.82 | 908.78 | [M+NH4]+ | C59H104O6 |
| LIPID-P-1114 | TG(16:0_18:1_22:4) | GL | TG | 926.82 | 908.78 | [M+NH4]+ | C59H104O6 |
| LIPID-P-1116 | TG(18:0_18:1_20:4) | GL | TG | 926.82 | 908.78 | [M+NH4]+ | C59H104O6 |
| LIPID-P-1117 | TG(18:2_18:2_20:1) | GL | TG | 926.82 | 908.78 | [M+NH4]+ | C59H104O6 |
| LIPID-P-1122 | TG(18:2_18:2_22:1) | GL | TG | 954.85 | 936.81 | [M+NH4]+ | C61H108O6 |
| LIPID-P-1123 | TG(18:1_18:2_22:2) | GL | TG | 954.84 | 936.81 | [M+NH4]+ | C61H108O6 |
| LIPID-P-1124 | TG(18:0_18:1_22:4) | GL | TG | 954.85 | 936.81 | [M+NH4]+ | C61H108O6 |
| LIPID-P-1125 | TG(16:0_18:1_24:4) | GL | TG | 954.84 | 936.81 | [M+NH4]+ | C61H108O6 |
| LIPID-P-1126 | TG(24:1_18:2_18:2) | GL | TG | 982.88 | 964.85 | [M+NH4]+ | C63H112O6 |
| LIPID-P-1127 | TG(14:1_18:2_18:3) | GL | TG | 840.70 | 822.67 | [M+NH4]+ | C53H90O6 |
| LIPID-P-1131 | TG(14:0_16:1_20:5) | GL | TG | 840.71 | 822.67 | [M+NH4]+ | C53H90O6 |
| LIPID-P-1104 | TG(18:1_18:2_18:2) | GL | TG | 898.79 | 880.75 | [M+NH4]+ | C57H100O6 |
| LIPID-P-1102 | TG(15:0_18:1_20:4) | GL | TG | 884.77 | 866.74 | [M+NH4]+ | C56H98O6 |
| LIPID-P-1105 | TG(16:0_18:1_20:4) | GL | TG | 898.79 | 880.75 | [M+NH4]+ | C57H100O6 |
| LIPID-P-1099 | TG(14:0_16:0_22:5) | GL | TG | 870.76 | 852.72 | [M+NH4]+ | C55H96O6 |
| LIPID-P-1079 | TG(20:1_24:1_18:2) | GL | TG | 1012.93 | 994.89 | [M+NH4]+ | C65H118O6 |
| LIPID-P-1080 | TG(14:1_16:1_16:3) | GL | TG | 786.66 | 768.63 | [M+NH4]+ | C49H84O6 |
| LIPID-P-1081 | TG(16:0_16:2_16:3) | GL | TG | 814.69 | 796.66 | [M+NH4]+ | C51H88O6 |
| LIPID-P-1082 | TG(12:0_18:2_18:3) | GL | TG | 814.69 | 796.66 | [M+NH4]+ | C51H88O6 |
| LIPID-P-1083 | TG(14:1_16:1_18:3) | GL | TG | 814.69 | 796.66 | [M+NH4]+ | C51H88O6 |
| LIPID-P-1101 | TG(17:1_18:2_18:2) | GL | TG | 884.77 | 866.74 | [M+NH4]+ | C56H98O6 |
| LIPID-P-1086 | TG(16:1_16:1_18:3) | GL | TG | 842.72 | 824.69 | [M+NH4]+ | C53H92O6 |
| LIPID-P-1087 | TG(14:0_18:2_18:3) | GL | TG | 842.72 | 824.69 | [M+NH4]+ | C53H92O6 |
| LIPID-P-1088 | TG(14:1_18:2_18:2) | GL | TG | 842.73 | 824.69 | [M+NH4]+ | C53H92O6 |
| LIPID-P-1085 | TG(15:0_16:1_18:4) | GL | TG | 828.71 | 810.67 | [M+NH4]+ | C52H90O6 |
| LIPID-P-1090 | TG(14:0_16:1_20:4) | GL | TG | 842.72 | 824.69 | [M+NH4]+ | C53H92O6 |
| LIPID-P-1092 | TG(15:0_18:2_18:3) | GL | TG | 856.74 | 838.71 | [M+NH4]+ | C54H94O6 |
| LIPID-P-1094 | TG(16:1_17:1_18:3) | GL | TG | 856.74 | 838.71 | [M+NH4]+ | C54H94O6 |
| LIPID-P-1095 | TG(16:1_18:2_18:2) | GL | TG | 870.75 | 852.72 | [M+NH4]+ | C55H96O6 |
| LIPID-P-1096 | TG(16:0_18:2_18:3) | GL | TG | 870.75 | 852.72 | [M+NH4]+ | C55H96O6 |
| LIPID-P-1097 | TG(16:1_18:1_18:3) | GL | TG | 870.75 | 852.72 | [M+NH4]+ | C55H96O6 |
| LIPID-P-1098 | TG(16:0_16:1_20:4) | GL | TG | 870.76 | 852.72 | [M+NH4]+ | C55H96O6 |
| LIPID-P-1089 | TG(12:0_16:0_22:5) | GL | TG | 842.73 | 824.69 | [M+NH4]+ | C53H92O6 |
| LIPID-P-0772 | TG(8:0_16:0_16:0) | GL | TG | 712.64 | 694.61 | [M+NH4]+ | C43H82O6 |
| LIPID-P-1903 | TG(O-20:0_18:0_22:5) | GL | TG-O | 968.90 | 950.87 | [M+NH4]+ | C63H114O5 |
| LIPID-P-1890 | TG(O-16:0_16:0_20:4) | GL | TG-O | 858.79 | 840.76 | [M+NH4]+ | C55H100O5 |
| LIPID-P-1870 | TG(O-20:0_16:0_18:2) | GL | TG-O | 890.85 | 872.82 | [M+NH4]+ | C57H108O5 |
| LIPID-P-1855 | TG(O-20:0_16:0_18:1) | GL | TG-O | 892.87 | 874.84 | [M+NH4]+ | C57H110O5 |
