# Supplementary material for: Anti-obesity effects of Wumeishanzhayin: an integrated lipidomics and transcriptomics study
Source: Front Pharmacol. 2025 Oct 21;16:1697683. doi: 10.3389/fphar.2025.1697683 (PMC12584145; doi:10.3389/fphar.2025.1697683)
Supplement: Supplementary file 2 [file Supplementaryfile2.docx]

Supplementary Table 2

Specific internal standard situation

| **NO.** | **Glass** | **Material name** | **CAS** | **manufacturer** | **Item Number** |
| --- | --- | --- | --- | --- | --- |
| 1 | PC | PC(16:0/16:0)-d9 | 77165-56-1 | Avanti | 860352P |
| 2 | Eicosanoid | 5S-HETE-d8 | 330796-62-8 | Cayman | 334230 |
| 3 | FFA | Arachidonic Acid-d8 | 69254-37-1 | Cayman | 390010 |
| 4 | CAR | CAR(16:0)-d3 | 202480-73-7 | sigma | 55107 |
| 5 | CE | CE(18:1)-d7 | 1416275-35-8 | Avanti | 700185M |
| 6 | Cer | Cer(d18:1/15:0)-d7 | — | Avanti | 860681P |
| 7 | Cer | Cer(d18:1-d7/16:0) | — | Avanti | 860676P |
| 8 | Cer | Cer(d18:1-d7/18:0) | — | Avanti | 860677P |
| 9 | Cer | Cer(d18:1-d7/24:0) | 1840942-15-5 | Avanti | 860678P |
| 10 | Cer | Cer(d18:1-d7/24:1) | 54164-50-0 | Avanti | 860525P |
| 11 | Cert | Cer(t18:0/22:0-d3) | 2011762-87-9 | Cayman | 9003465 |
| 12 | CerP | CerP(d18:1/8:0) | 474943-70-9 | Avanti | 860532P |
| 13 | Cho | cholesterol-d7 | 83199-47-7 | sigma | 700041P |
| 14 | CoQ | CoQ10-d9 | 303-98-0 | Avanti | 802891 |
| 15 | DG | DG(17:0/17:0)-d5 | — | Avanti | 800854P |
| 16 | FFA | FFA(16:0)-d31 | 39756-30-4 | Supelco | 68277 |
| 17 | BA | GCDCA-d4 | 1201918-16-2 | sigma | 739715 |
| 18 | HexCer | HexCer(d18:1-d5/18:0) | 2260670-13-9 | Avanti | 860638P |
| 19 | LPA | LPA(17:0) | 799268-66-9 | Avanti | 857127P |
| 20 | LPC | LPC(15:0)-d5 | 2342574-95-0 | Avanti | 870309L |
| 21 | LPC | LPC(16:0)-d31 | 327178-91-6 | Avanti | 860397P |
| 22 | LPC | LPC(17:0)-d5 | 2342575-12-4 | Avanti | 855679L |
| 23 | LPC | LPC(18:1-d7) | 2097561-13-0 | zzstandard | IR-54029 |
| 24 | LPE | LPE(15:0)-d5 | 2342574-96-1 | Avanti | 856709L |
| 25 | LPE | LPE(18:1-d7) | 2260669-47-2 | zzstandard | IR-54035 |
| 26 | LPG | LPG(15:0)-d5 | 2342574-98-3 | zzstandard | IR-54057 |
| 27 | LPG | LPG(17:1) | 1246298-11-2 | zzstandard | ZL-20103 |
| 28 | LPI | LPI(17:1) | 1246353-39-8 | Avanti | 850103P |
| 29 | LPS | LPS(17:1) | 1246298-15-6 | Avanti | 858141P |
| 30 | MG | MG(18:1-d7) | 2260669-49-4 | Avanti | 791646C |
| 31 | PA | PA(17:0/17:0) | 154804-54-3 | Avanti | 830856P |
| 32 | PC | PC(15:0/18:1(d7)) | 2097561-16-3 | zzstandard | IR-54021 |
| 33 | PC | PC(16:0-d31/18:1) | 179093-76-6 | Avanti | 860399P |
| 34 | PC | PC(14:0/14:0)-d9 | 71479-88-4 | Avanti | 860342P |
| 35 | PE | PE(15:0/18:1(d7)) | 2097561-15-2 | zzstandard | IR-54033 |
| 36 | PE | PE(16:0-d31/18:1) | 326495-44-7 | Avanti | 860374P |
| 37 | PE | PE(17:0/14:1)-d5 | 2342575-78-2 | Avanti | 856721L |
| 38 | PE | PE(17:0/18:1)-d5 | 2342575-56-6 | Avanti | 856719L |
| 39 | PE | PE(17:0-22:4)-d5 | 2342575-44-2 | zzstandard | IR-54054 |
| 40 | PG | PG(15:0/18:1(d7)) | — | Avanti | 791640C |
| 41 | PG | PG(16:0/d31/18:1) | 327178-87-0 | Avanti | 860384P |
| 42 | PI | PI(16:0-d31/18:1) | 799812-61-6 | Avanti | 860042P |
| 43 | PMeOH | PMeOH(16:0/16:0) | 92609-89-7 | zzstandard | ZL-20797 |
| 44 | PS | PS(15:0/18:1(d7)) | — | Avanti | 791639C |
| 45 | PS | PS(16:0(d31)/18:1) | 327178-96-1 | Avanti | 860403P |
| 46 | PS | PS(16:0/16:0)-d9 | 28152 | cayman | 28152 |
| 47 | SM | SM(d18:1/18:1)-d9 | 2260669-50-7 | Avanti | 791649C |
| 48 | SM | SM(d18:1/20:1)-d9 | 2342574-48-3 | Avanti | 860742L |
| 49 | SM | SM(d18:1/22:1)-d9 | 2342574-51-8 | Avanti | 860743L |
| 50 | SM | SM(d18:1-d9/15:0) | — | Avanti | 860686P |
| 51 | SPH | SPH(18:1)-d7 | 1246304-34-6 | Avanti | 860657P |
| 52 | TG | TG(14:0/16:1/14:0)-d5 | 944709-23-3 | zzstandard | IR-54066 |
| 53 | TG | TG(16:0-15:1-16:0)-d5 | 2342574-85-8 | Avanti | 860910L |
| 54 | TG | TG(17:0/17:1/17:0)-d5 | 958760-74-2 | Avanti | 860903P |
